# Rapid quantitation of erythropoietin and identification of its glycans using membranes for capture and digestion

**DOI:** 10.1016/j.talanta.2026.129396

**Published:** 2026-01-12

**Authors:** Yuhang Chen, Bill Boggess, Junyan Yang, Nicholas E. Manicke, Merlin L. Bruening

**Affiliations:** a Department of Chemistry and Biochemistry, University of Notre Dame, Notre Dame, IN, 46556, United States; b Department of Chemical and Biomolecular Engineering, University of Notre Dame, Notre Dame, IN, 46556, United States; c Department of Chemistry and Chemical Biology, Indiana University Indianapolis, Indianapolis, IN 46202, United States

**Keywords:** Affinity peptides, Erythropoietin, Fluorescence assay, Glycosylation, LC-MS/MS

## Abstract

Erythropoietin (EPO) is a glycoprotein hormone used to treat chronic anemia. Glycans at the three N-glycosylation sites strongly influence EPO’s stability and bioactivity, so accurate quantitation of EPO and analysis of its glycosylation patterns are critical during production and subsequent processing. This work demonstrates a rapid workflow for EPO capture, quantitation, and glycan profiling. Newly developed porous membranes containing affinity peptides selectively capture EPO from cell culture supernatants. In a 96-well-plate format, captured EPO was labeled with a fluorescent antibody to enable quantitation in a 10-min assay with an average coefficient of variation of 12 %. Elution of captured EPO from a 2-cm glass-fiber membrane combined with tryptic membranes enabled efficient purification and digestion for liquid chromatography–tandem mass spectrometry (LC–MS/MS) glycan analysis. Without purification, conventional LC-MS/MS identification of EPO N-glycans in Chinese hamster ovary (CHO) cell supernatant was not possible. The rapid in-membrane digestion yielded glycan profiles comparable to those obtained from overnight in-solution digestion. Thus, the membrane-based assays provide a novel approach for rapid EPO quantitation and facilitating glycan identification.

## 1. Introduction

This study employs new affinity membranes that capture erythropoietin (EPO) from CHO cell supernatant in minutes to facilitate rapid quantitation of EPO and subsequent analysis of its glycosylation pattern. EPO is a glycoprotein hormone that regulates red blood cell production. Synthesized mainly in the kidneys in response to hypoxic conditions, EPO stimulates bone marrow to increase red blood cell production, thereby enhancing the blood’s oxygen-carrying capacity [1]. Recombinant human erythropoietin (rhEPO) has emerged as an important treatment for anemia patients with chronic kidney disease, cancer and HIV/AIDS [2]. Since its approval in the late 1980s, rhEPO has reduced the need for blood transfusions and improved the quality of life in patients suffering from chronic anemia [3,4].

Current protocols for determining the concentration of rhEPO include Enzyme-Linked Immunosorbent Assays (ELISAs), optical or electrochemical detection, and liquid chromatography-tandem mass spectrometry (LC-MS/MS) [5–11]. Noea et al. developed a sandwich immunoassay that employs antibodies against two different epitopes to quantify EPO in human serum [12]. Other studies also demonstrated EPO immunoassays [13–15]. Despite their widespread application, ELISAs typically require more than 2 h and sometimes overnight, limiting throughput [16–18]. Ko et al. created an immunoassay that uses a camelid single-domain antibody to bind rhEPO and bio-layer interferometry for label-free detection [19]. Their assay allows quantitation of EPO in 80 Chinese hamster ovary (CHO) cell samples within 45 min. However, the single-domain antibody is expensive, and the 45-min turnaround time still has room for optimization. While immunoassays are highly specific and sensitive, such sensitivity is not required for rhEPO determination in production streams, where concentrations may reach hundreds of micrograms per milliliter [15]. Consequently, samples must be diluted more than 10,000-fold prior to analysis [15].

High-affinity peptides offer advantages over antibodies for affinity capture in some applications. Chemical synthesis of peptides enables their production at a lower cost compared to antibodies [20,21], and the relatively small size of peptides enhances tissue penetration when they serve as drugs or imaging probes [21]. Recent studies employed affinity peptides for protein capture [22–25]. Peptide immobilization can occur at a higher density than antibody immobilization to potentially enhance target capture through multiple interactions. Yang and coworkers showed that membranes with affinity peptides capture monoclonal antibodies more efficiently than membranes with Protein A [23]. This work explores the use of affinity peptides in new porous membranes for EPO capture.

Affinity membranes are attractive for rapid capture of specific biomolecules. Our research group developed several methods for quantifying protein concentrations using membranes functionalized with highly specific peptide ligands [24,26–28]. Flow in membrane pores delivers proteins to ligands immobilized on the pore surface, enabling rapid and efficient capture in residence times as short as milliseconds [29]. After target binding, capture of a fluorescently labeled secondary binder affords quantitation in minutes.

Glycosylation is a critical post-translational modification of EPO that influences its biological activity, stability, and pharmacokinetics [6, 30–32]. CHO cells are the most widely used expression system for rhEPO production because they can perform post-translational modifications, such as glycosylation, that are essential for EPO’s biological function [30,33]. Human EPO contains three N-linked glycosylation sites and one O-linked glycosylation site, with glycans contributing nearly 40 % of its molecular mass [34,35]. The N-glycans on EPO modulate its solubility, protect against proteolytic degradation, and extend its half-life [33,36]. Moreover, N-linked glycosylation patterns affect EPO’s immunogenicity and are a key determinant of its clinical efficacy [37]. Consequently, N-glycan identification on EPO is indispensable to ensure batch consistency and regulatory compliance [38,39]. Proteolytic digestion of glycoproteins followed by LC-MS/MS is a key method for identifying glycosylation sites and characterizing glycan compositions [40,41].

Analysis of EPO glycans typically employs purified protein, and purification of EPO from CHO cell supernatant may require hours of affinity-based enrichment or cumbersome chromatography [39,42–44]. In contrast, our affinity membranes enable selective EPO capture and elution within minutes. Moreover, tryptic digestion prior to LC-MS/MS may take hours, substantially extending the overall turnaround time for glycopeptide analysis. Using enzyme-functionalized membranes, our group achieved rapid protein digestion within minutes [23,26,45]. By combining protease-functionalized membranes with EPO capture in affinity membranes, we may expedite site-specific monitoring of glycans during EPO production.

This work describes the fabrication and use of membranes modified with an affinity peptide that captures EPO. Martínez-Ceron et al. reported that Ac-FHHFAHAG selectively binds to EPO with a dissociation constant, K_D_, of 1.5 μM [46], so we immobilized this peptide in membranes using SGSGK as a linker. The resulting affinity membranes capture EPO during flow of CHO-cell supernatant through the membrane. When using membranes in 96-well plates, binding of a fluorescently labeled secondary antibody to immobilized EPO enables quantitation of this protein in 10 min. A subsequent protease-containing membrane catalyzes rapid digestion of EPO eluted from an affinity membrane to streamline site-specific N-glycan identification. The Ac-FHHFAHAGSGSGK (EP13)-functionalized membrane facilitates both EPO quantification and subsequent glycan identification, highlighting its potential for monitoring EPO during production.

## 2. Experimental

### 2.1. Materials

Glass fiber membranes (A/C Glass Fiber 1 μm pores, 279 μm thick, 25 mm diameter, cat #66213) and AcroPrep Advance 96-well plates (350 μL well volume, each well contains an 8 mm diameter, 660 μm thick glass-fiber membrane with 1 μm pores, cat #8031) were acquired from Pall Corporation. The 25-mm glass fiber membrane was cleaned in a UV/O3 chamber (Jelight, model18) prior to modification.

Polyethyleneimine (PEI, M_W_ = 25 kDa), poly (acrylic acid) (PAA, M_W_ = 250 kDa, 35 % in aqueous solution), N-hydroxysuccinimide (NHS), Trypsin (TPCK-treated, lyophilized powder, ≥10,000 BAEE units per mg of protein), poly(sodium 4-styrenesulfonate) (PSS, average molecular weight ~70,000 Da), Tween 20, sodium chloride, sodium hydroxide, urea, sodium dodecyl sulfate, Tris(hydroxymethyl) aminomethane, glycine, sodium phosphate monobasic monohydrate, sodium phosphate dibasic heptahydrate, dithiothreitol (DTT) and 2-bromoethylamine were purchased from Sigma-Aldrich. 1-Ethyl-3-(3-dimethylaminopropyl) carbodiimide hydrochloride (EDC), DyLight 650-labeled goat anti-rabbit secondary antibody and bovine serum albumin (BSA) were purchased from Thermo Fisher Scientific. Rabbit anti-human EPO antibody was purchased from Bio-Techne. EPO affinity peptide (Ac-FHHFAHAGSGSGK, EP13) and recombinant EPO were purchased from Genscript. Sodium dodecyl sulfate polyacrylamide gel electrophoresis (SDS-PAGE) gels and protein ladders were purchased from BIO-RAD. CHO cell culture supernatants were prepared previously [23]. Solutions were prepared using deionized water (Milli-Q, 18.2 MΩ cm).

### 2.2. Immobilization of EP13 in glass fiber membranes in 96-well plates

Immobilization of affinity peptides in glass-fiber membranes in 96-well plates includes three steps: adsorption of a multilayer polyelectrolyte film containing –COOH groups, chemical activation of these groups using EDC/NHS chemistry, and covalent attachment of the affinity ligands to the membrane via reaction of peptide amine groups with the succinimidyl active ester. First, 100 μL of 2 mg/mL branched PEI (polyethylenimine) at pH 3 was added to each well and incubated for 5 min. Following incubation, the solutions were pulled through the glass-fiber membranes using a vacuum manifold. This procedure was repeated three additional times with fresh PEI solutions to increase the PEI coverage, and afterward the wells were washed with 1 mL of water with vacuum on. Then, 100 μL of 1.1 mg/mL PAA (polyacrylic acid) in 0.5 M NaCl at pH 3 was added to each well and pulled through the membrane after a 5-min incubation. The PAA incubation was repeated three additional times, and afterward membranes were rinsed with 1 mL of DI water with vacuum on. Additional PEI and PAA layers were deposited similarly to give two PEI/PAA bilayers. To activate the carboxylic acid groups in the adsorbed (PEI/PAA)_2_ films, 100 μL of a solution containing 0.1 M EDC and 0.1 M NHS was added to each well and incubated for 10 min. After pulling the solution through the membranes under vacuum, this incubation step was repeated five additional times to ensure activation of essentially all the –COOH groups. Following these incubations, each membrane was rinsed with 1 mL of DI water.

Finally, to covalently react the activated –COOH groups with EP13, 200 μL of 200 μg/mL EP13 in 20 mM phosphate containing 150 mM NaCl (pH 7.4, Buffer A) was added to each well and incubated for 10 min. The solution was then drawn through the membrane, followed by five additional incubations with fresh EP13 solution. After that, the membranes in each well were rinsed with 1 mL of Buffer A with vacuum on. The supplementary material describes the related procedure for modifying 25 mm-diameter glass-fiber membranes using flow methods. The O-ring holder in that procedure restricts the modification to an exposed 2-cm diameter [26].

### 2.3. Measurement of EPO breakthrough curves in peptide-modified 2-cm membranes

EPO was spiked in 20 mM phosphate (no salts, pH = 4, Buffer B). The developer of the affinity peptide claims it captures EPO more specifically at pH = 4 [46]. Six mL of 25 μg/mL EPO in Buffer B was passed through the EP13-modified membrane at 1 mL/min. In a control experiment to demonstrate that the peptide is responsible for EPO binding, 4 mL of 25 μg/mL EPO in Buffer B was passed through a membrane modified with ethanolamine (without the affinity peptide) at the same flow rate. (Modification of membranes with ethanolamine and peptides employs the same procedure; both react amine groups with active esters in the membrane to limit covalent protein binding, but the ethanol amine will not have affinity for EPO.) In a separate control experiment, 4 mL of 1:800 diluted CHO-cell supernatant (containing ~25 μg/mL host-cell proteins in Buffer B) was passed through an EP13-modified membrane at 1 mL/min. Diluted supernatant was employed to enable detection of adsorbed protein by difference. Permeates were collected in pre-weighed tubes, and their volumes were calculated from the mass increases after permeate collection. The EPO concentration in each aliquot was quantified by measuring intrinsic tryptophan fluorescence with a Synergy H1 microplate reader. The host cell protein concentration in undiluted CHO-cell supernatant was 20 ± 3 mg/mL as determined by precipitation using organic solvent. The supernatant was mixed with acetone at a 1:4 (v/v) ratio in a preweighed centrifuge tube, incubated at −20 °C overnight, and then centrifuged to pellet the precipitated proteins. After decanting the supernatant, the resulting pellet was dried under a nitrogen stream and weighed together with the centrifuge tube to determine the protein mass.

### 2.4. Binding and quantitation of EPO in 96-well plates containing membranes modified with EP13

**Fig. 1.**
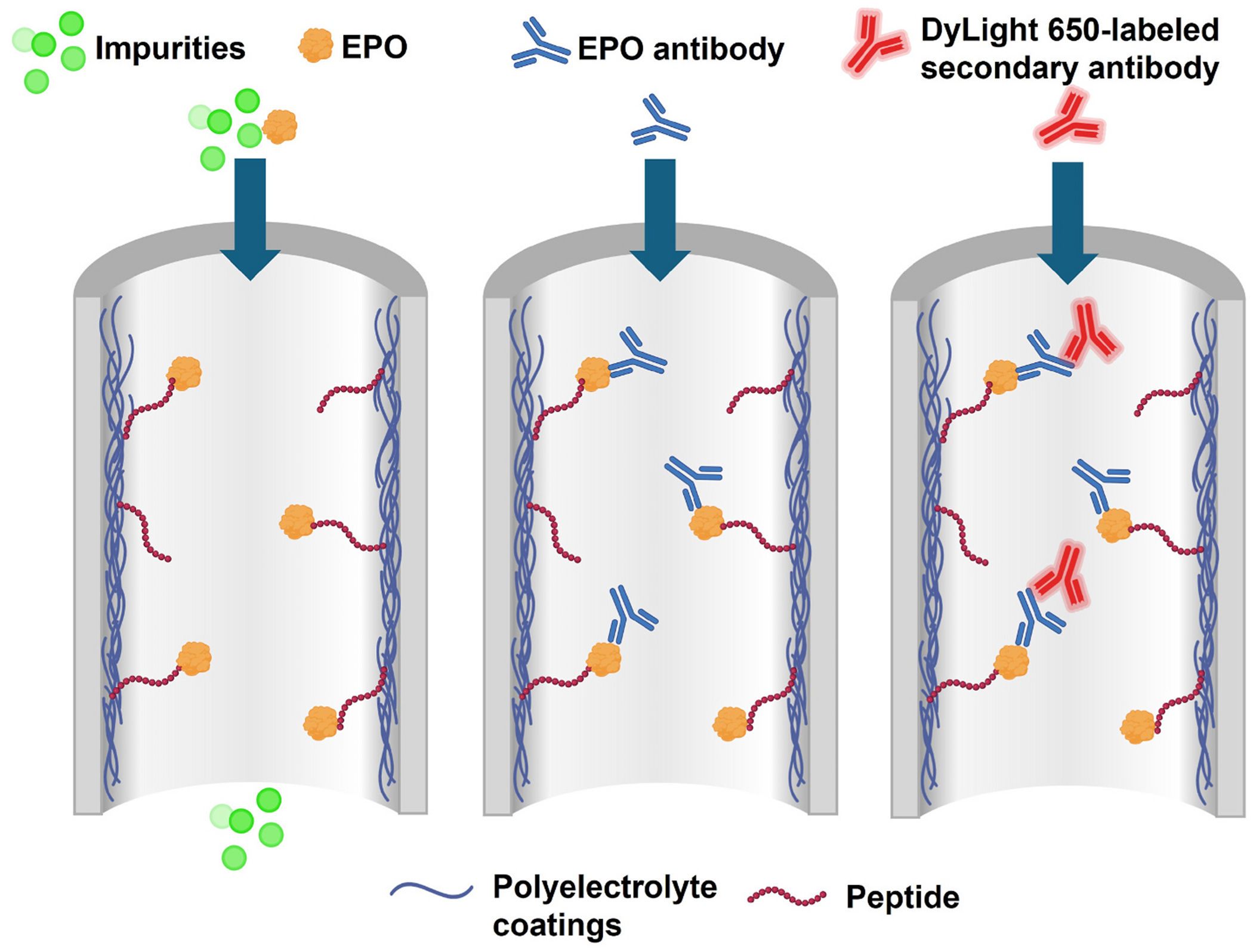
Schematic workflow for EPO quantitation using a 96-well plate. Glass-fiber membranes were modified with an affinity peptide to selectively capture EPO, followed by binding of an EPO antibody and a fluorescent secondary antibody. EPO concentration was determined based on fluorescence intensity and calibration curves. Rinsing occurred after each step.

A series of EPO standards (0, 0.5, 1, 2, 4, 6, 8, and 10 μg/mL in Buffer B) were prepared by diluting from a stock solution. Fig. 1 shows the workflow for quantitation using a 96-well plate. At a flow rate of 0.5 mL/min, 0.5 mL of the EPO standard was passed through the wells, followed by a 1 mL rinse with Buffer A to remove nonspecifically bound protein. (The flow rate was controlled by adjusting the vacuum.) Then, 0.5 mL of 10 μg/mL rabbit anti-human EPO IgG in 20 mM phosphate containing 500 mM NaCl (pH 7.4, Buffer C) was passed through each membrane. After the solutions were pulled through, the membranes were rinsed with 1 mL of Buffer C, and 0.5 mL of 10 μg/mL DyLight 650-labeled goat anti-rabbit secondary antibody in Buffer C was passed through each well. Then, 2 mL of 20 mM phosphate containing 500 mM NaCl with 0.1 % Tween20 (pH 7.4) was added to remove nonspecifically bound materials under full vacuum. Afterward, the plate was sealed with parafilm and placed on the vacuum manifold at maximum vacuum for 30 s to remove excess moisture from the glass-fiber membranes. The 96-well plate was placed in the Synergy H1 microplate reader for fluorescence measurement using an excitation wavelength of 650 nm and an emission wavelength of 675 nm. The calibration curve was established based on the fluorescence intensities of EPO standards in buffer B.

To evaluate the protocol under conditions similar to those used in manufacturing, we spiked EPO standards into 1:10 (v/v) CHO-cell supernatant (from a 10^10^ cell/L culture) diluted in Buffer B and supplemented with 10 μg/mL BSA as a further protein contaminant [46]. The diluted CHO-cell supernatant contained 1.8 ± 0.3 mg/mL host-cell protein. The solutions were passed through the modified membranes, followed by binding of primary and secondary antibodies using the above procedure. The membranes were then inserted into the plate reader for fluorescence measurement, as described above.

### 2.5. Binding and elution of EPO in EP13-Modified 2-cm membranes

CHO-cell supernatant was diluted 1:4 (v:v) in buffer B. EPO was spiked into 1 mL of diluted CHO-cell supernatant, and the solution was passed through a 2-cm EP13-modified glass-fiber membrane at 1 mL/min and collected in a tube. Subsequently, 10 mL of Buffer C was passed through the membrane at 1 mL/min, followed by a 10 mL deionized water rinse at the same flow rate. Finally, two 0.5-mL aliquots of 1 mg/mL aqueous SDS were circulated at 1 mL/min for 2 min each to elute EPO. The eluted EPO was collected in a centrifuge tube and stored for further use. For gel electrophoresis characterization, one hundred microliters of feed and flow-through solution and 500 μL of SDS eluate were dried in a SpeedVac vacuum concentrator and reconstituted in 10 μL of bromophenol blue-containing SDS-PAGE loading dye [47]. Then, they were loaded into a 4–20 % gradient SDS-PAGE (polyacrylamide gel electrophoresis) gel.

### 2.6. Characterization of glycopeptides using LC-MS/MS

EPO was denatured, reduced, and alkylated with 2-bromoethylamine prior to tryptic digestion. Digestion was then performed either in solution or by passing the sample through a trypsin-modified nylon membrane (see sections S1 and S2 and Fig. S1 in the supporting information for details of protein pretreatment and digestion). One μL of digested EPO (in aqueous 4 % acetonitrile, 0.1 % formic acid) was injected for LC-MS/MS analysis. Peptide separation was performed on a 75 mm × 100 μm peptide BEH C18 column (Waters, Billerica, MA) at a flow rate of 600 nL/min with a temperature of 53 °C using a 48 min gradient from 4 % B for 8.1 min, 4–7 % B 8.1–10.0 min, 7–33 % B 10–30 min, 33–90 % B 30–33 min, 90 % B for 3 min, 90-4 % B 36–37 min, and 4 % B 37.1–48 min to equilibrate the column. Mobile phase A consisted of water with 0.1 % formic acid (Honeywell/Burdick & Jackson, Muskegon, MI), and mobile phase B was acetonitrile with 0.1 % formic acid. A Sciex (Framingham, MA) nanoESI probe mounted on a Sciex Opti-flow^®^ ion source was used to introduce eluant from a Waters M-Class nano ultrahigh pressure liquid chromatography system into a Sciex ZenoTOF 7600 mass spectrometer for analysis. Ion source interface parameters were as follows: spray voltage = 3200 V; nano gas 1 = 10 psi; curtain gas = 25 psi; nano cell temperature = 225 °C; declustering potential = 80 V; declustering potential spread = 0 V. The MS1 acquisition parameters were: TOF mass range = 400–2000 Da; accumulation time = 0.1 s. The fragmentation mode was set as CID. For MS2, the parameters were: TOF mass range = 100–2000 Da; accumulation time = 0.025 s; dynamic collision energy based on the charge state and *m/z* value of the precursor ion; collision energy spread = 5 V; dynamic exclusion of precursor ions for 10 s after 2 occurrences. The Zeno threshold was set to 100,000 cps. Raw data were processed using Sciex OS software.

### 2.7. Data analysis

The LC-MS/MS data were analyzed using Glyco-Decipher [48]. The fixed modification was aminoethylation on cysteine (C, +43.042 Da). Variable modifications included oxidation of methionine and deamidation of asparagine and glutamine. Precursor and fragment error tolerances were set to 5 ppm and 20 ppm, respectively. Cysteines were aminoethylated to introduce additional tryptic cleavage sites, and tryptic digestion was defined to cleave at K, R, and C, with a maximum of three missed cleavages [34]. The rhEPO sequence was downloaded from UniprotKB. The monoisotopic *m/z* values of glycopeptide ions and their respective fragment ions identified by Glyco-Decipher were manually confirmed by searching the MS1 and MS2 spectra using Sciex OS software. For glycopeptide ions identified by the software, we examined the low mass range of the MS2 spectrum to confirm the presence of characteristic sugar fragments such as sialic acids. The most common source of misidentification was incorrect assignment of the monoisotopic mass due to its low intensity. For example, some glycans reported as containing three fucoses actually contained one fucose and one sialic acid, because the mass of two fucoses differs by only 1 Da from that of one sialic acid, and the monoisotopic peak was assigned to an isotopic signal. Retention time is also a useful criterion for distinguishing glycan compositions, as previous studies showed that glycans containing a sialic acid generally elute later than those containing two fucoses [45,49]. In addition, the relative intensities of sialic acid and other oxonium ions can further indicate the presence of multiple sialic acids. If the oxonium ion types, intensities, or the retention time were not consistent with the glycan composition given by the software, we interpreted MS2 spectra manually with a 1 Da decrease in monoisotopic mass. This was a rare event and was observed in glycopeptides with low (<100 cps) signal intensities, which did not appear to affect the glycan relative abundance analysis. The relative intensity of a specific glycopeptide was calculated as the peak area in its MS1 extracted ion chromatogram divided by the peak areas of all the glycopeptides determined in the sample by Glyco-Decipher [48]. The relative intensities of glycopeptides containing the same glycan were summed by the software to obtain the relative peak area of that glycan.

## 3. Results and discussion

Monitoring EPO concentrations is important during manufacturing, and this work uses peptides immobilized in membranes to capture EPO and enable its quantitation as well as glycan analysis. This section quantifies peptide immobilization in membranes, employs breakthrough curves to characterize EPO binding in peptide-modified membranes, and examines EPO capture and detection in 96-well plates containing peptide-functionalized membranes. Quantification of EPO in diluted CHO-cell supernatant demonstrates the relevance of the process for monitoring EPO concentration in fermentation broths. Finally, we identify EPO N-glycans by collecting eluates from peptide-functionalized membranes, performing in-membrane trypsin digestion and analyzing the resulting peptides using LC–MS/MS. We did not identify O-glycans because they are less abundant and less relevant to the protein’s function [50,51].

### 3.1. Immobilization of affinity peptides in membranes

To covalently immobilize peptides, we first deposited two PEI/PAA bilayers on the surface and in the pores of membranes to provide a high density of –COOH groups. We then activated these groups using EDC/NHS chemistry and allowed them to react with the amine groups on the N-terminal free lysine residues of EP13. This reaction not only immobilized the peptide but likely also crosslinked PEI and PAA to stabilize the polyelectrolyte layers [23]. We quantified peptide immobilization using the difference in the peptide concentrations before and after circulating a peptide solution through an activated membrane. This analysis employed determination of the peptide free amine groups using fluorescamine chemistry and gave an immobilization capacity of 15.2 ± 1.4 mg of EP13 per cm^3^ of membrane in large glass-fiber membranes.

When modifying membranes in 96-well plates, continuous circulation of derivatization solutions through the membranes as utilized above was not possible. Instead, we added solutions to individual wells containing membranes, incubated for several minutes, and drew the solutions through the membranes using vacuum. Repeating this step multiple times mimicked the immobilization process with continuous circulation of solution. However, replacing circulation with incubation may decrease the peptide-binding capacity [23,27]. Analysis of the solutions before and after passing through the membranes in the wells suggests an immobilization capacity of 3.1 ± 0.2 mg of EP13 per cm^3^ of membrane. The lower binding capacity observed in the 96-well plate format reflects both the use of incubation instead of circulation and a lower peptide concentration applied to reduce the ligand cost when modifying many wells. Circulation employed 3 mL of 1 mg/mL peptide for a 2-cm membrane (exposed membrane volume of 0.088 mL), whereas incubation employed a total of 1.2 mL of 0.2 mg/mL per well (exposed membrane volume of 0.033 mL).

### 3.2. Characterization of EPO capture in glass-fiber membranes modified with affinity peptides

**Fig. 2.**
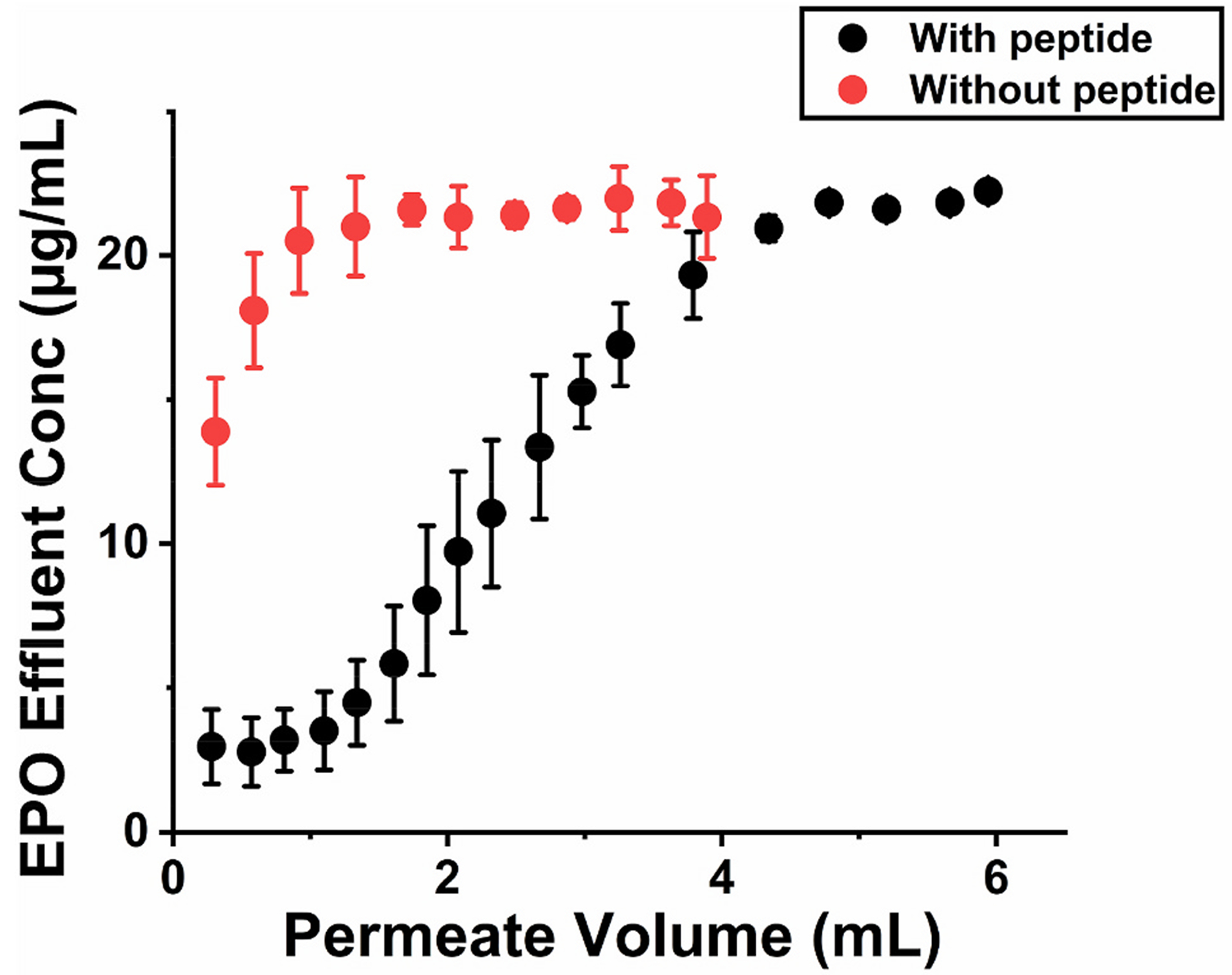
Breakthrough curves for EPO solutions passing through membranes modified with EP13 (black circles) or ethanolamine (red circles). In both cases, the EPO feed concentration was 25 μg/mL. Lower effluent concentrations indicate more EPO capture. Concentrations were determined using native fluorescence spectroscopy, and the flow rate was 1 mL/min. Error bars represent standard deviations from analyses with 3 different EP13-modified membrane and 2 different ethanolamine-modified membranes. Error bars in the X-direction are too small to see.

Studies of the capture and elution of EPO employed a 2-cm glass-fiber membrane to provide a high binding capacity. Fig. 2 shows EPO breakthrough curves obtained by passing 25 μg/mL EPO solutions through a membrane functionalized with EP13 and through a control membrane modified with ethanolamine rather than peptide. The plot shows the concentration of EPO exiting the membrane, and low effluent concentrations indicate high binding. The EP13-modified membrane captures approximately 85 % of the EPO in the first 1.5 mL of solution passed through the membrane, with most of the EPO captured from the first 2 mL. We calculated an approximate saturation binding capacity of 41 ± 3 μg of EPO by summing the products of each aliquot volume and the difference between permeate concentrations in the experimental and control groups. When divided by the membrane volume of 0.088 mL, the capacity is 0.47 ± 0.03 mg of EPO per mL of membrane. Reaction with ethanolamine rather than EP13 produced a membrane that captured only a small amount of EPO in the first 0.5 mL, and some of this is due to liquid dead volume in the system. Fig. S2 also shows the EP13 affinity membrane does not bind a significant fraction of host cell proteins from 1:800 diluted CHO-cell supernatant, highlighting the potential for selective EPO capture from this supernatant.

The membranes in the 96-well plates contain 2.6-fold less volume than those used in the breakthrough experiments in Fig. 2. Thus, if the binding properties of the two types of membranes were the same, membranes in the 96-well plates would bind 85 % of the EPO from around 0.5 mL of solution. However, the membranes in the well-plates have a lower affinity-peptide density.

### 3.3. Rapid quantitation of EPO in buffer using 96-well plates containing membranes

To quantify EPO, we captured this protein using membranes in 96-well plates and bound a secondary rabbit anti-human EPO antibody to the captured EPO. Finally, we conjugated a goat anti-rabbit IgG labeled with a DyLight650 fluorophore to the human antibody and quantified the fluorescence using a plate reader (Fig. 1).

**Fig. 3.**
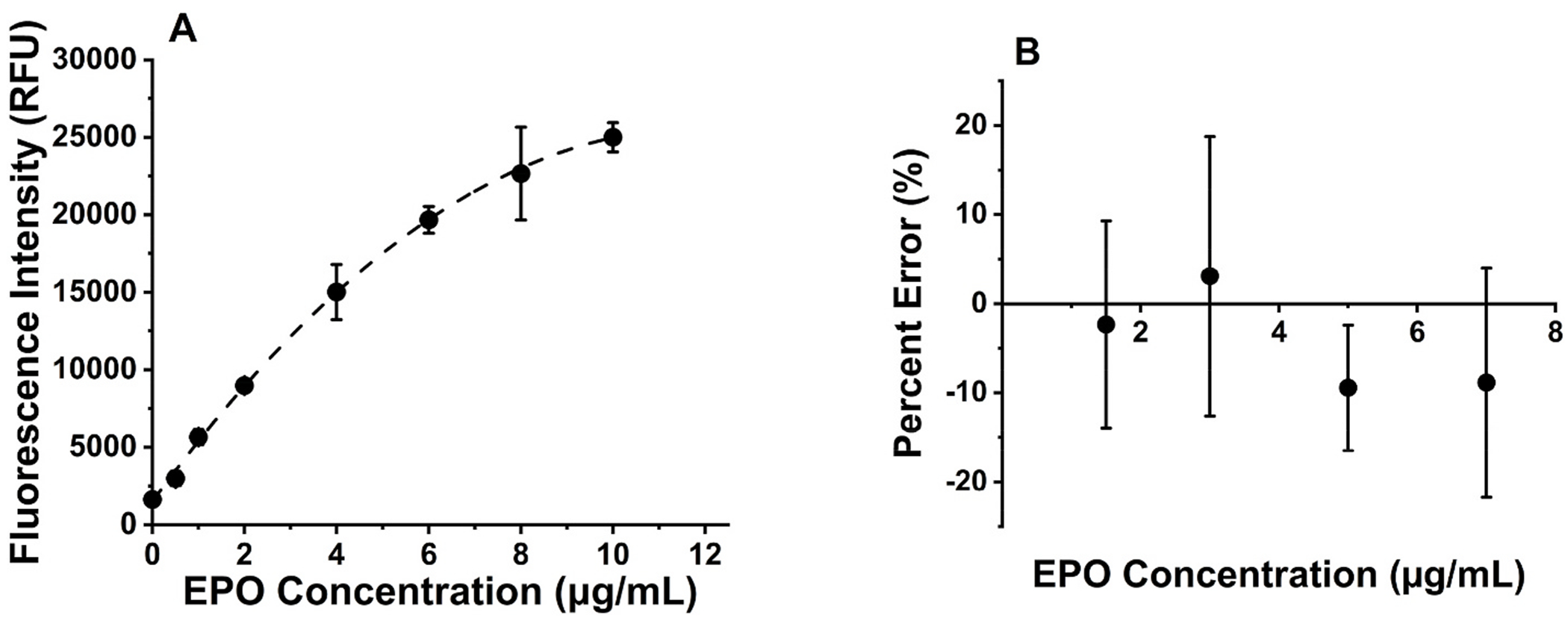
(A) The calibration curve for EPO analysis in 20 mM phosphate (pH 4) using EP13-modified membranes, capture of EPO, binding of a rabbit anti-human EPO antibody and an anti-rabbit antibody labeled with DyLight650. (B) Percent errors in EPO concentrations determined using the calibration curve in (A). The y-axis in (A) shows fluorescence emission at 675 nm and a quadratic regression fit to the data. Error bars in (A) and (B) show standard deviations from replicate analyses in 3 different wells in the same 96-well plate. In (A), the error bars are often smaller than the symbols.

Fig. 3A shows the calibration curve generated from a series of solutions with a range of EPO concentrations (0–10 μg/mL) in 20 mM phosphate (pH 4). Prior work suggests that this peptide is more selective for EPO binding at pH 4 than at pH 7.4 [46]. We fit the data with a quadratic regression. As expected, fluorescence intensity increased with higher EPO loading on the membranes. The curve was approximately linear at lower concentrations but plateaued at higher concentrations, suggesting that the binding begins to approach saturation at 10 μg/mL. Fig. S3A shows a linear calibration curve generated using EPO concentrations from 0 to 1000 ng/mL. The lower limit of quantitation (LLOQ), calculated as 10 times the standard deviation of the blank divided by the slope of the calibration curve, was 140 ng/ml. Practically, analysis with a coefficient of variation <20 % requires concentrations ≥200 ng/mL. Commercial ELISAs have lower LLOQs, but usually require hours to perform. Moreover, when monitoring fermentation broths ELISAs will require much greater dilution to achieve the detection range of the technique.

To evaluate reproducibility, we analyzed independent EPO samples in buffer B with known concentrations of 1.5, 3, 5, and 7 μg/mL. Fig. 3B shows the percent error in the concentrations calculated using the calibration curve in Fig. 3A. The low average percent error (2–10 %) and standard deviation (<16 %) demonstrate that the assay provides reasonable accuracy and precision, meeting the requirements for most EPO analyses [52,53]. As a single control experiment, we modified a membrane with ethanolamine, rather than EP13, and performed the steps to create a calibration curve. In this case, the fluorescence intensity change was only 10 % of the background on going from 0 to 10 μg/mL EPO (Fig. S3B).

### 3.4. Quantitation of EPO in CHO-cell supernatant

Next, we evaluated quantitation of EPO in diluted CHO-cell supernatant to simulate analysis from a fermentation broth. Recombinant EPO concentrations in industry production streams can reach several hundred micrograms per milliliter [15,54]. This would require at least a 1:10 dilution to achieve the range in the calibration curve in Fig. 3. Thus, we diluted the CHO-cell supernatant 1:10 (v/v) with 20 mM phosphate at pH 4, spiked this solution with a series of EPO concentrations, and analyzed these solutions using peptide-modified membranes.

**Fig. 4.**
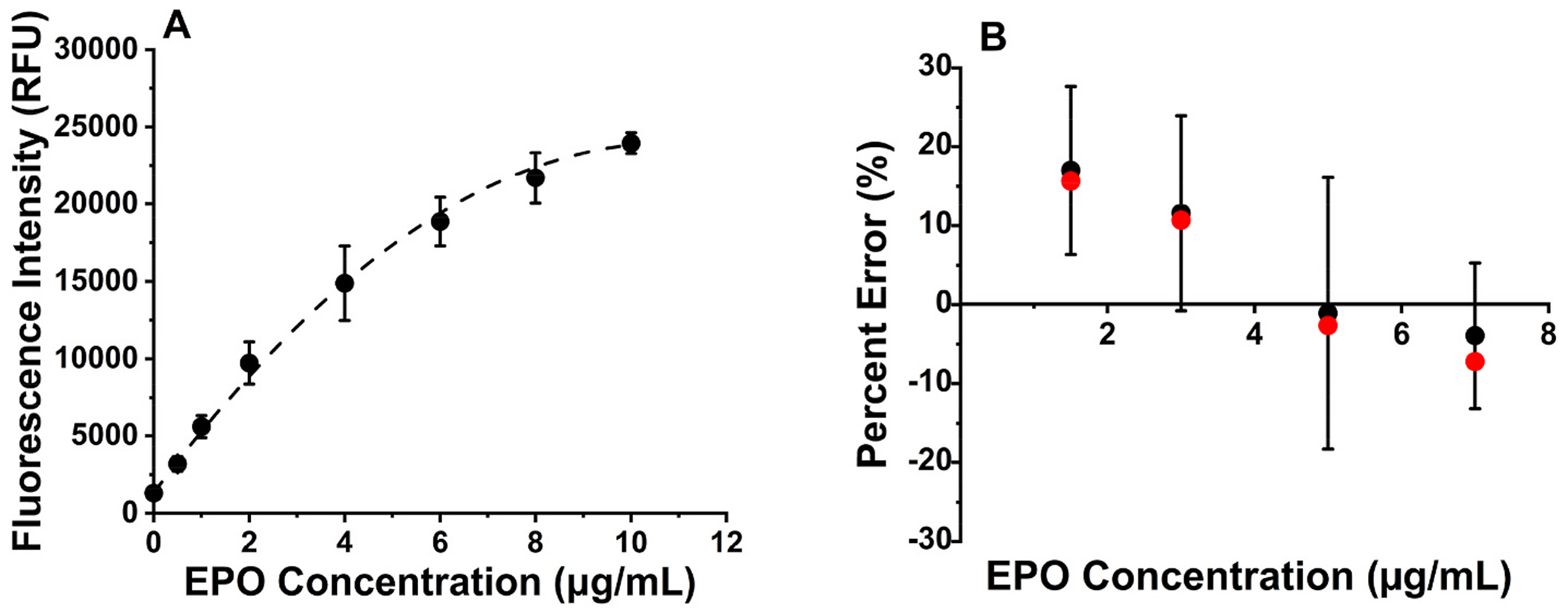
(A) Calibration curve for EPO analysis in 1:10 (v/v) diluted CHO-cell supernatant using EP13-modified membranes (B) Percent errors in the EPO concentrations determined using the calibration curve in (Fig. 4A) (black) and the calibration curve in (Fig. 3A) (red). The y-axis in (A) shows fluorescence emission at 675 nm and a quadratic regression fit to the data. Error bars represent standard deviations from four replicate analyses using the calibration curve in (Fig. 4A).

Fig. 4A shows the calibration curve for EPO spiked into 1:10 (v/v) diluted CHO-cell supernatant and analyzed using EP13-modified 96-well plates. The data points are not statistically different from those obtained in pH 4 phosphate, indicating that the affinity peptide-membrane system effectively captures EPO from CHO-cell supernatant. Using this calibration, we assessed accuracy by analyzing samples with known concentrations. To confirm the reproducibility, we analyzed independent EPO samples in 1:10 (v/v) diluted CHO-cell supernatant with known concentrations. Fig. 4B shows that the average percent errors from four independent replicates ranged from 1.1 % to 17 %, with standard deviations of 9.2 %–17.2 %, demonstrating both accuracy and precision. Fig. 4B also shows that the calibration curve in buffer B (Fig. 3A), gives similar concentrations when analyzing EPO in diluted CHO-cell supernatant, even though calibration curves were obtained on different days. Thus, it is not necessary to accurately match the matrix in standard and sample solutions. The results demonstrate that the assay should apply to EPO quantitation during production in CHO-cell fermentation broths.

### 3.5. Binding and elution of EPO from CHO-cell supernatant using 2-cm membranes

To examine capture and binding from mL volumes, we spiked 10 μg of EPO into 1 mL of 1:4 diluted CHO-cell supernatant (Buffer B) and circulated the solution through a 2-cm EP13-modified membrane for 2 min. After rinsing, we circulated 500 μL of 0.1 % SDS solution through the membrane for 2 min to elute EPO. We repeated this step with a second 500 μL of 0.1 % SDS to examine the extent of elution in the first aliquot.

Fig. S4 presents the SDS–PAGE analysis of the EPO binding and elution process. A clear EPO band appeared in the first elution, with no obvious host-cell proteins, indicating that the affinity membrane successfully isolated EPO from CHO-cell supernatant. The absence of visible protein bands in the second eluate aliquot suggests that the first elution was complete. Moreover, a substantial fraction of EPO was recovered, as the intensity of the EPO band from the first eluate is comparable to that for a 10 μg EPO standard loaded in Lane 9. The loading solution also contained 10 μg of EPO.

### 3.6. Identification of EPO N-glycans using LC-MS/MS

After SDS removal, the eluted EPO was denaturated with urea, reduced with DTT, digested, and desalted (see the SI for details). To introduce trypsin cleavage sites between the N-glycosylation sites at Asn51 and Asn65, we added 2-bromoethylamine hydrobromide to perform cysteine aminoethylation (Fig. S5) prior to digestion [34,55]. For comparison, we also examined standards containing 10 μg of EPO and performed all the steps in the process.

Glyco-decipher revealed many glycans and glycopeptides at the three N-glycosylation sites. We manually examined the MS1 and MS2 spectra to confirm the identities of all glycans. Fig. S6–S11 show some examples of extracted ion chromatograms (XIC), MS1 spectra, and MS2 spectra of selected glycopeptides. The monoisotopic *m/z* values in the MS1 spectra were consistent with their theoretical values, and the *m/z* values of fragment ions observed in the MS2 spectra matched expected fragments.

Table S1–S18 list the identified glycans from EPO standards dissolved in 0.08 % SDS and from EPO eluted in 0.1 % SDS after capture in an EP13-modified membrane. The abbreviations used to describe glycans are: Hex, hexose; HexNAc, N-acetylhexosamine; NeuAc, N-acetylneuraminic acid; and Fuc, fucose. Figs. S12 and S13 show the overlap among glycans identified in three replicate solutions for EPO standards or from three replicate membranes employed for capture and elution of EPO. After in-solution tryptic digestion, we identified at least 14 glycans at each glycosylation site in all three replicates of both standards and EPO captured in membranes.

**Fig. 5.**
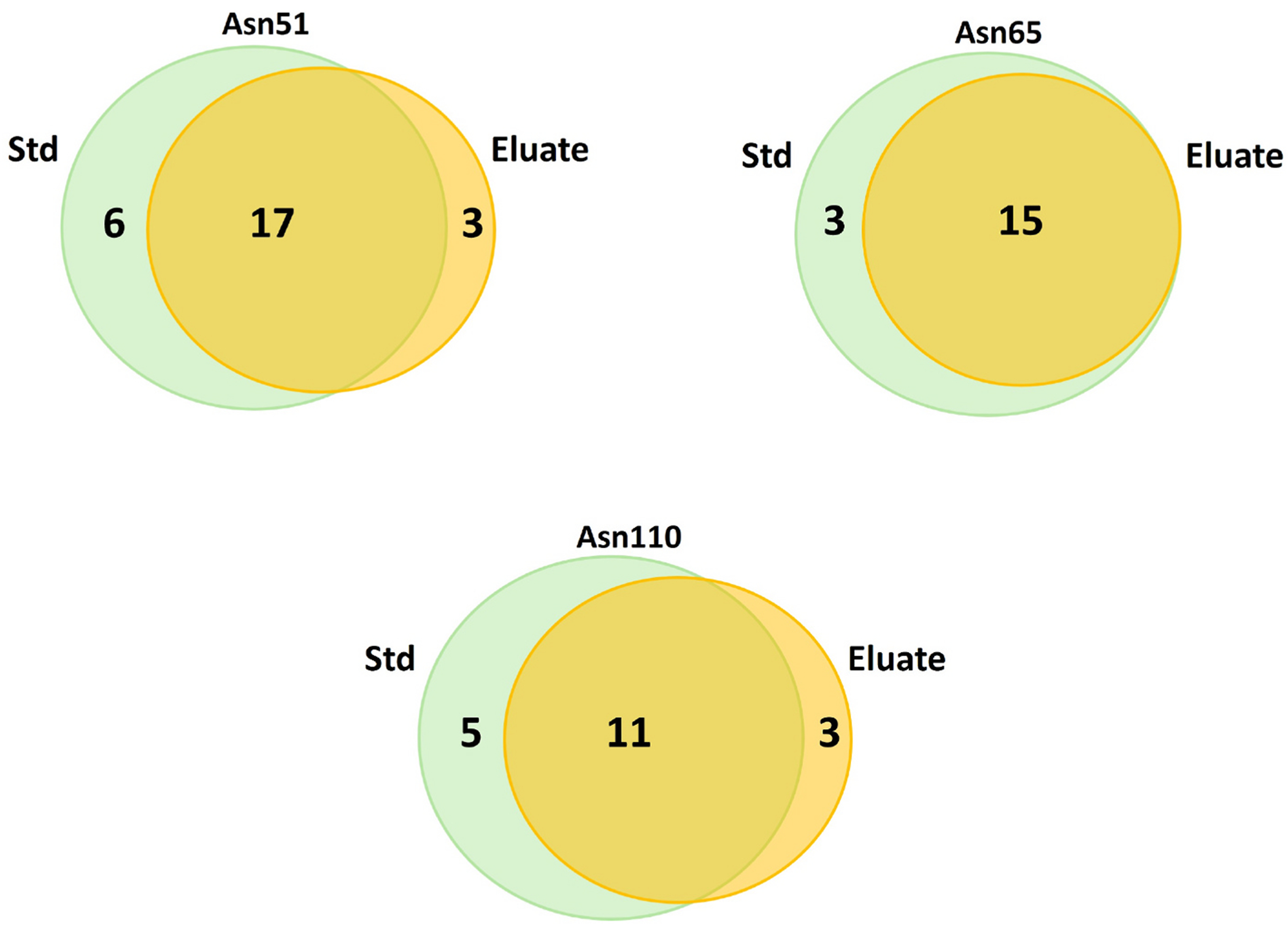
Comparison of the number of EPO N-glycans identified from a 10 μg EPO standard and from 10 μg of EPO spiked into CHO-cell supernatant and recovered using the affinity-peptide membrane system with elution in 0.1 % SDS. The figure displays only glycans detected in all three standard replicates or with all three membranes.

Fig. 5 provides the numbers of glycans identified in EPO standards and eluted EPO and shows the overlap. Comparing the standards and the eluted EPO, analyses revealed about 15 % more glycans at all three N-glycosylation sites when examining the standards. Nevertheless, analysis of the eluate also identified some glycans not found in all three of the standard replicates. Many of the peptides seen in all standard replicates but not all eluate replicates appeared in some of the eluate replicates and vice versa. These results demonstrate that capture and elution enable effective glycan analysis. Missing glycans in either standards or eluates are those with low signals.

Next, we investigated the relative intensities of glycopeptides from the EPO standards and the EPO recovered using the affinity-membrane system. Glyco-Decipher provided the relative peak areas of the glycopeptides in the MS1 spectra [48]. When a single glycan was detected in multiple peptides or at more than one N-glycosylation site, the areas were summed.

**Fig. 6.**
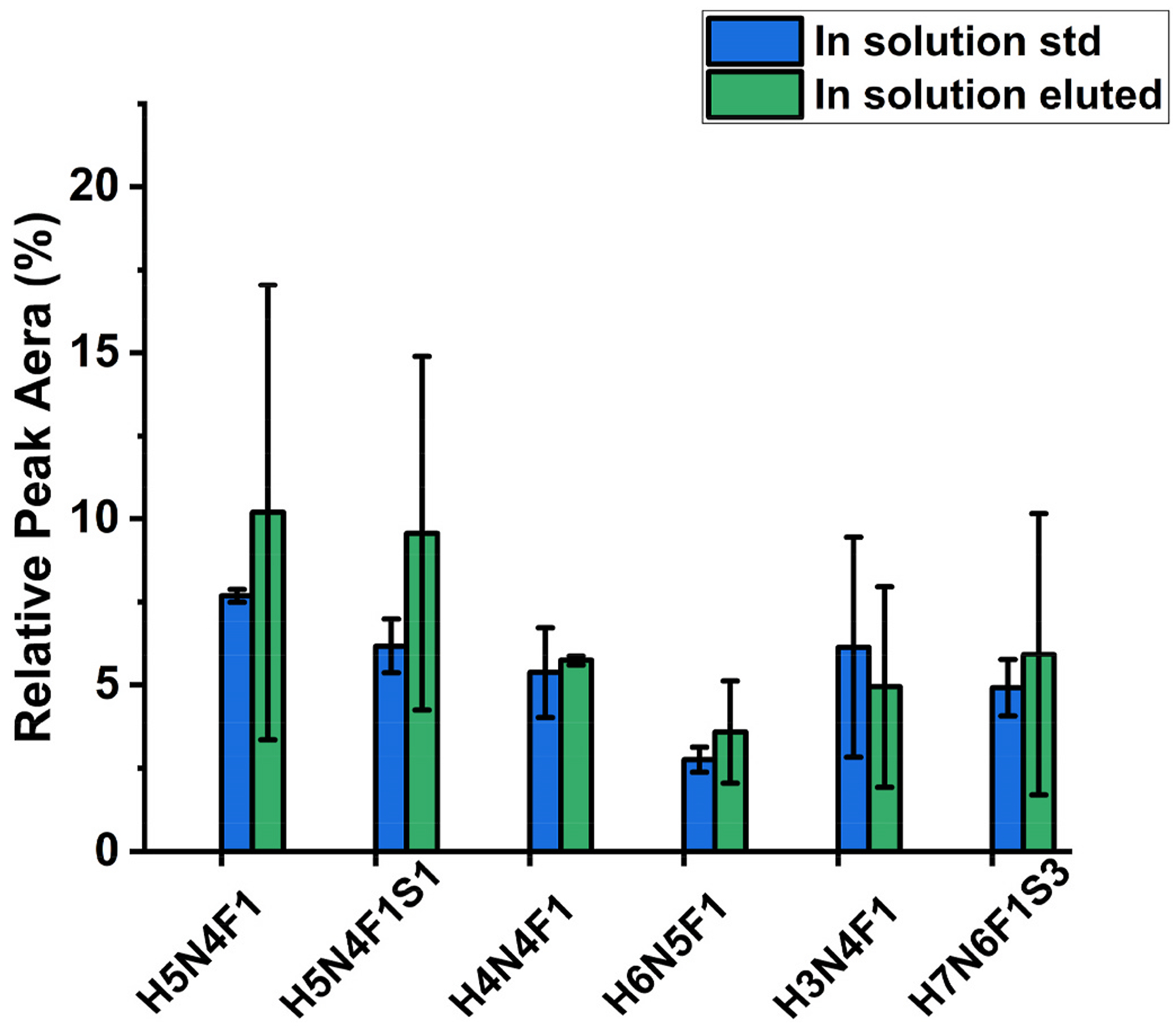
Relative peak areas of the six most intense glycans found at all three N-glycosylation sites in EPO standards and EPO recovered from CHO-cell supernatant and eluted from an affinity-membrane. The peak areas are those of glycopeptides after tryptic digestion. Error bars represent standard deviations from three replicates. Abbreviations: H, hexose; N, N-acetylhexosamine; F, fucose; S, N-acetylneuraminic acid. Proteins were digested in solution.

The EPO glycan relative peak areas did not change significantly after isolation from CHO-cell supernatant by the affinity-membrane system (Fig. 6). This result indicates that EP13-modified membranes do not introduce significant bias in the capture of EPO with different glycans. These findings suggest that the assay is effective for applications such as monitoring the continuity of EPO glycosylation during manufacturing in CHO cell cultures.

### 3.7. Rapid tryptic digestion of EPO using an enzyme-containing membrane

The previous section demonstrated rapid isolation of EPO from CHO-cell supernatant, and elution, digestion and LC-MS analysis provided reliable glycan identifications that are similar to those in EPO standards. However, the in-solution tryptic digestion, which requires several hours, increases the turnaround time for glycopeptide analysis via LC–MS/MS. We previously reported the use of enzyme-containing membranes to digest proteins within minutes, and this work employs such membrane for digestion of eluted proteins [45,56,57]. After elution, buffer exchange, denaturation, reduction, and alkylation, we passed the recovered EPO through a trypsin-containing membrane. We analyzed EPO standards after a similar in-membrane digestion. SDS–PAGE analysis showed no detectable residual undigested EPO after passing the protein through a trypsin-containing membrane (see Fig. S14).

### 3.8. Comparison of glycan identification after in-membrane tryptic digestion and overnight in-solution digestion

To evaluate whether the trypsin-modified membranes can serve as an alternative to conventional in-solution digestion for glycan analysis, we compared site-specific glycan identifications from EPO standards processed by in-solution and in-membrane digestion. Tables S19–S36 list the identified glycopeptides from in-membrane–digested EPO. Compared with overnight in-solution digestion, the rapid in-membrane digestion was more likely to result in missed cleavages. At Asn51, most glycopeptides were identified as EAENITTGC in the in-solution–digested EPO, whereas in the in-membrane digestions most glycopeptides appeared as EAENITTGCAEHC.

**Fig. 7.**
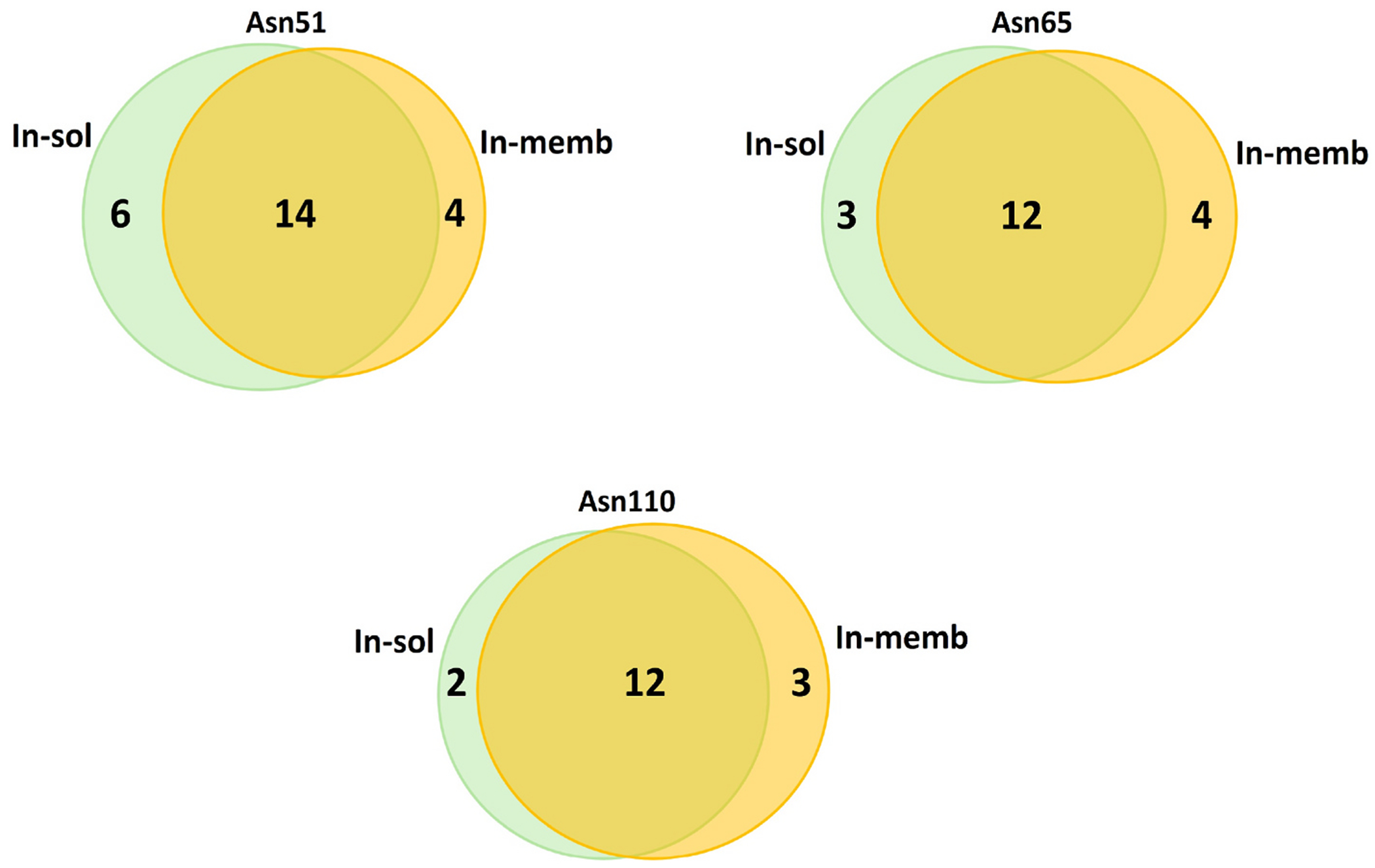
Comparison of EPO N-glycans identified from 10 μg of EPO spiked into CHO-cell supernatant and recovered using an affinity membrane. Analysis occurred after either in-solution tryptic digestion or in-membrane digestion. Each group includes three replicates, and only glycans detected in all replicates are shown.

Figs. S15 and S16 show overlaps of glycan identifications from EPO standards and recovered EPO after three replicate in-membrane tryptic digestions. Identifications are highly reproducible. Fig. 7 shows the overlap of site-specific glycans identified after in-solution tryptic digestion or in-membrane tryptic digestion using EPO recovered from CHO-cell supernatant (spiked with 10 μg of EPO). The numbers of peptides reproducibly identified after the two digestion methods are approximately the same. A similar result occurred with EPO standards (Fig. S17).

**Fig. 8.**
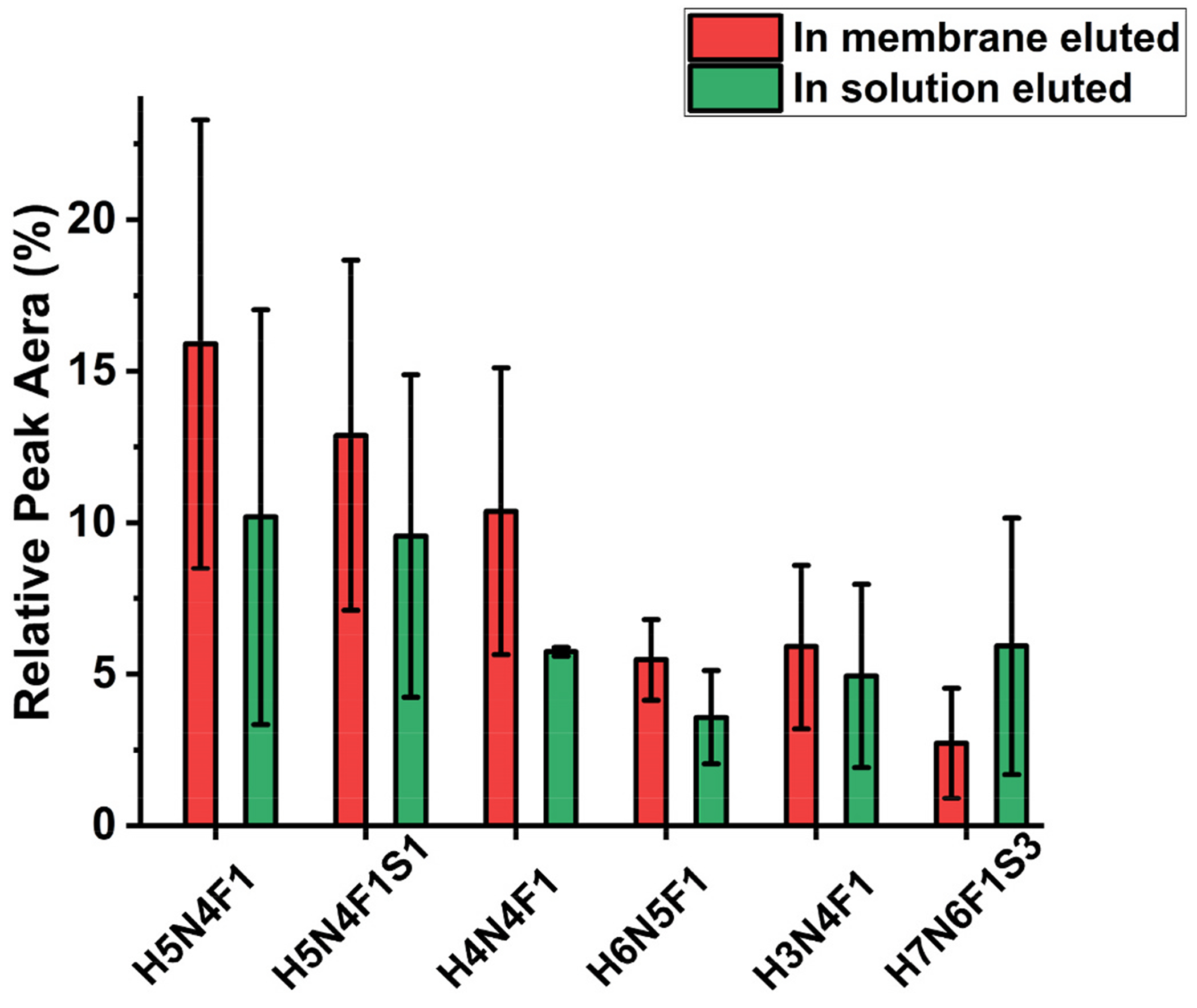
Relative peak areas of the six most intense N-glycans found at all three N-glycosylation sites from in-membrane and in-solution digested EPO recovered from CHO-cell supernatant and eluted from an affinity-membrane. Error bars represent standard deviations from three replicates. Abbreviations: H, hexose; N, N-acetylhexosamine; F, fucose; S, N-acetylneuraminic acid.

We also examined the relative peak areas of glycopeptides identified from in-membrane–digested EPO eluted from an affinity membrane. Fig. 8 shows the relative peak areas of the six most intense glycans (as determined from their glycopeptides) from EPO recovered from CHO-cell supernatant. Analyses occurred after in-solution and in-membrane tryptic digestion. Results from a Student’s t-test indicated no statistically significant differences in glycan peak areas (95 % confidence) between in-solution and in-membrane digestion. In-membrane digestion produced longer glycopeptides, and the glycopeptide length can alter ionization efficiency [45]. Nevertheless, the overall glycan relative peak did not change significantly. Fig. S18 shows that the relative glycan peak areas (as determined from glycopeptides) in EPO standards did not change significantly (95 % confidence) when using in-membrane digestions compared to in-solution digestion.

Overall, we identified comparable numbers and compositions of glycans after in-solution and in-membrane tryptic digestion of EPO, while the in-membrane digestion required only 6 min.

To evaluate the necessity of affinity enrichment for glycan identification of EPO, we directly digested CHO-cell supernatant spiked with EPO. Specifically, 10 μg of EPO was spiked into 1 mL of 1:9 diluted CHO-cell supernatant, and to prevent overloading, the mixture was further diluted to ~0.4 mg/mL total protein with deionized water. Without affinity-membrane enrichment or elution, the mixture was filtered through a 0.22 μm PES syringe filter, followed by buffer exchange, denaturation, reduction, alkylation, and in-solution tryptic digestion following the same protocol described in this paper. However, no EPO glycopeptides were identified in the software-processed results, indicating that enrichment is necessary.

## 4. Conclusions

This study demonstrates the versatility of affinity membranes in EPO isolation and quantification. After EPO capture in membranes in 96-well plates, binding of fluorescently labeled secondary antibodies enabled quantification of EPO in CHO-cell supernatant in less than 10 min. We also established a workflow combining rapid EPO enrichment using an affinity membrane with rapid digestion using a trypsin-containing membrane. In-membrane digestions yielded EPO glycan identifications comparable to those obtained by in-solution digestion, but in-membrane digestion occurs in minutes. EPO glycans identified before and after affinity capture were essentially the same. This work demonstrates the versatility of affinity and tryptic membranes for both rapid quantitation and glycosylation analysis during EPO production.

## Supplementary Material

1

## Data Availability

Excel figures and mass spectra are available in Mendeley Data at DOI: 10.17632/wwb6bsw36g.1.

## Appendix A. Supplementary data

Supplementary data to this article can be found online at https://doi.org/10.1016/j.talanta.2026.129396.
