# Supplementary material for: Rapid quantitation of erythropoietin and identification of its glycans using membranes for capture and digestion
Source: Talanta. Author manuscript; Available in PMC 2026 Jul 1. (PMC13321599; doi:10.1016/j.talanta.2026.129396)
Supplement: 1 [file NIHMS2189627-supplement-1.pdf]

Supplementary material to:

Rapid Quantitation of Erythropoietin and Identification of Its Glycans using Membranes for Capture and Digestion

Yuhang Chen[a], Bill Boggess[a], Junyan Yang[b], Nicholas E. Manicke[c], Merlin L. Bruening[a,b*]

[a]Department of Chemistry and Biochemistry, University of Notre Dame, Notre Dame, IN, 46556, United States

[b]Department of Chemical and Biomolecular Engineering, University of Notre Dame, Notre Dame, IN, 46556, United States

[c]Department of Chemistry and Chemical Biology, Indiana University Indianapolis, Indianapolis, IN 46202, United States

Email: mbruenin@nd.edu

*Corresponding author

Contents

S1. Modification of 2-cm Membranes with Peptides or Trypsin 1

S2. Digestion of EPO using a Trypsin-Modified Membrane 1

Figure S1. Schematic workflow of EPO denaturation, reduction, alkylation, and digestion 3

Figure S2. Breakthrough curves for CHO cell supernatant HCP in pH 4, 20 mM phosphate passing through an EP13-modified membrane. 3

Figure S3. (A) Low-concentration calibration curve for EPO analysis in Buffer B obtained from a 96-well plate modified with EP13 . (B) EPO “calibration curve” obtained from a 96-well plate containing membranes without EP13. 4

Figure S4. SDS–PAGE analysis of EPO in 1:4 CHO cell supernatant before and after flowing through a membrane, and in the eluate from a peptide-modified membrane. 4

Figure S5. Scheme of aminoethylation to introduce additional tryptic cleavage sites between Asn51 and Asn65 in EPO. 5

Figures S6-S11. Extracted ion chromatograms, and MS1 and MS2 spectra of selected glycopeptides 6

Tables S1-S18. EPO N-glycans identified from in-solution trypsin digested samples 15

Figure S12. Overlap of glycans identified from EPO standards after three replicate in-solution tryptic digestions. 33

Figure S13. Overlap of glycans identified from recovered EPO after three replicate in-solution tryptic digestions. 33

Tables S19-S36. EPO N-glycans identified from in-membrane tryptic digested samples 34

Figure S14. SDS–PAGE analysis of EPO before and after in-membrane tryptic digestion. 52

Figure S15. Overlap of glycans identified from EPO standards after three replicate in-membrane tryptic digestions. 52

Figure S16. Overlap of glycans identified from EPO recovered using three different affinity membranes prior to three independent in-membrane tryptic digestions. 53

Figure S17. Comparison of EPO glycans identified from 10 μg of EPO standard digested either in solution or in a membrane. 53

Figure S18. Relative peak areas of the six most intense glycans found at all three N-glycosylation sites for in-membrane and in-solution tryptic digested EPO standards. 54

Reference: 55

S1. Modification of 2-cm Membranes with Peptides or Trypsin

A 25 mm-diameter glass-fiber membrane was functionalized with polyelectrolytes using a peristaltic pump to circulate solutions through the membranes at a flow rate of 1 mL/min. Unless specified otherwise, all flow rates through 2-cm membranes were 1 mL/min. The membrane was first treated with UV/ozone (Jelight, Model 18) for 10 min and then placed in customized membrane holder. A rubber o-ring was placed in the membrane holder to fix the membrane and reduce the exposed aera to a 2 cm-diameter circle. A 10-mL solution of 2 mg/mL branched PEI at pH 3 was circulated through the membrane for 15 minutes followed by a 10 mL water rinse. Next, 5 mL of 1.1 mg/mL PAA in 0.5 M NaCl at pH 3 was circulated through the membrane, and the membrane was again rinsed with 10 mL of water. Solution pH values were adjusted with HCl or NaOH solutions. A second PEI/PAA bilayer was then deposited using the same procedure. Subsequently, 5 mL of an aqueous solution containing 0.1 M EDC and 0.1 M NHS was circulated through the membrane for 1 hour. The membrane was then rinsed with 10 mL of DI water, followed by circulation of 3 mL of 1 mg/mL EP13 in Buffer A through the membrane for 1 h. After circulation, the solution was collected for concentration determination. Finally, 10 mL of Buffer A was passed through the membrane to remove unbound peptides. The concentration of peptides in solution was determined using fluorescamine chemistry. Briefly, fluorescamine was dissolved in acetonitrile at a concentration of 2 mg/mL, and a calibration curve was established by mixing fluorescamine with peptide standards (20-100 μg/mL) in phosphate buffer to give a final fluorescamine concentration 0.1 mg/mL. After mixing, samples were incubated in the dark for 10 min and then transferred to a 96-well plate. Fluorescence was measured with excitation at 390 nm and emission at 470 nm. The concentrations of peptides in loading, effluent, and rinsing solutions were determined to calculate the amount of peptide in the membrane by subtraction.

To produce a trypsin-containing membrane, a 25 mm-diameter *nylon* membrane was cleaned for 10 minutes using a UV/ozone cleaner, inserted into a membrane holder (exposed diameter of 2 cm) and rinsed with 10 mL of deionized water. Subsequently, 10 mL of 3.7 mg/mL PSS (500 mM NaCl, pH 2.3) was circulated through the membrane for 20 minutes, followed by a rinse with 30 mL of deionized water. The flow rate was 1 mL/min. Next, 1 mL of 1 mg/mL TPCK-treated trypsin in 2.7 mM HCl was circulated through the membrane for 30 minutes, followed by a 30 mL rinse with 1 mM HCl. The rinsed membrane was then dried with N_2 and stored in a refrigerator. Trypsin immobilization was confirmed by measuring the intrinsic tryptophan fluorescence of the trypsin feed and flow-through solutions. This follows a literature procedure [1].

S2. Digestion of EPO using a Trypsin-Modified Membrane

Figure S1 shows a scheme of EPO denaturation, reduction, alkylation, and digestion. For EPO reduction, aminoethylation and digestion, we followed adapted literature protocols [1,2]. The

eluted EPO was added to a 10 kDa molecular weight cutoff filter and centrifuged at 14000 rpm for 15 min to concentrate the protein to approximately 30 µL. For analysis of an EPO standard, 100 µL of 0.1 mg/mL EPO in Buffer A was mixed with 400 µL of 0.1% SDS and added to a 10K filter for concentration in the same way. The concentrated EPO was diluted with 400 µL of 50 mM ammonium bicarbonate and centrifuged to a final volume of 30 µL using a 10 kDa filter. This buffer-exchanged EPO was subsequently added to 50 µL of 50 mM ammonium bicarbonate containing 38.4 mg urea to give a final urea concentration of 8 M. Afterward, 3 µL of 16 mg/mL DTT in 8 M urea was added, and the solution was incubated at 37 °C for 1 hour in the dark. Subsequently, 11 µL of 36 mg/mL 2-bromoethylamine in 8 M urea was added, and the solution was incubated for an additional hour at 60 °C in the dark. The alkylated EPO was added to 400 µL of ammonium bicarbonate and reduced in volume to 30 µL using a 10K filter; this step was repeated to give further desalting. For in-solution trypsin digestion, 0.2 µg of sequencing-grade modified trypsin in 2 µL of 50 mM ammonium bicarbonate was added to the recovered EPO, and the solution was incubated overnight at 37 °C. For in-membrane trypsin digestion, the retentate was resuspended in approximately 70 µL of ammonium bicarbonate to reach a final volume of ~100 µL. The 100 µL of alkylated EPO was passed through the trypsin membrane (~0.02 cm^2 exposed area) using a syringe pump at a flow rate of 1 mL/h. The digested samples were dried using a SpeedVac vacuum concentrator and reconstituted in 30 µL of a solution containing 4% (v/v) acetonitrile and 0.1% formic acid in DI water.

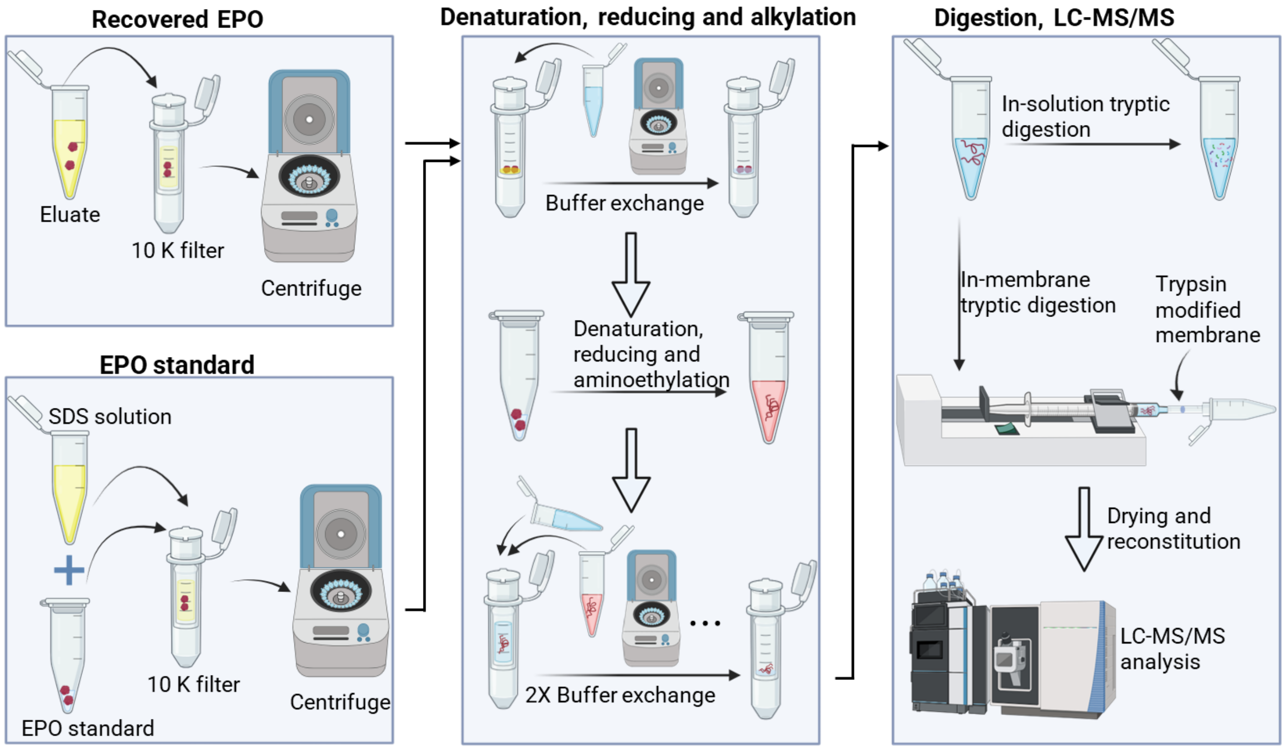

Figure S1. Schematic workflow of EPO denaturation, reduction, alkylation, and digestion.

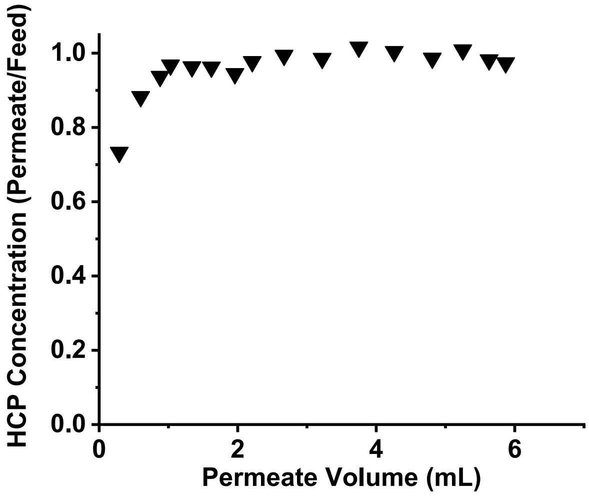

Figure S2. Breakthrough curves for CHO cell supernatant HCP in pH 4, 20 mM phosphate passing through an EP13-modified membrane. The host-cell protein (HCP) concentration was roughly 25 µg/mL as determined using protein precipitation of a stock solution. HCP concentrations relative to the feed were determined using native fluorescence spectroscopy (excitation 280 nm, emission 330 nm), and the flow rate was 1 mL/min.

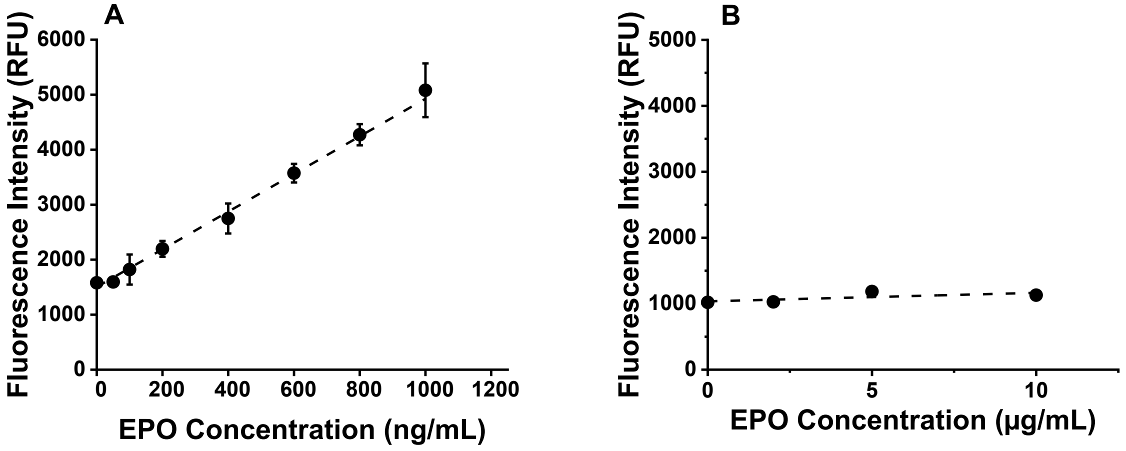

Figure S3. (A) Low-concentration calibration curve for EPO analysis in Buffer B obtained from a 96-well plate modified with EP13 . (B) EPO “calibration curve” obtained from a 96-well plate containing membranes without EP13. A series of EPO solutions at different concentrations were passed through the membranes, followed by solutions containing an anti-EPO antibody and a fluorescently labeled secondary antibody, and fluorescence analysis.

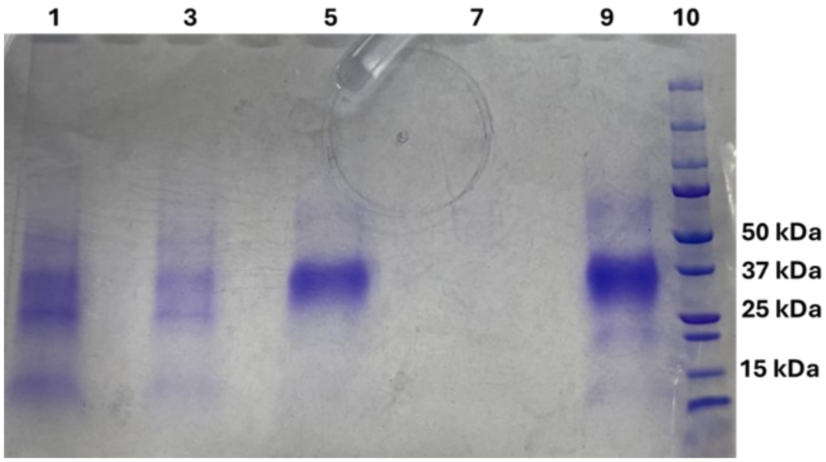

Figure S4. SDS–PAGE analysis of EPO in 1:4 CHO cell supernatant before and after flowing through a membrane, and in the eluate from a peptide-modified membrane. Lane 1: Protein from 20 µL of the feed solution spiked with 10 µg/mL EPO; Lane 3: Protein from 20 µL of same flow-through solution; Lane 5: Protein from the first 500 µL SDS eluate; Lane 7: Protein from the second 500 µL SDS eluate; Lane 9: 10 µg EPO standard; Lane 10: molecular weight markers.

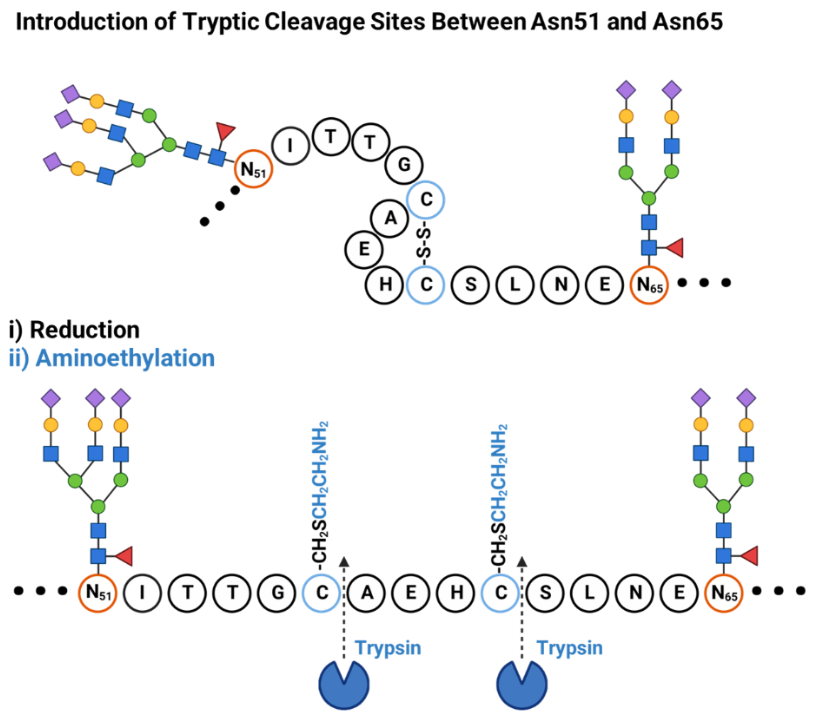

Figure S5. Scheme of aminoethylation to introduce additional tryptic cleavage sites between Asn51 and Asn65 in EPO.

Figures S6-S11. Extracted ion chromatograms, and MS1 and MS2 spectra of selected glycopeptides

(A)

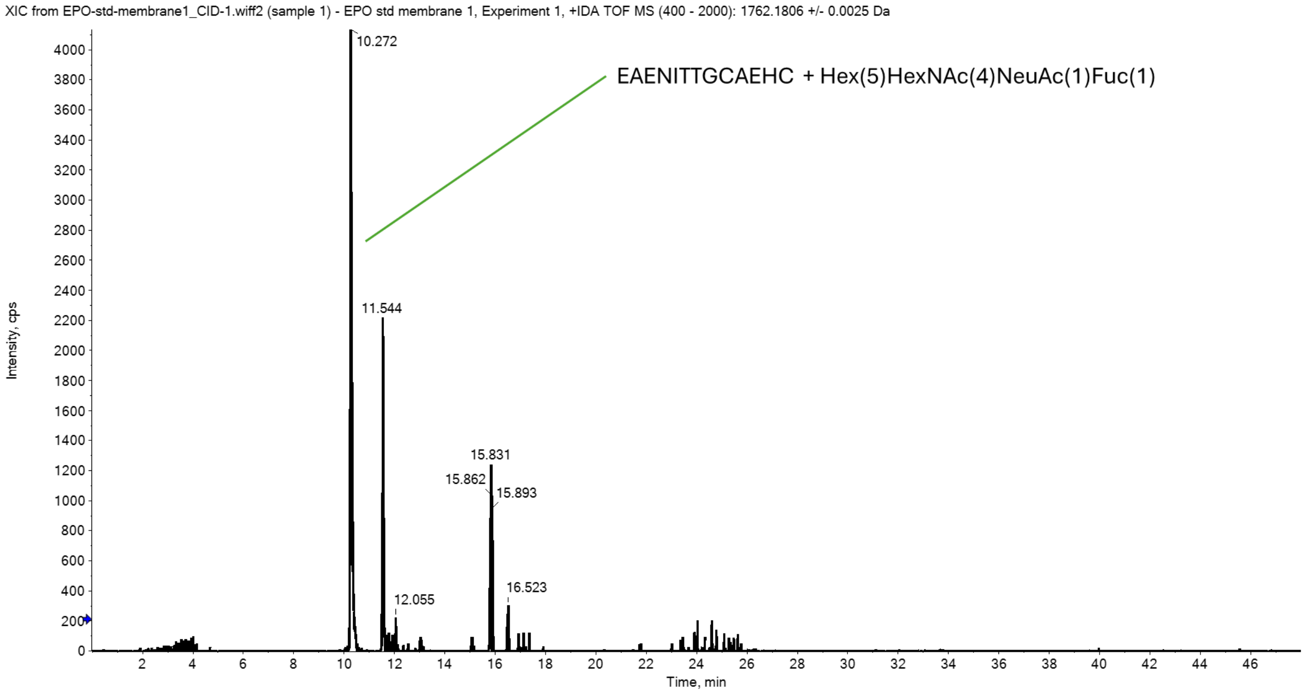

(B)

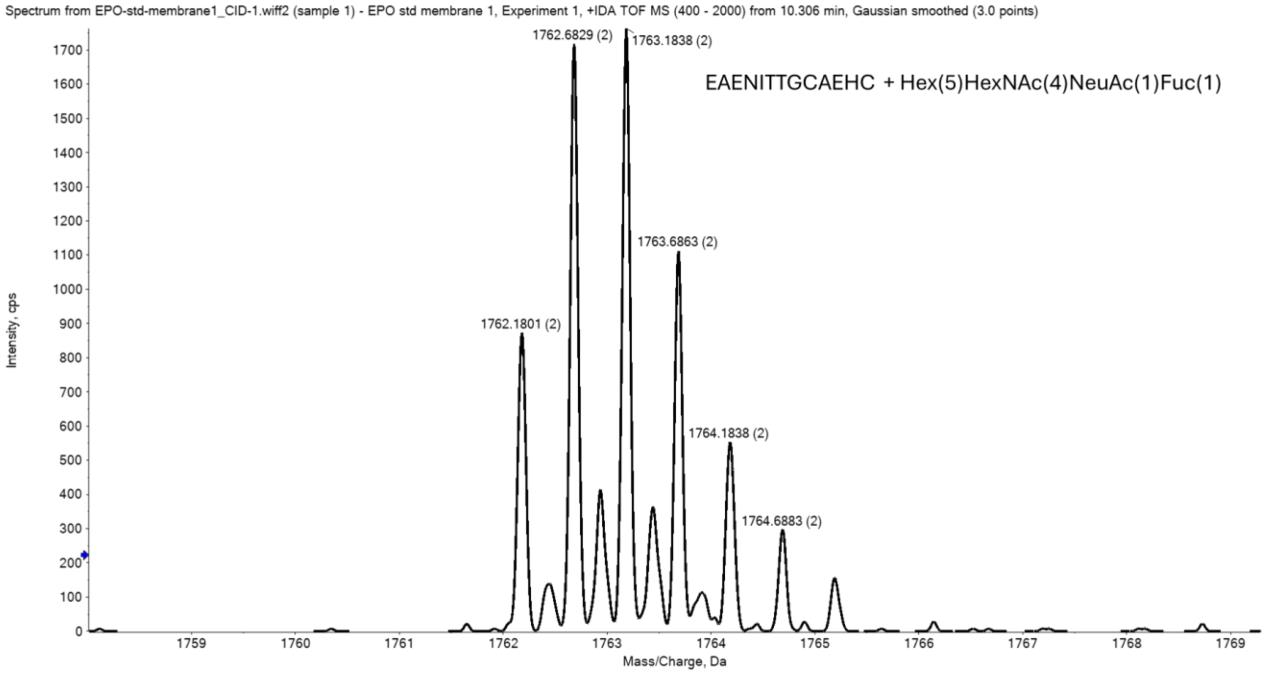

(C)

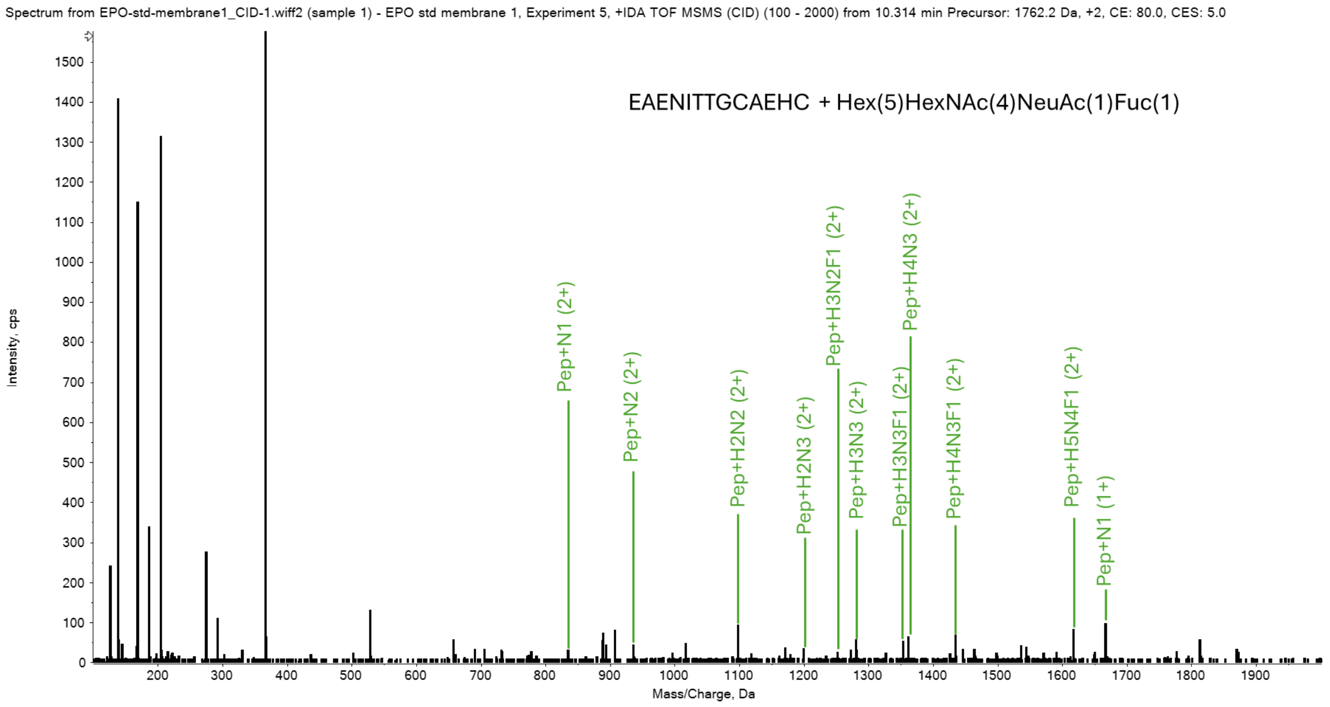

Figure S6. Extracted ion chromatogram (A) and MS1 (B) and MS2 (C) spectra of the EPO glycopeptide EAENITTGCAEHC+Hex(5)HexNAc(4)NeuAc(1)Fuc(1) with +2 charge.

(A)

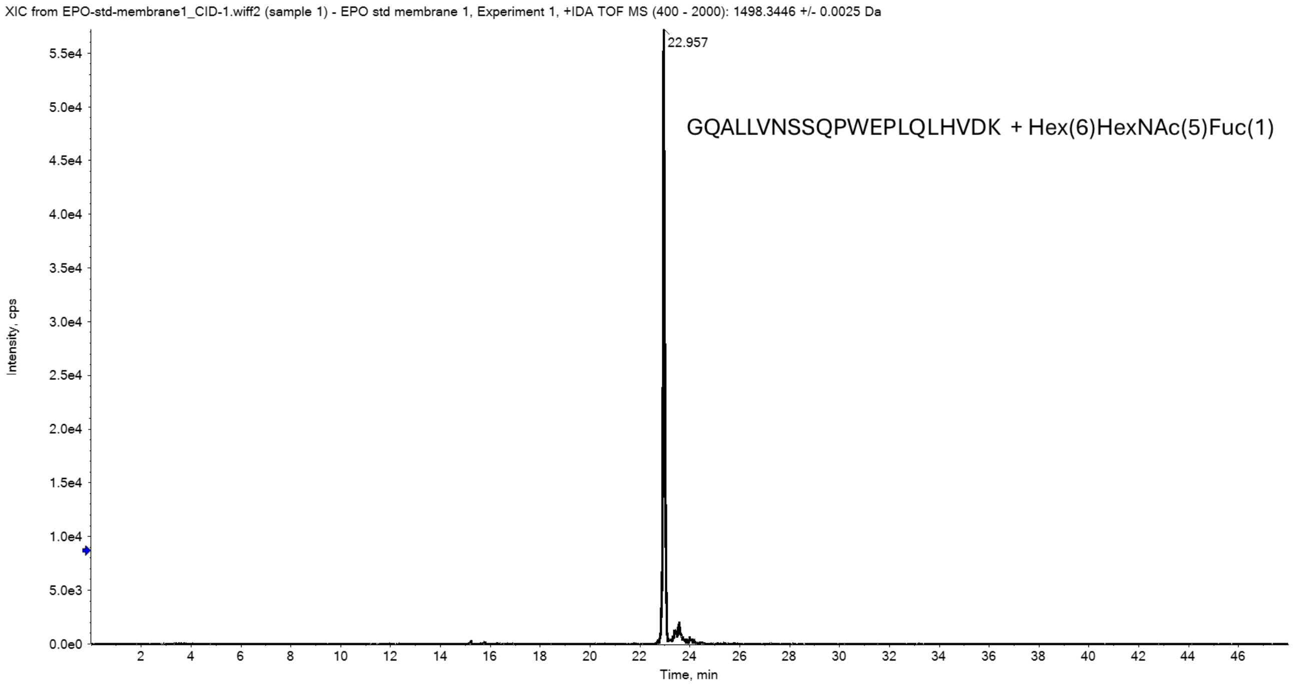

(B)

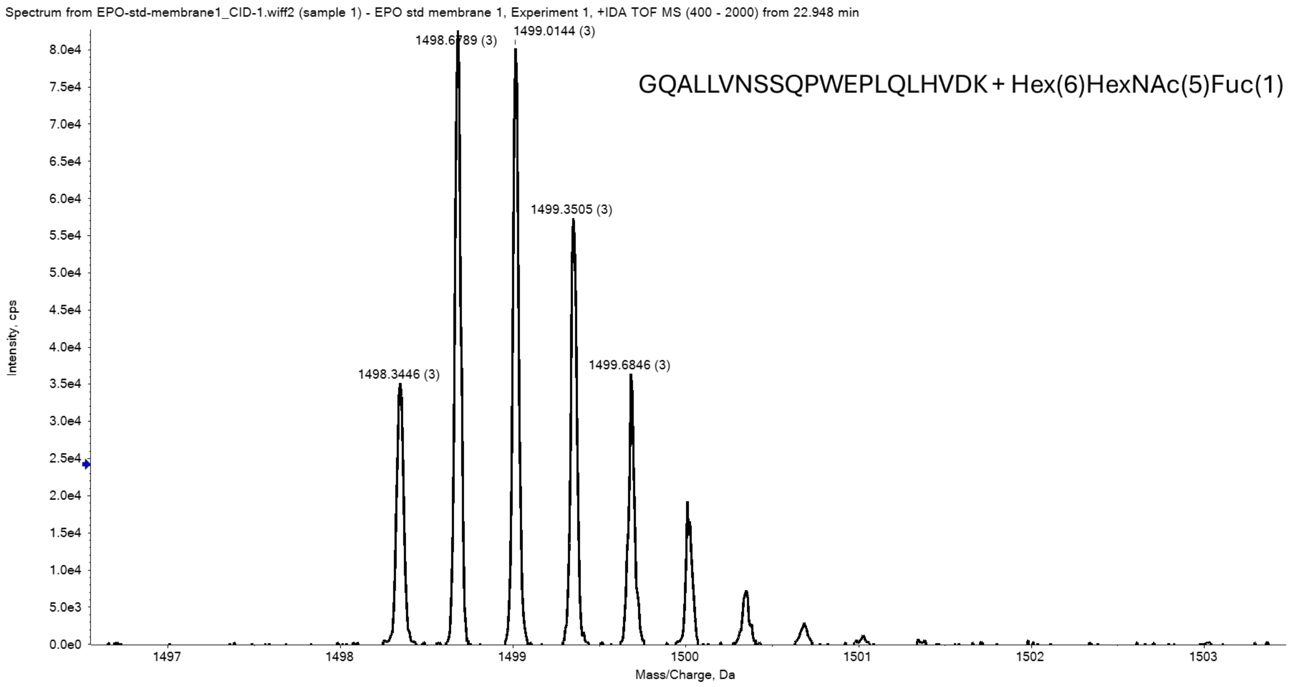

(C)

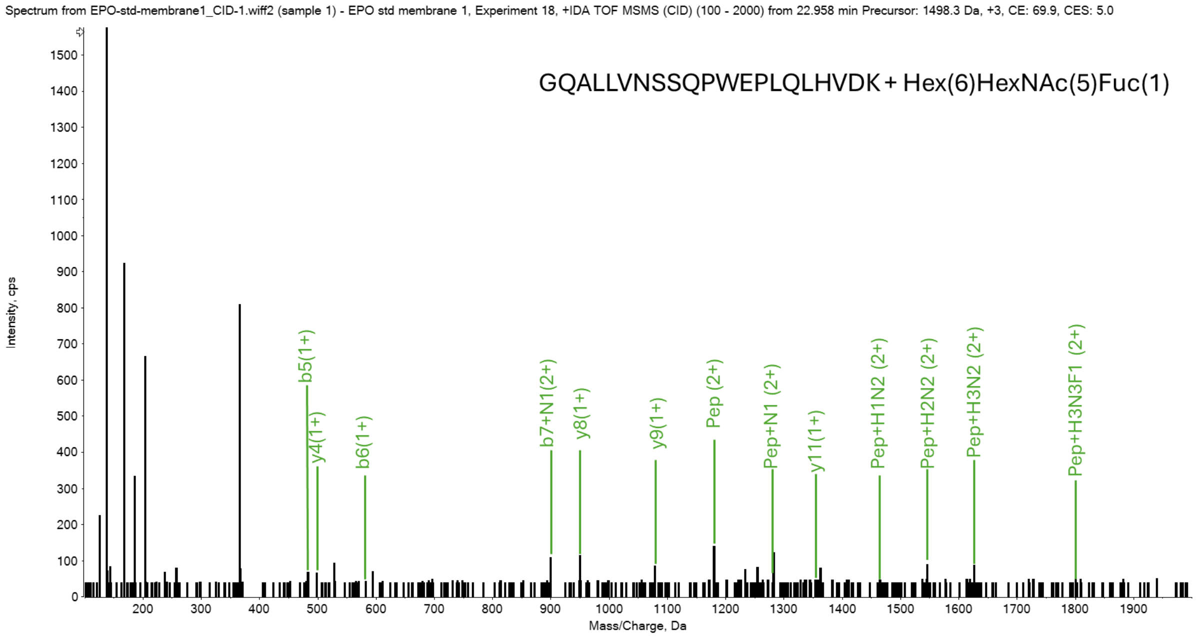

Figure S7. Extracted ion chromatogram (A) and MS1 (B) and MS2 (C) spectra of the EPO glycopeptide GQALLVNSSQPWEPLQLHVDK+Hex(6)HexNAc(5)Fuc(1) with +3 charge.

(A)

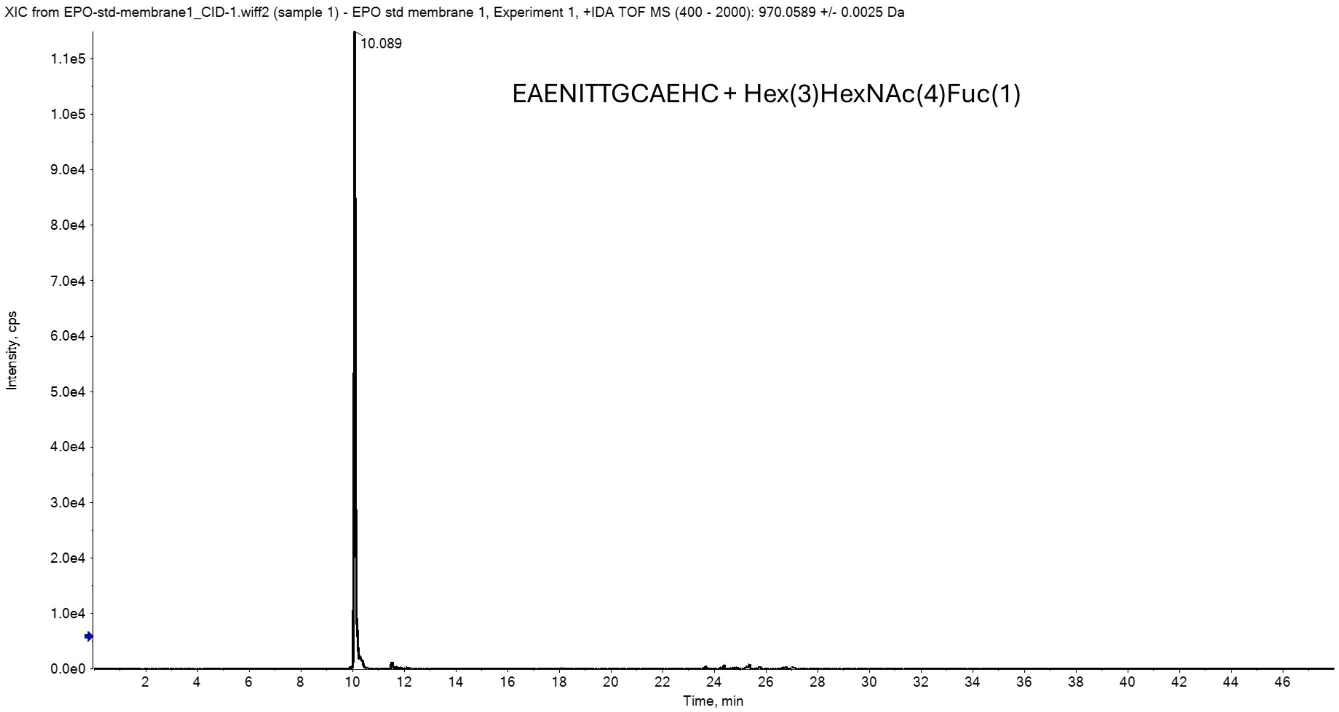
XIC from EPO-std-membrane1_CID-1.wiff2 (sample 1) - EPO std membrane 1, Experiment 1, +IDA TOF MS (400 - 2000): 970.0589 +/- 0.0025 Da
10.089
EAENITTGCAEHC + Hex(3)HexNAc(4)Fuc(1)
Intensity, cps
Time, min

(B)

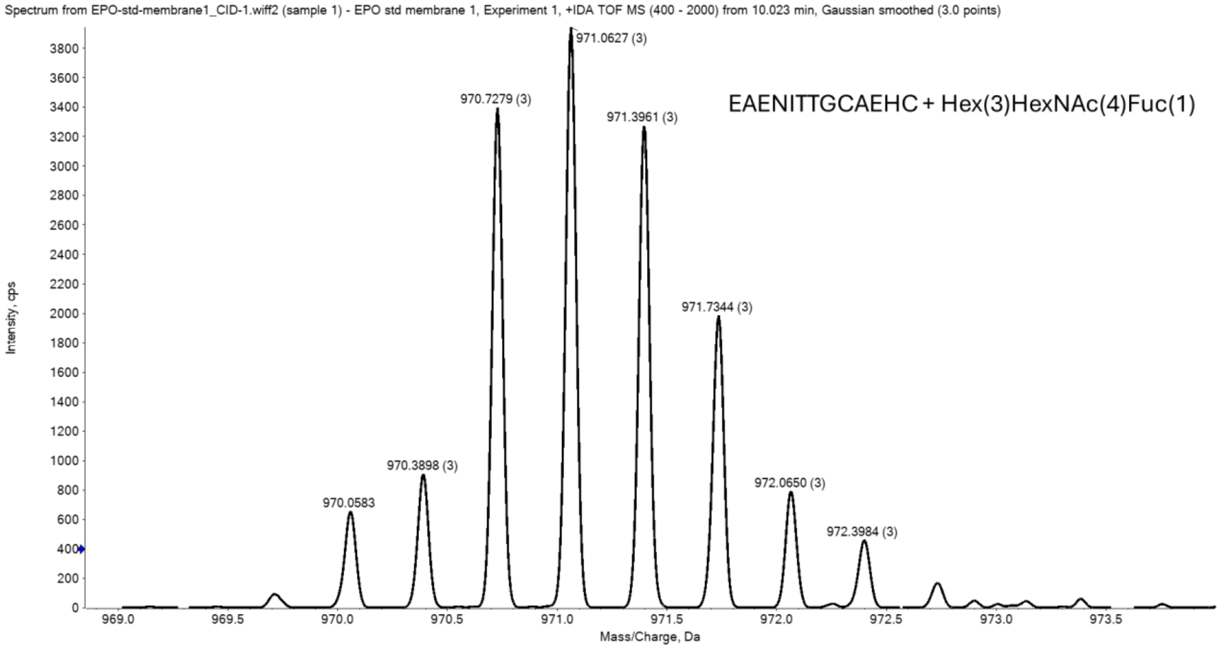
Spectrum from EPO-std-membrane1_CID-1.wiff2 (sample 1) - EPO std membrane 1, Experiment 1, +IDA TOF MS (400 - 2000) from 10.023 min, Gaussian smoothed (3.0 points)
971.0627 (3)
970.7279 (3)
971.3961 (3)
EAENITTGCAEHC + Hex(3)HexNAc(4)Fuc(1)
971.7344 (3)
970.3898 (3)
970.0583
972.0650 (3)
972.3984 (3)
Intensity, cps
Mass/Charge, Da

(C)

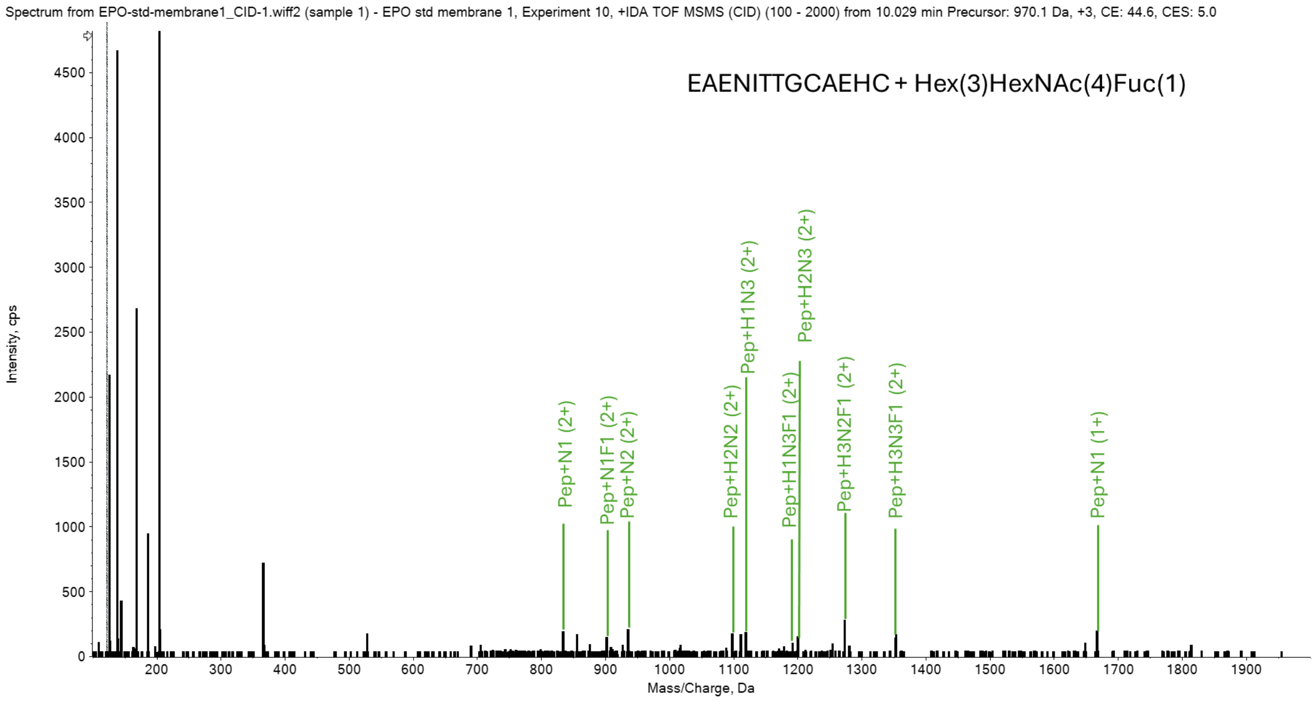

Figure S8. Extracted ion chromatogram (A) and MS1 (B) and MS2 (C) spectra of the EPO glycopeptide EAENITTGCAEHC+Hex(3)HexNAc(4)Fuc(1) with +3 charge.

(A)

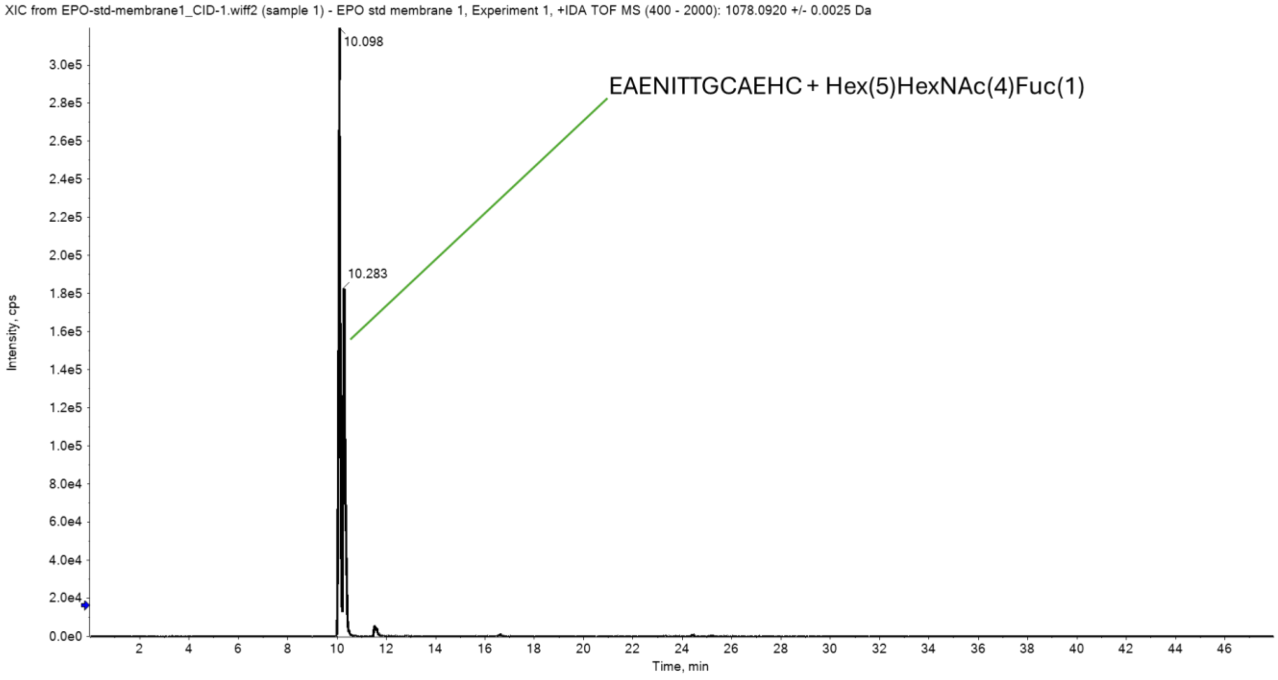

(B)

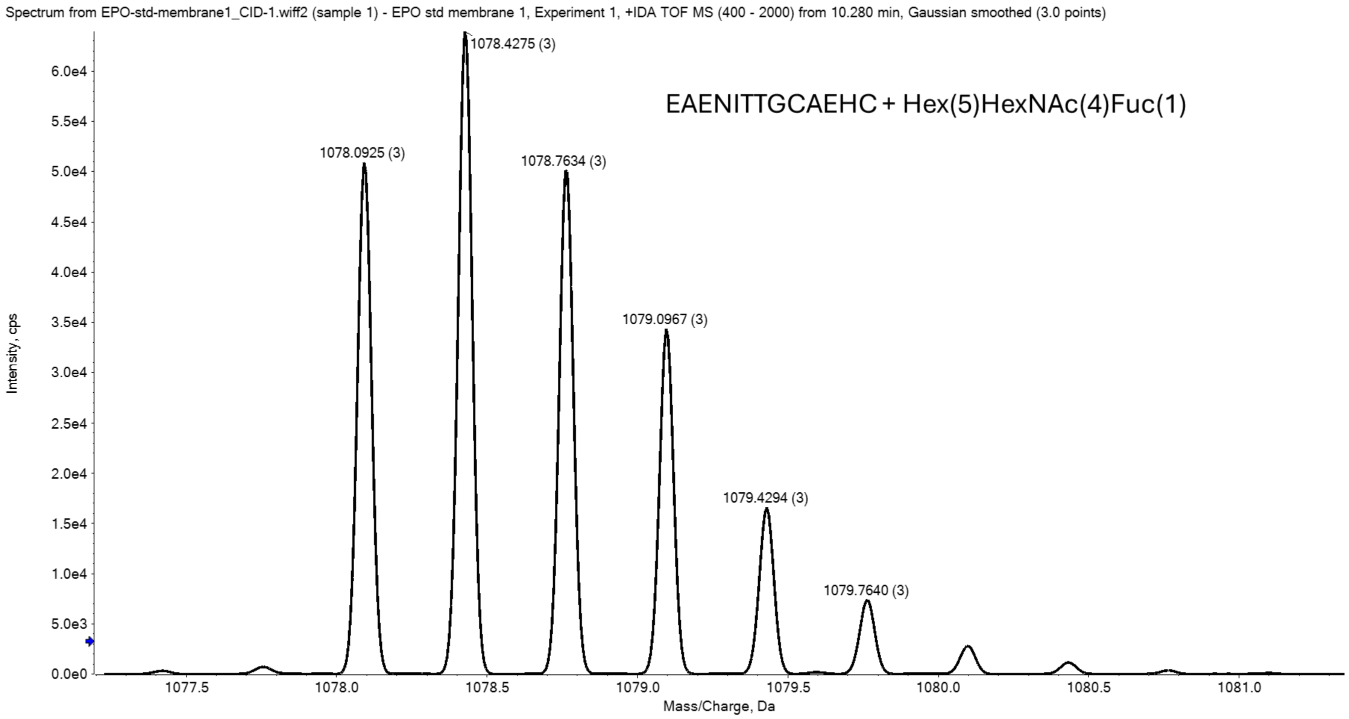

(C)

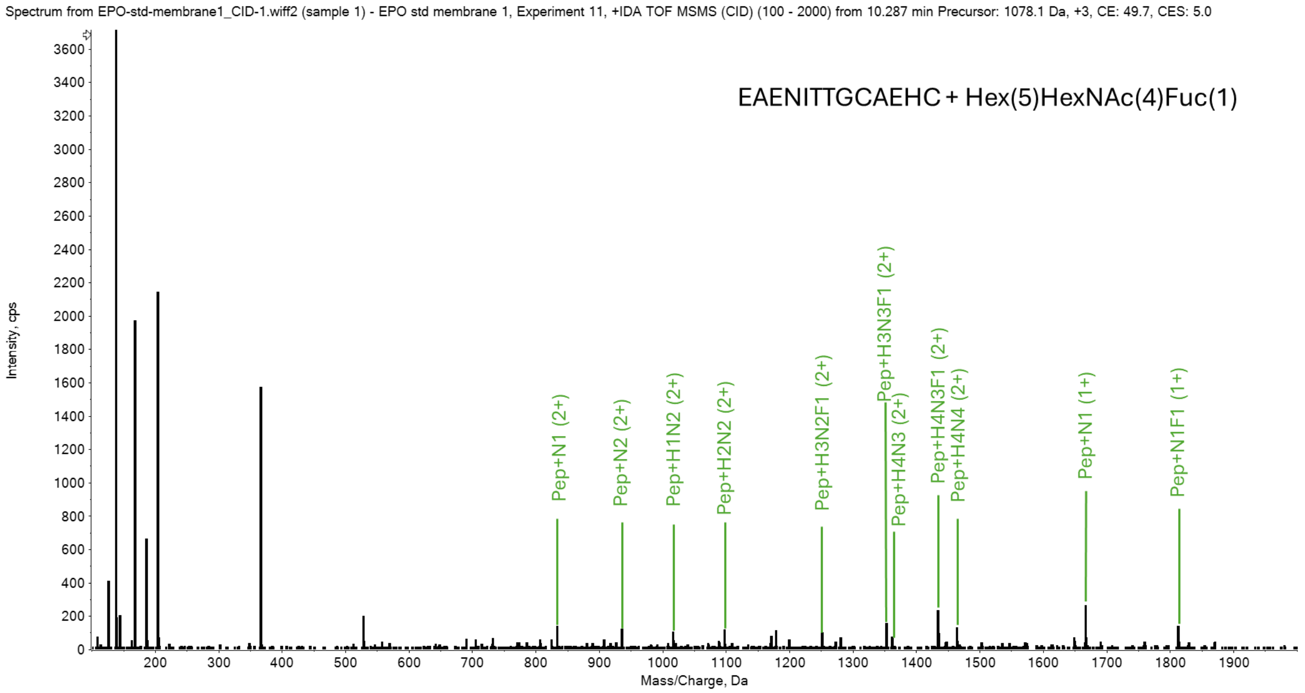

Figure S9. Extracted ion chromatogram (A) and MS1 (B) and MS2 (C) spectra of the EPO glycopeptide EAENITTGCAEHC+Hex(5)HexNAc(4)Fuc(1) with +3 charge.

(A)

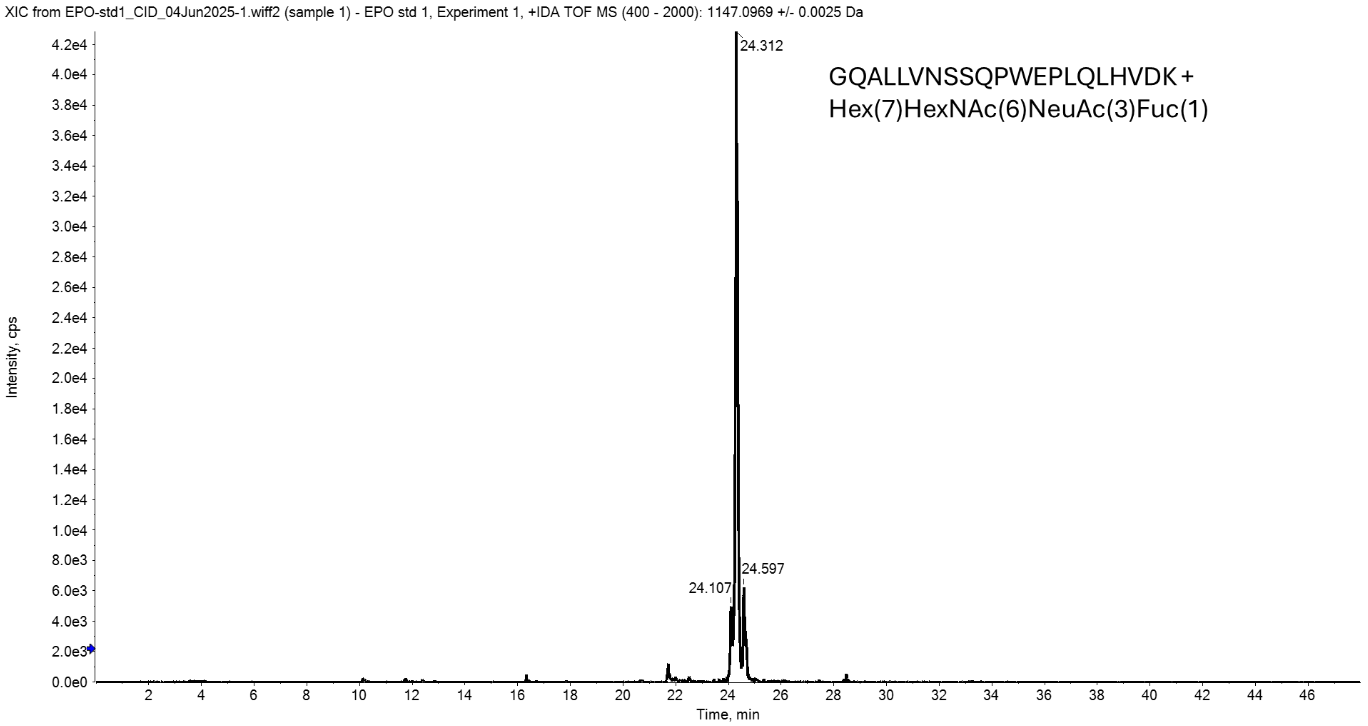
XIC from EPO-std1_CID_04Jun2025-1.wiff2 (sample 1) - EPO std 1, Experiment 1, +IDA TOF MS (400 - 2000): 1147.0969 +/- 0.0025 Da
GQALLVNSSQPWEPLQLHVDK+
Hex(7)HexNAc(6)NeuAc(3)Fuc(1)
24.312
24.107
24.597
Intensity, cps
Time, min

(B)

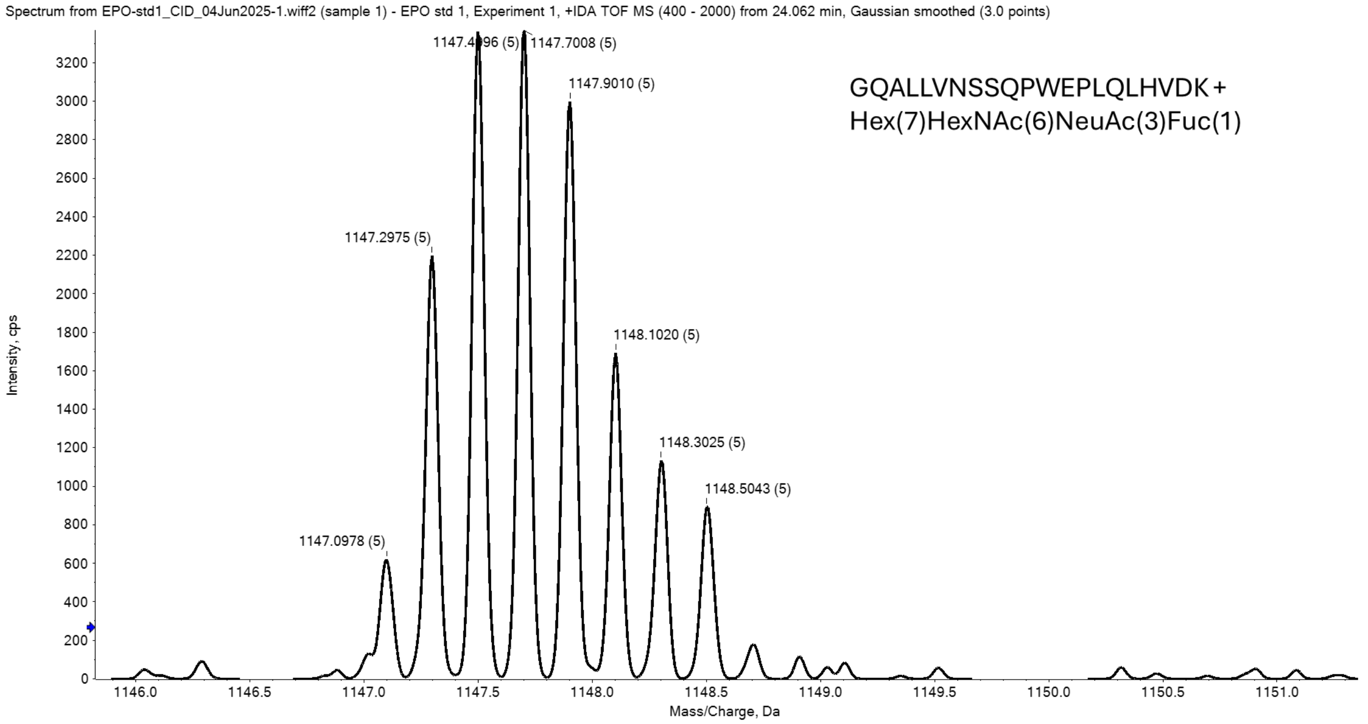
Spectrum from EPO-std1_CID_04Jun2025-1.wiff2 (sample 1) - EPO std 1, Experiment 1, +IDA TOF MS (400 - 2000) from 24.062 min, Gaussian smoothed (3.0 points)
GQALLVNSSQPWEPLQLHVDK+
Hex(7)HexNAc(6)NeuAc(3)Fuc(1)
1147.0978 (5)
1147.2975 (5)
1147.7008 (5)
1147.9010 (5)
1148.1020 (5)
1148.3025 (5)
1148.5043 (5)
Intensity, cps
Mass/Charge, Da

(C)

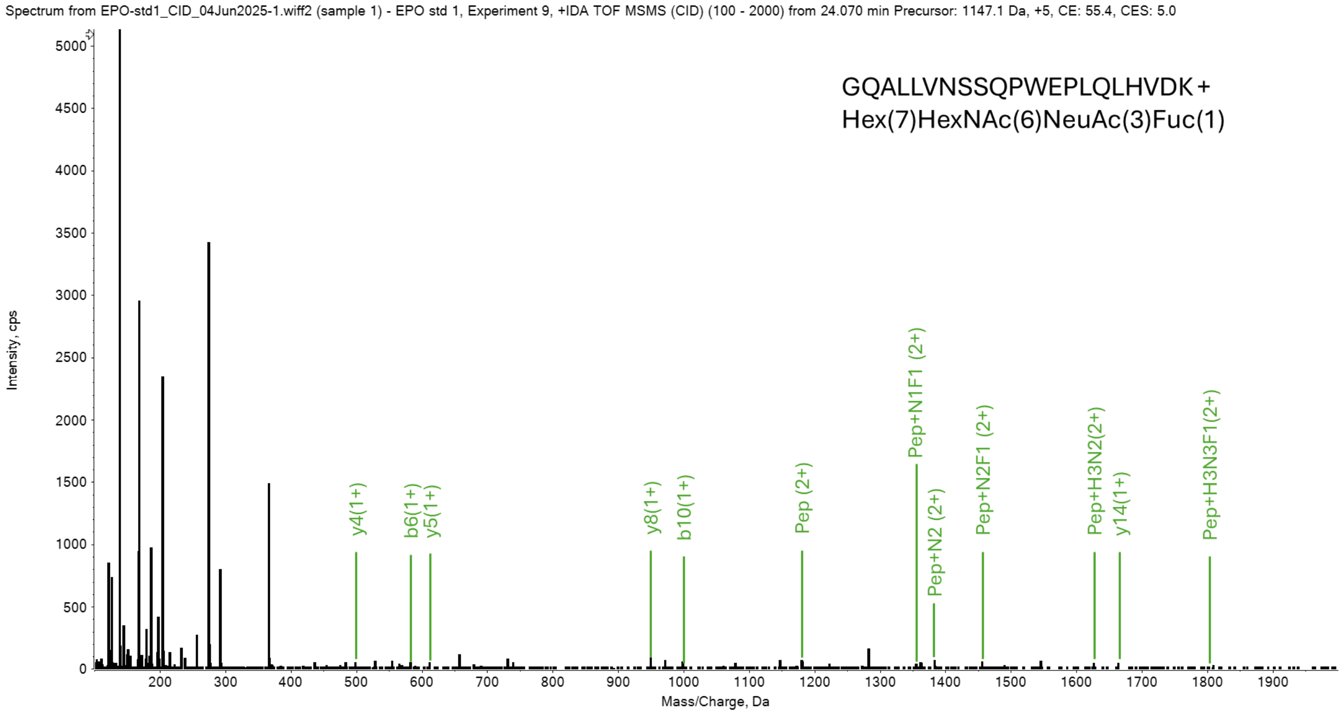

Figure S10. Extracted ion chromatogram (A) and MS1 (B) and MS2 (C) spectra of the EPO glycopeptide GQALLVNSSQPWEPLQLHVDK+Hex(7)HexNAc(6)NeuAc(3)Fuc(1) with +5 charge.

(A)

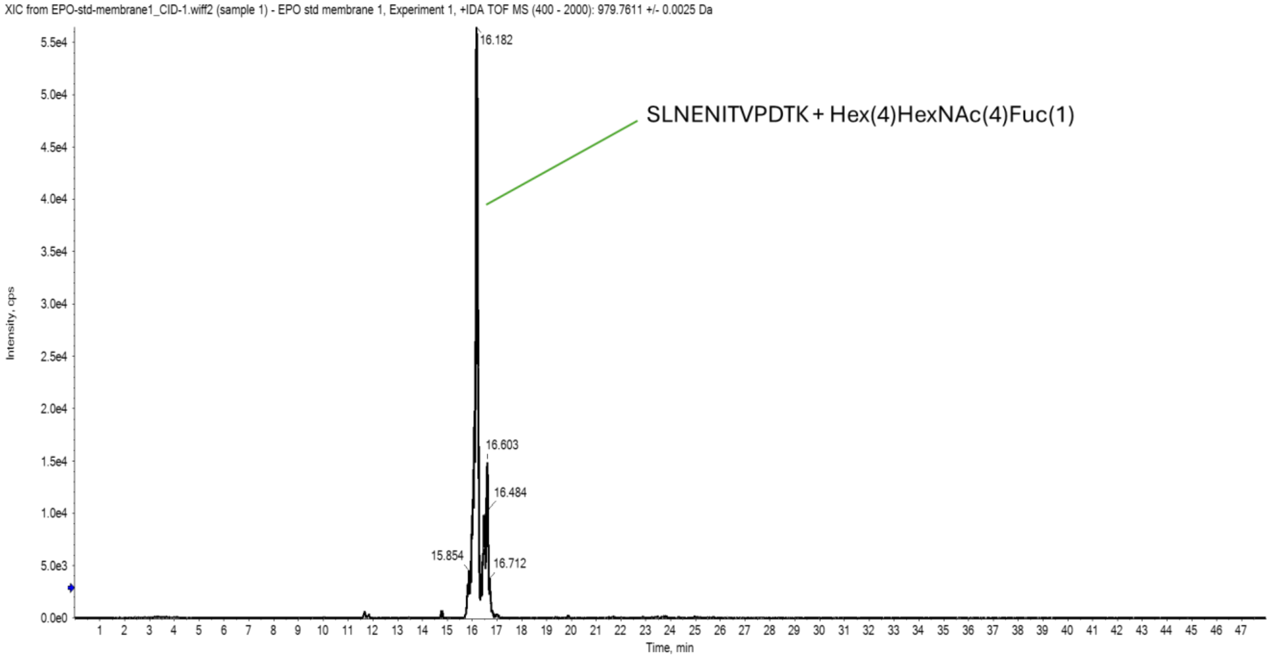

(B)

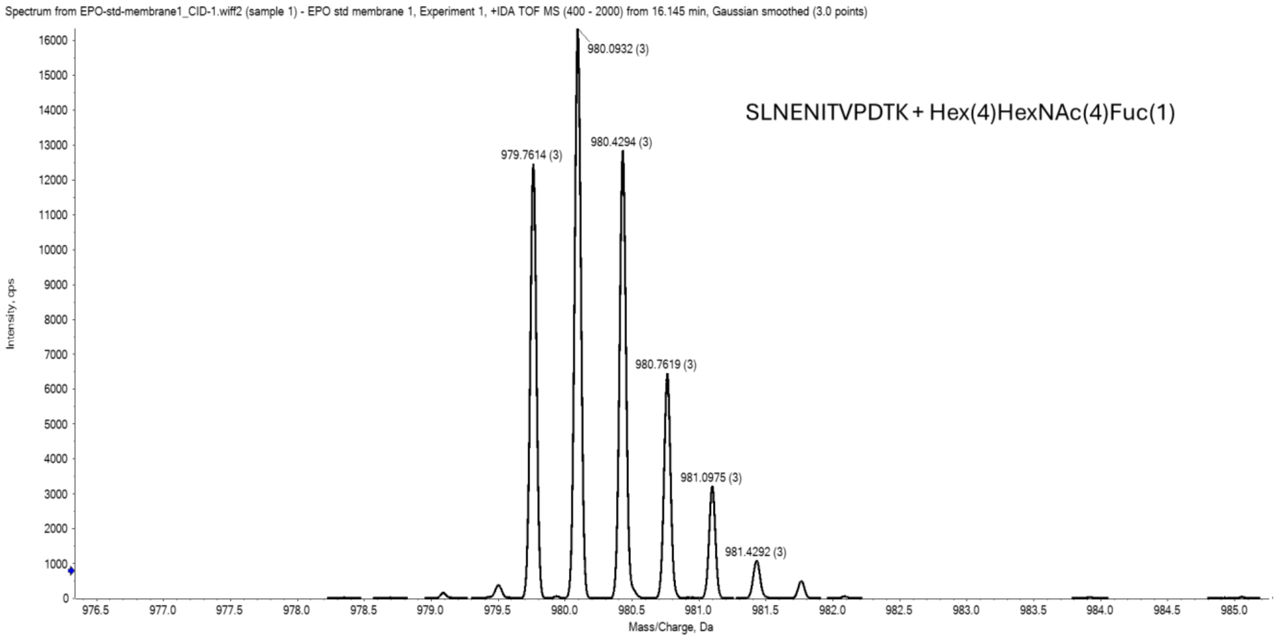

(C)

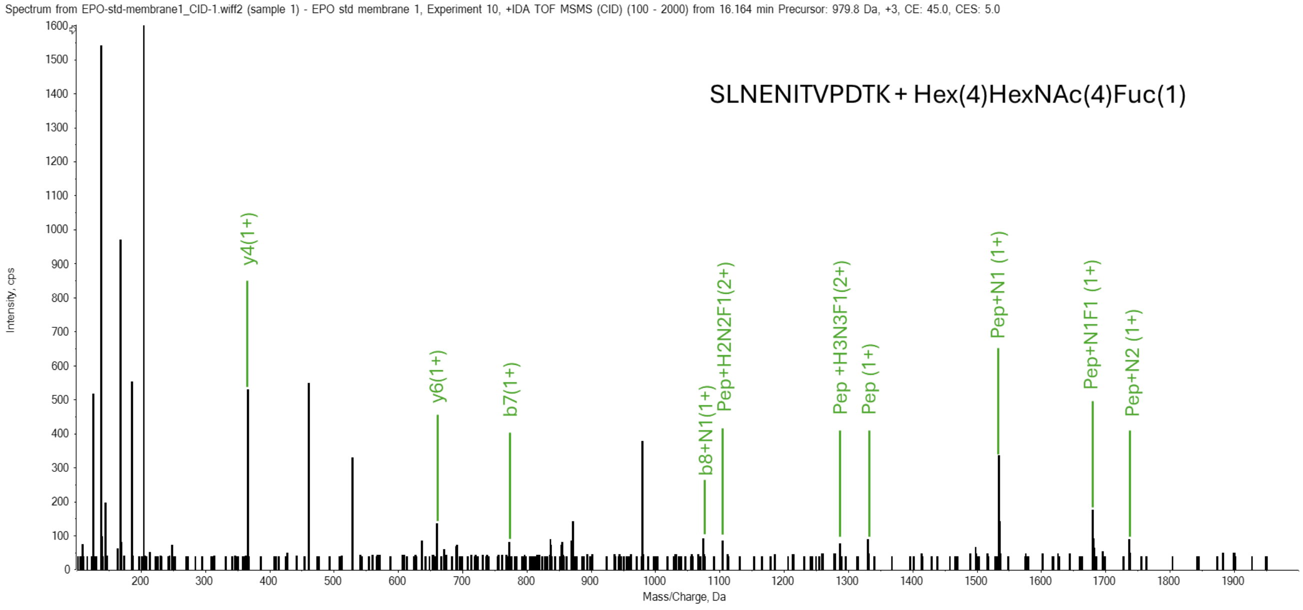

Figure S11. Extracted ion chromatogram (A), MS1 (B) and MS2 (C) spectra of the EPO glycopeptide SLNENITVPDTK+Hex(4)HexNAc(4)Fuc(1) with +3 charge.

Tables S1-S18. EPO N-glycans identified from in-solution trypsin digested samples

Table S1. EPO N-glycopeptides (Asn51) identified from 10 µg of EPO standard after in-solution digestion. This is the analysis of the first replicate solution (Exp#1).

The table shows each glycan only once, and the glycans may appear on multiple peptides with different numbers of missed cleavages and in different charge states. For a particular glycan, we selected the glycopeptide with the highest Glyco Decipher glycan score. This applies to Tables S1-S36.

Peptide	Glycan	Glycan Mass (Da)	Precursor m/z	Precursor charge
EAENITTGCAEHC	Hex(3)HexNAc(3)Fuc(1)	1241.4544	902.366	3
EAENITTGCAEHC	Hex(3)HexNAc(4)Fuc(1)	1444.5338	970.0586	3
EAENITTGCAEHC	Hex(3)HexNAc(5)Fuc(1)	1647.6132	1037.7523	3
EAENITTGC	Hex(3)HexNAc(6)Fuc(1)	1850.6925	1416.0731	2
EAENITTGC	Hex(4)HexNAc(3)Fuc(1)	1403.5073	1192.4785	2
EAENITTGC	Hex(4)HexNAc(3)NeuAc(1)Fuc(1)	1694.6027	1338.0271	2
EAENITTGC	Hex(4)HexNAc(4)Fuc(1)	1606.5866	1294.0177	2
EAENITTGCAEHC	Hex(4)HexNAc(4)NeuAc(1)Fuc(1)	1897.682	841.0832	4
EAENITTGCAEHC	Hex(4)HexNAc(5)Fuc(1)	1809.666	1091.7711	3
EAENITTGC	Hex(4)HexNAc(5)NeuAc(1)Fuc(1)	2100.7614	1027.7395	3
EAENITTGC	Hex(4)HexNAc(6)Fuc(1)	2012.7453	998.4028	3
EAENITTGC	Hex(4)HexNAc(6)NeuAc(1)Fuc(1)	2303.8408	1095.432	3
EAENITTGC	Hex(5)HexNAc(4)Fuc(1)	1768.6394	917.0336	3
EAENITTGC	Hex(5)HexNAc(4)NeuAc(1)	1913.6769	965.3759	3
EAENITTGC	Hex(5)HexNAc(4)NeuAc(1)Fuc(1)	2059.7348	760.7999	4
EAENITTGC	Hex(5)HexNAc(4)NeuAc(2)Fuc(1)	2350.8302	1666.1381	2
EAENITTGC	Hex(5)HexNAc(5)Fuc(1)	1971.7188	738.7953	4
EAENITTGC	Hex(5)HexNAc(5)NeuAc(1)Fuc(1)	2262.8142	1081.7534	3
EAENITTGC	Hex(5)HexNAc(6)NeuAc(1)Fuc(1)	2465.8936	1149.4532	3
EAENITTGCAEHC	Hex(6)HexNAc(5)Fuc(1)	2133.7716	1199.8058	3
EAENITTGC	Hex(6)HexNAc(5)NeuAc(1)Fuc(1)	2424.867	1135.7774	3
EAENITTGC	Hex(6)HexNAc(5)NeuAc(2)Fuc(1)	2715.9624	1232.8089	3
EAENITTGC	Hex(6)HexNAc(5)NeuAc(3)Fuc(1)	3007.0578	1329.835	3
EAENITTGC	Hex(7)HexNAc(6)Fuc(1)	2498.9038	870.593	4
EAENITTGC	Hex(7)HexNAc(6)NeuAc(1)Fuc(1)	2789.9992	1257.4878	3
EAENITTGC	Hex(7)HexNAc(6)NeuAc(3)Fuc(1)	3372.19	1451.5533	3
EAENITTGC	Hex(7)HexNAc(6)NeuAc(4)Fuc(1)	3663.2854	1548.5849	3
EAENITTGC	Hex(8)HexNAc(7)NeuAc(1)Fuc(1)	3155.1314	1379.1965	3

Table S2. EPO N-glycopeptides (Asn65) identified from 10 µg of EPO standard after in-solution digestion. This is the analysis of the first replicate solution (Exp#1).

Peptide	Glycan	Glycan Mass (Da)	Precursor m/z	Precursor charge
SLNENITVPDTK	Hex(3)HexNAc(3)Fuc(1)	1241.4544	1286.5762	2
SLNENITVPDTK	Hex(3)HexNAc(4)Fuc(1)	1444.5338	1388.1156	2
SLNENITVPDTK	Hex(3)HexNAc(5)Fuc(1)	1647.6132	1489.6563	2
SLNENITVPDTK	Hex(3)HexNAc(6)Fuc(1)	1850.6925	1591.193	2
SLNENITVPDTK	Hex(4)HexNAc(4)Fuc(1)	1606.5866	1469.1417	2
SLNENITVPDTK	Hex(4)HexNAc(5)Fuc(1)	1809.666	1570.6827	2
SLNENITVPDTK	Hex(4)HexNAc(5)NeuAc(1)Fuc(1)	2100.7614	1716.2304	2
SLNENITVPDTK	Hex(4)HexNAc(6)Fuc(1)	2012.7453	1672.2192	2
SLNENITVPDTK	Hex(4)HexNAc(6)NeuAc(1)Fuc(1)	2303.8408	1817.7708	2
SLNENITVPDTK	Hex(5)HexNAc(4)Fuc(1)	1768.6394	1033.7833	3
SLNENITVPDTK	Hex(5)HexNAc(4)NeuAc(1)Fuc(1)	2059.7348	1695.7195	2
SLNENITVPDTK	Hex(5)HexNAc(4)NeuAc(2)Fuc(1)	2350.8302	1227.847	3
SLNENITVPDTK	Hex(5)HexNAc(5)Fuc(1)	1971.7188	1651.7066	2
SLNENITVPDTK	Hex(5)HexNAc(5)NeuAc(1)Fuc(1)	2262.8142	1797.2527	2
SLNENITVPDTK	Hex(5)HexNAc(6)Fuc(1)	2174.7982	1753.2482	2
SLNENITVPDTK	Hex(5)HexNAc(6)NeuAc(2)Fuc(1)	2756.989	1022.6781	4
SLNENITVPDTK	Hex(6)HexNAc(5)NeuAc(1)Fuc(1)	2424.867	1252.5273	3
SLNENITVPDTK	Hex(6)HexNAc(6)NeuAc(1)Fuc(1)	2627.9464	1320.2193	3
SLNENITVPDTK	Hex(7)HexNAc(6)Fuc(1)	2498.9038	1915.3023	2
SLNENITVPDTK	Hex(7)HexNAc(6)NeuAc(1)Fuc(1)	2789.9992	1030.9283	4
SLNENITVPDTK	Hex(7)HexNAc(6)NeuAc(2)Fuc(1)	3081.0946	1471.2719	3
SLNENITVPDTK	Hex(7)HexNAc(6)NeuAc(3)Fuc(1)	3372.19	1176.4731	4
SLNENITVPDTK	Hex(7)HexNAc(6)NeuAc(4)Fuc(1)	3663.2854	1665.3324	3
SLNENITVPDTK	Hex(8)HexNAc(7)NeuAc(3)Fuc(1)	3737.3222	1690.0141	3

Table S3. EPO N-glycopeptides (Asn110) identified from 10 µg of EPO standard after in-solution digestion. This is the analysis of the first replicate solution (Exp#1).

Peptide	Glycan	Glycan Mass (Da)	Precursor m/z	Precursor charge
GQALLVNSSQPWEPLQLHVDK	Hex(3)HexNAc(3)Fuc(1)	1241.4544	1200.9073	3
GQALLVNSSQPWEPLQLHVDK	Hex(3)HexNAc(4)Fuc(1)	1444.5338	1268.5958	3
GQALLVNSSQPWEPLQLHVDK	Hex(3)HexNAc(5)Fuc(1)	1647.6132	1002.4732	4
GQALLVNSSQPWEPLQLHVDK	Hex(4)HexNAc(3)Fuc(1)	1403.5073	1254.926	3
GQALLVNSSQPWEPLQLHVDK	Hex(4)HexNAc(4)Fuc(1)	1606.5866	1322.6183	3
GQALLVNSSQPWEPLQLHVDK	Hex(4)HexNAc(4)NeuAc(1)Fuc(1)	1897.682	1419.6483	3
GQALLVNSSQPWEPLQLHVDK	Hex(4)HexNAc(6)NeuAc(1)Fuc(1)	2303.8408	1166.526	4
GQALLVNSSQPWEPLQLHVDK	Hex(5)HexNAc(4)Fuc(1)	1768.6394	1032.7292	4
GQALLVNSSQPWEPLQLHVDK	Hex(5)HexNAc(4)NeuAc(1)Fuc(1)	2059.7348	1473.6694	3
GQALLVNSSQPWEPLQLHVDK	Hex(5)HexNAc(5)Fuc(1)	1971.7188	1083.5004	4
GQALLVNSSQPWEPLQLHVDK	Hex(5)HexNAc(5)NeuAc(2)Fuc(1)	2553.9096	1229.049	4
GQALLVNSSQPWEPLQLHVDK	Hex(5)HexNAc(6)NeuAc(1)Fuc(1)	2465.8936	965.8316	5
GQALLVNSSQPWEPLQLHVDK	Hex(5)HexNAc(6)NeuAc(2)Fuc(1)	2756.989	1279.8138	4
GQALLVNSSQPWEPLQLHVDK	Hex(6)HexNAc(5)Fuc(1)	2133.7716	1498.3496	3
GQALLVNSSQPWEPLQLHVDK	Hex(6)HexNAc(5)NeuAc(1)Fuc(1)	2424.867	957.6285	5
GQALLVNSSQPWEPLQLHVDK	Hex(6)HexNAc(5)NeuAc(2)Fuc(1)	2715.9624	846.7089	6
GQALLVNSSQPWEPLQLHVDK	Hex(6)HexNAc(5)NeuAc(3)Fuc(1)	3007.0578	1789.446	3
GQALLVNSSQPWEPLQLHVDK	Hex(6)HexNAc(6)Fuc(1)	2336.851	940.0274	5
GQALLVNSSQPWEPLQLHVDK	Hex(6)HexNAc(6)NeuAc(3)Fuc(1)	3210.1372	1114.6858	5
GQALLVNSSQPWEPLQLHVDK	Hex(6)HexNAc(7)Fuc(1)	2539.9303	1225.5505	4
GQALLVNSSQPWEPLQLHVDK	Hex(7)HexNAc(6)Fuc(1)	2498.9038	1215.295	4
GQALLVNSSQPWEPLQLHVDK	Hex(7)HexNAc(6)NeuAc(2)Fuc(1)	3081.0946	1360.8403	4
GQALLVNSSQPWEPLQLHVDK	Hex(7)HexNAc(6)NeuAc(3)Fuc(1)	3372.19	1147.0969	5
GQALLVNSSQPWEPLQLHVDK	Hex(7)HexNAc(6)NeuAc(4)Fuc(1)	3663.2854	1004.5973	6
GQALLVNSSQPWEPLQLHVDK	Hex(7)HexNAc(8)NeuAc(1)	3050.1	1353.0952	4
GQALLVNSSQPWEPLQLHVDK	Hex(8)HexNAc(7)Fuc(1)	2864.036	1045.4661	5
GQALLVNSSQPWEPLQLHVDK	Hex(8)HexNAc(7)NeuAc(2)Fuc(1)	3446.2268	1161.9033	5
GQALLVNSSQPWEPLQLHVDK	Hex(8)HexNAc(7)NeuAc(4)Fuc(1)	4028.4176	1278.3411	5

Table S4. EPO N-glycopeptides (Asn51) identified from 10 µg of EPO standard after in-solution digestion. This is the analysis of the second replicate solution (Exp#2).

Peptide	Glycan	Glycan mass	Precursor m/z	Precursor charge
EAENITTGCAEHC	Hex(3)HexNAc(3)Fuc(1)	1241.4544	902.3677	3
EAENITTGC	Hex(3)HexNAc(4)Fuc(1)	1444.5338	1212.9918	2
EAENITTGCAEHC	Hex(3)HexNAc(5)Fuc(1)	1647.6132	1037.756	3
EAENITTGC	Hex(3)HexNAc(6)Fuc(1)	1850.6925	1416.0715	2
EAENITTGC	Hex(4)HexNAc(3)Fuc(1)	1403.5073	1192.477	2
EAENITTGC	Hex(4)HexNAc(3)NeuAc(1)Fuc(1)	1694.6027	1338.0266	2
EAENITTGC	Hex(4)HexNAc(4)Fuc(1)	1606.5866	1294.0176	2
EAENITTGCAEHC	Hex(4)HexNAc(5)Fuc(1)	1809.666	1091.7711	3
EAENITTGC	Hex(4)HexNAc(5)NeuAc(1)Fuc(1)	2100.7614	1027.7408	3
EAENITTGC	Hex(4)HexNAc(6)Fuc(1)	2012.7453	1497.0962	2
EAENITTGC	Hex(4)HexNAc(6)NeuAc(1)Fuc(1)	2303.8408	821.8272	4
EAENITTGC	Hex(5)HexNAc(4)Fuc(1)	1768.6394	1078.096	3
EAENITTGCAEHC	Hex(5)HexNAc(4)NeuAc(1)Fuc(1)	2059.7348	1175.1296	3
EAENITTGC	Hex(5)HexNAc(4)NeuAc(2)Fuc(1)	2350.8302	833.5739	4
EAENITTGCAEHC	Hex(5)HexNAc(5)Fuc(1)	1971.7188	1145.7901	3
EAENITTGC	Hex(5)HexNAc(5)NeuAc(1)Fuc(1)	2262.8142	1081.759	3
EAENITTGCAEHC	Hex(6)HexNAc(5)Fuc(1)	2133.7716	1199.8068	3
EAENITTGC	Hex(6)HexNAc(5)NeuAc(1)Fuc(1)	2424.867	1135.7776	3
EAENITTGC	Hex(6)HexNAc(5)NeuAc(2)Fuc(1)	2715.9624	1045.6514	4
EAENITTGC	Hex(6)HexNAc(5)NeuAc(3)Fuc(1)	3007.0578	1329.8404	3
EAENITTGC	Hex(7)HexNAc(6)Fuc(1)	2498.9038	1160.4559	3
EAENITTGC	Hex(7)HexNAc(6)NeuAc(1)Fuc(1)	2789.9992	1257.4844	3
EAENITTGC	Hex(7)HexNAc(6)NeuAc(2)Fuc(1)	3081.0946	1016.1416	4
EAENITTGC	Hex(7)HexNAc(6)NeuAc(3)Fuc(1)	3372.19	1451.5482	3
EAENITTGC	Hex(8)HexNAc(7)NeuAc(2)Fuc(1)	3446.2268	1476.2289	3
EAENITTGC	Hex(8)HexNAc(7)NeuAc(3)Fuc(1)	3737.3222	1180.1976	4

Table S5. EPO N-glycopeptides (Asn65) identified from 10 μg of EPO standard after in-solution digestion. This is the analysis of the second replicate solution (Exp#2).

Peptide	Glycan	Glycan mass	Precursor m/z	Precursor charge
SLNENITVPDTK	Hex(3)HexNAc(3)Fuc(1)	1241.4544	1286.5791	2
SLNENITVPDTK	Hex(3)HexNAc(4)Fuc(1)	1444.5338	1388.1179	2
SLNENITVPDTK	Hex(3)HexNAc(5)Fuc(1)	1647.6132	1489.6563	2
SLNENITVPDTK	Hex(3)HexNAc(6)Fuc(1)	1850.6925	1591.192	2
SLNENITVPDTK	Hex(4)HexNAc(4)Fuc(1)	1606.5866	1469.1437	2
SLNENITVPDTK	Hex(4)HexNAc(4)NeuAc(1)Fuc(1)	1897.682	1076.7976	3
SLNENITVPDTK	Hex(4)HexNAc(5)Fuc(1)	1809.666	785.842	4
SLNENITVPDTK	Hex(4)HexNAc(6)NeuAc(1)Fuc(1)	2303.8408	1212.1831	3
SLNENITVPDTK	Hex(5)HexNAc(4)Fuc(1)	1768.6394	1033.7833	3
SLNENITVPDTK	Hex(5)HexNAc(5)Fuc(1)	1971.7188	1651.7055	2
SLNENITVPDTK	Hex(5)HexNAc(5)NeuAc(1)Fuc(1)	2262.8142	1797.2587	2
SLNENITVPDTK	Hex(5)HexNAc(5)NeuAc(2)Fuc(1)	2553.9096	1942.8016	2
SLNENITVPDTK	Hex(6)HexNAc(5)NeuAc(1)Fuc(1)	2424.867	1252.528	3
SLNENITVPDTK	Hex(6)HexNAc(5)NeuAc(3)Fuc(1)	3007.0578	1446.5876	3
SLNENITVPDTK	Hex(6)HexNAc(6)Fuc(1)	2336.851	1834.274	2
SLNENITVPDTK	Hex(6)HexNAc(6)NeuAc(1)Fuc(1)	2627.9464	1320.2216	3
SLNENITVPDTK	Hex(7)HexNAc(6)Fuc(1)	2498.9038	1915.3071	2
SLNENITVPDTK	Hex(7)HexNAc(6)NeuAc(1)Fuc(1)	2789.9992	1374.2395	3
SLNENITVPDTK	Hex(7)HexNAc(6)NeuAc(2)Fuc(1)	3081.0946	1471.2639	3
SLNENITVPDTK	Hex(7)HexNAc(6)NeuAc(3)Fuc(1)	3372.19	1176.4744	4
SLNENITVPDTK	Hex(7)HexNAc(6)NeuAc(4)Fuc(1)	3663.2854	1249.253	4
SLNENITVPDTK	Hex(8)HexNAc(7)NeuAc(3)Fuc(1)	3737.3222	1690.0078	3

Table S6. EPO N-glycopeptides (Asn110) identified from 10 µg of EPO standard after in-solution digestion. This is the analysis of the second replicate solution (Exp#2).

Peptide	Glycan	Glycan mass	Precursor m/z	Precursor charge
GQALLVNSSQPWEPLQLHVDK	Hex(3)HexNAc(4)Fuc(1)	1444.5338	1268.6015	3
GQALLVNSSQPWEPLQLHVDK	Hex(4)HexNAc(4)Fuc(1)	1606.5866	992.2168	4
GQALLVNSSQPWEPLQLHVDK	Hex(4)HexNAc(4)NeuAc(1)Fuc(1)	1897.682	1419.6528	3
GQALLVNSSQPWEPLQLHVDK	Hex(4)HexNAc(6)NeuAc(1)Fuc(1)	2303.8408	933.4225	5
GQALLVNSSQPWEPLQLHVDK	Hex(5)HexNAc(4)NeuAc(1)Fuc(1)	2059.7348	1473.6635	3
GQALLVNSSQPWEPLQLHVDK	Hex(5)HexNAc(4)NeuAc(2)Fuc(1)	2350.8302	1522.0133	3
GQALLVNSSQPWEPLQLHVDK	Hex(5)HexNAc(6)Fuc(1)	2174.7982	1570.6991	3
GQALLVNSSQPWEPLQLHVDK	Hex(5)HexNAc(6)NeuAc(2)Fuc(1)	2756.989	1706.0796	3
GQALLVNSSQPWEPLQLHVDK	Hex(5)HexNAc(7)Fuc(1)	2377.8775	1134.2684	4
GQALLVNSSQPWEPLQLHVDK	Hex(6)HexNAc(5)Fuc(1)	2133.7716	1579.7156	3
GQALLVNSSQPWEPLQLHVDK	Hex(6)HexNAc(5)NeuAc(1)Fuc(1)	2424.867	798.193	6
GQALLVNSSQPWEPLQLHVDK	Hex(6)HexNAc(5)NeuAc(2)Fuc(1)	2715.9624	1692.4097	3
GQALLVNSSQPWEPLQLHVDK	Hex(6)HexNAc(5)NeuAc(3)Fuc(1)	3007.0578	1342.3358	4
GQALLVNSSQPWEPLQLHVDK	Hex(6)HexNAc(6)NeuAc(2)Fuc(1)	2919.0418	1056.4659	5
GQALLVNSSQPWEPLQLHVDK	Hex(6)HexNAc(6)NeuAc(3)Fuc(1)	3210.1372	1114.6787	5
GQALLVNSSQPWEPLQLHVDK	Hex(6)HexNAc(7)Fuc(1)	2539.9303	1225.5516	4
GQALLVNSSQPWEPLQLHVDK	Hex(7)HexNAc(6)Fuc(1)	2498.9038	810.5327	6
GQALLVNSSQPWEPLQLHVDK	Hex(7)HexNAc(6)NeuAc(2)Fuc(1)	3081.0946	1088.8779	5
GQALLVNSSQPWEPLQLHVDK	Hex(7)HexNAc(6)NeuAc(3)Fuc(1)	3372.19	956.0817	6
GQALLVNSSQPWEPLQLHVDK	Hex(7)HexNAc(6)NeuAc(4)Fuc(1)	3663.2854	1004.5967	6
GQALLVNSSQPWEPLQLHVDK	Hex(8)HexNAc(7)Fuc(1)	2864.036	1741.7686	3
GQALLVNSSQPWEPLQLHVDK	Hex(8)HexNAc(7)NeuAc(1)Fuc(1)	3155.1314	1103.6855	5
GQALLVNSSQPWEPLQLHVDK	Hex(8)HexNAc(7)NeuAc(2)Fuc(1)	3446.2268	1452.1272	4
GQALLVNSSQPWEPLQLHVDK	Hex(8)HexNAc(7)NeuAc(3)Fuc(1)	3737.3222	1220.1226	5
GQALLVNSSQPWEPLQLHVDK	Hex(9)HexNAc(8)NeuAc(3)Fuc(1)	4102.4544	1293.1401	5

Table S7. EPO N-glycopeptides (Asn51) identified from 10 μg of EPO standard after in-solution digestion. This is the analysis of the third replicate solution (Exp#3).

Peptide	Glycan	Glycan Mass (Da)	Precursor m/z	Precursor charge
EAENITTGC	Hex(3)HexNAc(3)Fuc(1)	1241.4544	1111.4469	2
EAENITTGC	Hex(3)HexNAc(4)Fuc(1)	1444.5338	1212.9883	2
EAENITTGC	Hex(3)HexNAc(5)Fuc(1)	1647.6132	876.6877	3
EAENITTGC	Hex(3)HexNAc(6)Fuc(1)	1850.6925	1416.0684	2
EAENITTGC	Hex(4)HexNAc(3)Fuc(1)	1403.5073	1192.4715	2
EAENITTGC	Hex(4)HexNAc(3)NeuAc(1)Fuc(1)	1694.6027	892.3518	3
EAENITTGC	Hex(4)HexNAc(4)Fuc(1)	1606.5866	863.0115	3
EAENITTGC	Hex(4)HexNAc(4)NeuAc(1)Fuc(1)	1897.682	960.0448	3
EAENITTGC	Hex(4)HexNAc(5)Fuc(1)	1809.666	930.7044	3
EAENITTGC	Hex(4)HexNAc(5)NeuAc(1)Fuc(1)	2100.7614	1027.7366	3
EAENITTGC	Hex(4)HexNAc(6)Fuc(1)	2012.7453	998.3982	3
EAENITTGC	Hex(4)HexNAc(6)NeuAc(1)Fuc(1)	2303.8408	1095.4318	3
EAENITTGC	Hex(5)HexNAc(4)Fuc(1)	1768.6394	917.0303	3
EAENITTGC	Hex(5)HexNAc(4)NeuAc(1)Fuc(1)	2059.7348	1014.0618	3
EAENITTGC	Hex(5)HexNAc(4)NeuAc(2)Fuc(1)	2350.8302	1111.0949	3
EAENITTGC	Hex(5)HexNAc(5)Fuc(1)	1971.7188	984.7216	3
EAENITTGC	Hex(5)HexNAc(5)NeuAc(1)Fuc(1)	2262.8142	1081.7549	3
EAENITTGC	Hex(5)HexNAc(6)Fuc(1)	2174.7982	1052.416	3
EAENITTGC	Hex(5)HexNAc(6)NeuAc(1)Fuc(1)	2465.8936	1149.4424	3
EAENITTGC	Hex(6)HexNAc(5)Fuc(1)	2133.7716	1557.6067	2
EAENITTGC	Hex(6)HexNAc(5)NeuAc(1)Fuc(1)	2424.867	1135.7755	3
EAENITTGC	Hex(6)HexNAc(5)NeuAc(2)Fuc(1)	2715.9624	1232.8065	3
EAENITTGC	Hex(6)HexNAc(5)NeuAc(3)Fuc(1)	3007.0578	1329.8379	3
EAENITTGC	Hex(6)HexNAc(6)Fuc(1)	2336.851	1106.4346	3
EAENITTGC	Hex(6)HexNAc(6)NeuAc(1)Fuc(1)	2627.9464	1203.47	3
EAENITTGC	Hex(7)HexNAc(6)Fuc(1)	2498.9038	1160.4488	3
EAENITTGC	Hex(7)HexNAc(6)NeuAc(1)Fuc(1)	2789.9992	1257.4855	3
EAENITTGC	Hex(7)HexNAc(6)NeuAc(2)Fuc(1)	3081.0946	1016.1352	4
EAENITTGC	Hex(7)HexNAc(6)NeuAc(3)Fuc(1)	3372.19	1451.5519	3
EAENITTGC	Hex(7)HexNAc(6)NeuAc(4)Fuc(1)	3663.2854	1548.5768	3
EAENITTGC	Hex(8)HexNAc(7)NeuAc(4)Fuc(1)	4028.4176	1252.9638	4

Table S8. EPO N-glycopeptides (Asn65) identified from 10 µg of EPO standard after in-solution digestion. This is the analysis of the third replicate solution (Exp#3).

Peptide	Glycan	Glycan Mass (Da)	Precursor m/z	Precursor charge
SLNENITVPDTK	Hex(3)HexNAc(3)Fuc(1)	1241.4544	1286.5728	2
SLNENITVPDTK	Hex(3)HexNAc(4)Fuc(1)	1444.5338	1388.108	2
SLNENITVPDTK	Hex(3)HexNAc(5)Fuc(1)	1647.6132	1489.6491	2
SLNENITVPDTK	Hex(3)HexNAc(6)Fuc(1)	1850.6925	1061.1339	3
SLNENITVPDTK	Hex(4)HexNAc(4)Fuc(1)	1606.5866	1469.1356	2
SLNENITVPDTK	Hex(4)HexNAc(5)Fuc(1)	1809.666	785.8426	4
SLNENITVPDTK	Hex(4)HexNAc(5)NeuAc(1)Fuc(1)	2100.7614	1716.2235	2
SLNENITVPDTK	Hex(4)HexNAc(6)Fuc(1)	2012.7453	1672.2105	2
SLNENITVPDTK	Hex(4)HexNAc(6)NeuAc(1)Fuc(1)	2303.8408	1212.1808	3
SLNENITVPDTK	Hex(5)HexNAc(4)Fuc(1)	1768.6394	1033.7793	3
SLNENITVPDTK	Hex(5)HexNAc(5)Fuc(1)	1971.7188	1651.7088	2
SLNENITVPDTK	Hex(5)HexNAc(5)NeuAc(1)Fuc(1)	2262.8142	1198.5065	3
SLNENITVPDTK	Hex(5)HexNAc(6)NeuAc(2)Fuc(1)	2756.989	1022.6724	4
SLNENITVPDTK	Hex(6)HexNAc(5)Fuc(1)	2133.7716	1732.734	2
SLNENITVPDTK	Hex(6)HexNAc(5)NeuAc(1)Fuc(1)	2424.867	1252.5245	3
SLNENITVPDTK	Hex(6)HexNAc(6)NeuAc(1)Fuc(1)	2627.9464	1320.2158	3
SLNENITVPDTK	Hex(6)HexNAc(6)NeuAc(2)Fuc(1)	2919.0418	1063.1868	4
SLNENITVPDTK	Hex(6)HexNAc(6)NeuAc(3)Fuc(1)	3210.1372	1514.2829	3
SLNENITVPDTK	Hex(7)HexNAc(6)Fuc(1)	2498.9038	1915.2986	2
SLNENITVPDTK	Hex(7)HexNAc(6)NeuAc(1)Fuc(1)	2789.9992	1030.9269	4
SLNENITVPDTK	Hex(7)HexNAc(6)NeuAc(2)Fuc(1)	3081.0946	1103.7036	4
SLNENITVPDTK	Hex(7)HexNAc(6)NeuAc(3)Fuc(1)	3372.19	1568.3	3
SLNENITVPDTK	Hex(7)HexNAc(6)NeuAc(4)Fuc(1)	3663.2854	1665.3325	3
SLNENITVPDTK	Hex(8)HexNAc(7)NeuAc(3)Fuc(1)	3737.3222	1690.0085	3

Table S9. EPO N-glycopeptides (Asn110) identified from 10 µg of EPO standard after in-solution digestion. This is the analysis of the third replicate solution (Exp#3).

Peptide	Glycan	Glycan Mass (Da)	Precursor m/z	Precursor charge
GQALLVNSSQPWEPLQLHVDK	Hex(3)HexNAc(3)Fuc(1)	1241.4544	900.9268	4
GQALLVNSSQPWEPLQLHVDK	Hex(3)HexNAc(4)Fuc(1)	1444.5338	1268.5973	3
GQALLVNSSQPWEPLQLHVDK	Hex(3)HexNAc(5)Fuc(1)	1647.6132	1336.2921	3
GQALLVNSSQPWEPLQLHVDK	Hex(3)HexNAc(6)Fuc(1)	1850.6925	1403.9818	3
GQALLVNSSQPWEPLQLHVDK	Hex(4)HexNAc(4)Fuc(1)	1606.5866	1322.6161	3
GQALLVNSSQPWEPLQLHVDK	Hex(4)HexNAc(4)NeuAc(1)Fuc(1)	1897.682	1064.986	4
GQALLVNSSQPWEPLQLHVDK	Hex(4)HexNAc(5)Fuc(1)	1809.666	1390.3069	3
GQALLVNSSQPWEPLQLHVDK	Hex(4)HexNAc(5)NeuAc(1)Fuc(1)	2100.7614	1115.7549	4
GQALLVNSSQPWEPLQLHVDK	Hex(4)HexNAc(6)NeuAc(1)Fuc(1)	2303.8408	933.4189	5
GQALLVNSSQPWEPLQLHVDK	Hex(5)HexNAc(4)NeuAc(1)Fuc(1)	2059.7348	884.598	5
GQALLVNSSQPWEPLQLHVDK	Hex(5)HexNAc(4)NeuAc(2)Fuc(1)	2350.8302	1178.2716	4
GQALLVNSSQPWEPLQLHVDK	Hex(5)HexNAc(6)NeuAc(1)Fuc(1)	2465.8936	965.8306	5
GQALLVNSSQPWEPLQLHVDK	Hex(6)HexNAc(5)Fuc(1)	2133.7716	1124.009	4
GQALLVNSSQPWEPLQLHVDK	Hex(6)HexNAc(5)NeuAc(1)Fuc(1)	2424.867	1196.7866	4
GQALLVNSSQPWEPLQLHVDK	Hex(6)HexNAc(5)NeuAc(2)Fuc(1)	2715.9624	1692.4084	3
GQALLVNSSQPWEPLQLHVDK	Hex(6)HexNAc(5)NeuAc(3)Fuc(1)	3007.0578	1342.3339	4
GQALLVNSSQPWEPLQLHVDK	Hex(6)HexNAc(6)NeuAc(1)Fuc(1)	2627.9464	1247.5521	4
GQALLVNSSQPWEPLQLHVDK	Hex(6)HexNAc(6)NeuAc(3)Fuc(1)	3210.1372	1393.1012	4
GQALLVNSSQPWEPLQLHVDK	Hex(7)HexNAc(6)Fuc(1)	2498.9038	972.4394	5
GQALLVNSSQPWEPLQLHVDK	Hex(7)HexNAc(6)NeuAc(1)Fuc(1)	2789.9992	1288.0679	4
GQALLVNSSQPWEPLQLHVDK	Hex(7)HexNAc(6)NeuAc(2)Fuc(1)	3081.0946	1360.8433	4
GQALLVNSSQPWEPLQLHVDK	Hex(7)HexNAc(6)NeuAc(3)Fuc(1)	3372.19	1147.0937	5
GQALLVNSSQPWEPLQLHVDK	Hex(7)HexNAc(6)NeuAc(4)Fuc(1)	3663.2854	1205.3146	5
GQALLVNSSQPWEPLQLHVDK	Hex(7)HexNAc(7)NeuAc(2)Fuc(1)	3284.174	1411.6092	4
GQALLVNSSQPWEPLQLHVDK	Hex(8)HexNAc(7)Fuc(1)	2864.036	1306.5697	4
GQALLVNSSQPWEPLQLHVDK	Hex(8)HexNAc(7)NeuAc(1)Fuc(1)	3155.1314	1103.6801	5
GQALLVNSSQPWEPLQLHVDK	Hex(8)HexNAc(7)NeuAc(2)Fuc(1)	3446.2268	1161.9007	5
GQALLVNSSQPWEPLQLHVDK	Hex(8)HexNAc(7)NeuAc(4)Fuc(1)	4028.4176	1278.3386	5
GQALLVNSSQPWEPLQLHVDK	Hex(9)HexNAc(8)NeuAc(3)Fuc(1)	4102.4544	1293.1485	5

Table S10. EPO N-glycopeptides (Asn51) identified from 10 µg of EPO spiked into CHO cell supernatant and recovered using the affinity-peptide membrane prior to in-solution digestion. This is the analysis of the first replicate solution (Exp#1).

Peptide	Glycan	Glycan Mass (Da)	Precursor m/z	Precursor charge
EAENITTGC	Hex(3)HexNAc(3)Fuc(1)	1241.4544	1111.4458	2
EAENITTGC	Hex(3)HexNAc(4)Fuc(1)	1444.5338	1212.986	2
EAENITTGC	Hex(3)HexNAc(5)Fuc(1)	1647.6132	876.6853	3
EAENITTGC	Hex(3)HexNAc(6)Fuc(1)	1850.6925	1416.0666	2
EAENITTGC	Hex(4)HexNAc(3)Fuc(1)	1403.5073	795.318	3
EAENITTGC	Hex(4)HexNAc(3)NeuAc(1)Fuc(1)	1694.6027	1338.0202	2
EAENITTGC	Hex(4)HexNAc(4)Fuc(1)	1606.5866	863.0116	3
EAENITTGC	Hex(4)HexNAc(4)NeuAc(1)Fuc(1)	1897.682	1439.5572	2
EAENITTGC	Hex(4)HexNAc(5)Fuc(1)	1809.666	930.7035	3
EAENITTGC	Hex(4)HexNAc(5)NeuAc(1)Fuc(1)	2100.7614	1027.7349	3
EAENITTGC	Hex(4)HexNAc(6)Fuc(1)	2012.7453	998.3943	3
EAENITTGC	Hex(5)HexNAc(4)Fuc(1)	1768.6394	917.0266	3
EAENITTGC	Hex(5)HexNAc(4)NeuAc(1)Fuc(1)	2059.7348	1520.589	2
EAENITTGC	Hex(5)HexNAc(4)NeuAc(2)Fuc(1)	2350.8302	1111.0951	3
EAENITTGC	Hex(5)HexNAc(5)Fuc(1)	1971.7188	984.7225	3
EAENITTGC	Hex(5)HexNAc(5)NeuAc(1)Fuc(1)	2262.8142	1081.7546	3
EAENITTGC	Hex(5)HexNAc(6)Fuc(1)	2174.7982	1052.4157	3
EAENITTGC	Hex(6)HexNAc(5)Fuc(1)	2133.7716	1038.742	3
EAENITTGC	Hex(6)HexNAc(5)NeuAc(1)Fuc(1)	2424.867	1135.7734	3
EAENITTGC	Hex(6)HexNAc(5)NeuAc(2)Fuc(1)	2715.9624	1232.8048	3
EAENITTGC	Hex(6)HexNAc(5)NeuAc(3)Fuc(1)	3007.0578	1329.836	3
EAENITTGC	Hex(7)HexNAc(6)NeuAc(1)Fuc(1)	2789.9992	943.3611	4
EAENITTGC	Hex(7)HexNAc(6)NeuAc(2)Fuc(1)	3081.0946	1016.138	4
EAENITTGC	Hex(8)HexNAc(7)NeuAc(3)Fuc(1)	3737.3222	1180.192	4
EAENITTGC	Hex(8)HexNAc(7)NeuAc(4)Fuc(1)	4028.4176	1252.9706	4

Table S11. EPO N-glycopeptides (Asn65) identified from 10 µg of EPO spiked into CHO cell supernatant and recovered using the affinity-peptide membrane prior to in-solution digestion. This is the analysis of the first replicate solution (Exp#1).

Peptide	Glycan	Glycan Mass (Da)	Precursor m/z	Precursor charge
SLNENITVPDTK	Hex(3)HexNAc(3)Fuc(1)	1241.4544	1286.5719	2
SLNENITVPDTK	Hex(3)HexNAc(4)Fuc(1)	1444.5338	1388.1115	2
SLNENITVPDTK	Hex(3)HexNAc(5)Fuc(1)	1647.6132	993.4384	3
SLNENITVPDTK	Hex(3)HexNAc(6)Fuc(1)	1850.6925	796.0978	4
SLNENITVPDTK	Hex(4)HexNAc(4)Fuc(1)	1606.5866	735.0726	4
SLNENITVPDTK	Hex(4)HexNAc(4)NeuAc(1)	1751.6241	1541.6527	2
SLNENITVPDTK	Hex(4)HexNAc(5)Fuc(1)	1809.666	785.8412	4
SLNENITVPDTK	Hex(4)HexNAc(6)Fuc(1)	2012.7453	836.6122	4
SLNENITVPDTK	Hex(4)HexNAc(6)NeuAc(1)Fuc(1)	2303.8408	1212.1832	3
SLNENITVPDTK	Hex(5)HexNAc(4)Fuc(1)	1768.6394	1033.78	3
SLNENITVPDTK	Hex(5)HexNAc(4)NeuAc(2)Fuc(1)	2350.8302	1227.8423	3
SLNENITVPDTK	Hex(5)HexNAc(5)Fuc(1)	1971.7188	1651.7022	2
SLNENITVPDTK	Hex(5)HexNAc(5)NeuAc(1)Fuc(1)	2262.8142	1198.4989	3
SLNENITVPDTK	Hex(5)HexNAc(6)NeuAc(1)Fuc(1)	2465.8936	1266.1986	3
SLNENITVPDTK	Hex(5)HexNAc(6)NeuAc(2)Fuc(1)	2756.989	1363.2278	3
SLNENITVPDTK	Hex(6)HexNAc(5)NeuAc(1)Fuc(1)	2424.867	1252.5237	3
SLNENITVPDTK	Hex(6)HexNAc(5)NeuAc(2)Fuc(1)	2715.9624	1349.5565	3
SLNENITVPDTK	Hex(7)HexNAc(6)Fuc(1)	2498.9038	1277.2041	3
SLNENITVPDTK	Hex(7)HexNAc(6)NeuAc(1)Fuc(1)	2789.9992	1374.2363	3
SLNENITVPDTK	Hex(7)HexNAc(6)NeuAc(2)Fuc(1)	3081.0946	1103.7005	4
SLNENITVPDTK	Hex(7)HexNAc(6)NeuAc(3)Fuc(1)	3372.19	941.3797	5
SLNENITVPDTK	Hex(7)HexNAc(6)NeuAc(4)Fuc(1)	3663.2854	999.6014	5
SLNENITVPDTK	Hex(9)HexNAc(8)NeuAc(3)Fuc(1)	4102.4544	1811.7266	3

Table S12. EPO N-glycopeptides (Asn110) identified from 10 µg of EPO spiked into CHO cell supernatant and recovered using the affinity-peptide membrane prior to in-solution digestion. This is the analysis of the first replicate solution (Exp#1).

Peptide	Glycan	Glycan Mass (Da)	Precursor m/z	Precursor charge
GQALLVNSSQPWEPLQLHVDK	Hex(3)HexNAc(4)Fuc(1)	1444.5338	1268.5931	3
GQALLVNSSQPWEPLQLHVDK	Hex(3)HexNAc(5)Fuc(1)	1647.6132	1336.2905	3
GQALLVNSSQPWEPLQLHVDK	Hex(4)HexNAc(3)Fuc(1)	1403.5073	1254.9241	3
GQALLVNSSQPWEPLQLHVDK	Hex(4)HexNAc(4)Fuc(1)	1606.5866	1322.6166	3
GQALLVNSSQPWEPLQLHVDK	Hex(4)HexNAc(4)NeuAc(1)Fuc(1)	1897.682	1419.647	3
GQALLVNSSQPWEPLQLHVDK	Hex(4)HexNAc(5)NeuAc(1)Fuc(1)	2100.7614	1487.3373	3
GQALLVNSSQPWEPLQLHVDK	Hex(4)HexNAc(6)Fuc(1)	2012.7453	1093.7522	4
GQALLVNSSQPWEPLQLHVDK	Hex(5)HexNAc(4)Fuc(1)	1768.6394	1376.635	3
GQALLVNSSQPWEPLQLHVDK	Hex(5)HexNAc(4)NeuAc(1)Fuc(1)	2059.7348	1105.5003	4
GQALLVNSSQPWEPLQLHVDK	Hex(5)HexNAc(4)NeuAc(2)Fuc(1)	2350.8302	1570.6932	3
GQALLVNSSQPWEPLQLHVDK	Hex(5)HexNAc(6)Fuc(1)	2174.7982	1134.2647	4
GQALLVNSSQPWEPLQLHVDK	Hex(5)HexNAc(6)NeuAc(2)Fuc(1)	2756.989	853.5448	6
GQALLVNSSQPWEPLQLHVDK	Hex(6)HexNAc(5)Fuc(1)	2133.7716	1498.3442	3
GQALLVNSSQPWEPLQLHVDK	Hex(6)HexNAc(5)NeuAc(1)Fuc(1)	2424.867	957.6266	5
GQALLVNSSQPWEPLQLHVDK	Hex(6)HexNAc(5)NeuAc(2)Fuc(1)	2715.9624	846.7055	6
GQALLVNSSQPWEPLQLHVDK	Hex(6)HexNAc(5)NeuAc(3)Fuc(1)	3007.0578	1342.3292	4
GQALLVNSSQPWEPLQLHVDK	Hex(6)HexNAc(6)NeuAc(1)Fuc(1)	2627.9464	1247.5546	4
GQALLVNSSQPWEPLQLHVDK	Hex(7)HexNAc(6)Fuc(1)	2498.9038	1620.0572	3
GQALLVNSSQPWEPLQLHVDK	Hex(7)HexNAc(6)NeuAc(1)Fuc(1)	2789.9992	1288.0652	4
GQALLVNSSQPWEPLQLHVDK	Hex(7)HexNAc(6)NeuAc(2)Fuc(1)	3081.0946	1360.8362	4
GQALLVNSSQPWEPLQLHVDK	Hex(7)HexNAc(6)NeuAc(3)Fuc(1)	3372.19	1147.0938	5
GQALLVNSSQPWEPLQLHVDK	Hex(7)HexNAc(6)NeuAc(4)Fuc(1)	3663.2854	1205.313	5
GQALLVNSSQPWEPLQLHVDK	Hex(8)HexNAc(7)NeuAc(2)Fuc(1)	3446.2268	1452.1221	4
GQALLVNSSQPWEPLQLHVDK	Hex(9)HexNAc(8)NeuAc(3)Fuc(1)	4102.4544	1077.7866	6

Table S13. EPO N-glycopeptides (Asn51) identified from 10 μg of EPO spiked into CHO cell supernatant and recovered using the affinity-peptide membrane prior to in-solution digestion. This is the analysis of the second replicate solution (Exp#2).

Peptide	Glycan	Glycan Mass (Da)	Precursor m/z	Precursor charge
EAENITTGC	Hex(3)HexNAc(3)Fuc(1)	1241.4544	741.2995	3
EAENITTGC	Hex(3)HexNAc(4)Fuc(1)	1444.5338	1212.9849	2
EAENITTGC	Hex(3)HexNAc(5)Fuc(1)	1647.6132	876.6856	3
EAENITTGC	Hex(3)HexNAc(6)Fuc(1)	1850.6925	1416.0652	2
EAENITTGC	Hex(4)HexNAc(3)Fuc(1)	1403.5073	1192.4713	2
EAENITTGC	Hex(4)HexNAc(4)Fuc(1)	1606.5866	1294.0149	2
EAENITTGCAEHC	Hex(4)HexNAc(4)NeuAc(1)Fuc(1)	1897.682	841.0854	4
EAENITTGC	Hex(4)HexNAc(5)Fuc(1)	1809.666	930.701	3
EAENITTGC	Hex(4)HexNAc(5)NeuAc(1)Fuc(1)	2100.7614	1027.7347	3
EAENITTGC	Hex(5)HexNAc(4)Fuc(1)	1768.6394	917.0286	3
EAENITTGC	Hex(5)HexNAc(4)NeuAc(1)Fuc(1)	2059.7348	760.7945	4
EAENITTGC	Hex(5)HexNAc(4)NeuAc(2)Fuc(1)	2350.8302	833.57	4
EAENITTGC	Hex(5)HexNAc(5)Fuc(1)	1971.7188	984.7218	3
EAENITTGC	Hex(5)HexNAc(5)NeuAc(1)Fuc(1)	2262.8142	1622.1234	2
EAENITTGC	Hex(5)HexNAc(6)Fuc(1)	2174.7982	1052.4161	3
EAENITTGC	Hex(6)HexNAc(5)Fuc(1)	2133.7716	779.3066	4
EAENITTGC	Hex(6)HexNAc(5)NeuAc(1)Fuc(1)	2424.867	1135.7711	3
EAENITTGC	Hex(6)HexNAc(5)NeuAc(2)Fuc(1)	2715.9624	1232.8064	3
EAENITTGC	Hex(6)HexNAc(5)NeuAc(3)Fuc(1)	3007.0578	1329.8363	3
EAENITTGC	Hex(7)HexNAc(6)Fuc(1)	2498.9038	870.5889	4
EAENITTGC	Hex(7)HexNAc(6)NeuAc(1)Fuc(1)	2789.9992	1257.4852	3
EAENITTGC	Hex(7)HexNAc(6)NeuAc(2)Fuc(1)	3081.0946	1354.5172	3
EAENITTGC	Hex(7)HexNAc(6)NeuAc(3)Fuc(1)	3372.19	1088.9116	4
EAENITTGC	Hex(7)HexNAc(6)NeuAc(4)Fuc(1)	3663.2854	1161.6848	4
EAENITTGC	Hex(8)HexNAc(7)NeuAc(3)Fuc(1)	3737.3222	1180.1956	4

Table S14. EPO N-glycopeptides (Asn65) identified from 10 µg of EPO spiked into CHO cell supernatant and recovered using the affinity-peptide membrane prior to in-solution digestion. This is the analysis of the second replicate solution (Exp#2).

Peptide	Glycan	Glycan Mass (Da)	Precursor m/z	Precursor charge
SLNENITVPDTK	Hex(3)HexNAc(3)Fuc(1)	1241.4544	1286.573	2
SLNENITVPDTK	Hex(3)HexNAc(4)Fuc(1)	1444.5338	925.7436	3
SLNENITVPDTK	Hex(3)HexNAc(5)Fuc(1)	1647.6132	993.4381	3
SLNENITVPDTK	Hex(3)HexNAc(6)Fuc(1)	1850.6925	796.0984	4
SLNENITVPDTK	Hex(4)HexNAc(4)Fuc(1)	1606.5866	979.7644	3
SLNENITVPDTK	Hex(4)HexNAc(4)NeuAc(1)Fuc(1)	1897.682	1614.6804	2
SLNENITVPDTK	Hex(4)HexNAc(5)Fuc(1)	1809.666	785.8422	4
SLNENITVPDTK	Hex(4)HexNAc(6)NeuAc(1)Fuc(1)	2303.8408	909.3864	4
SLNENITVPDTK	Hex(5)HexNAc(4)Fuc(1)	1768.6394	1033.7814	3
SLNENITVPDTK	Hex(5)HexNAc(5)Fuc(1)	1971.7188	1651.7076	2
SLNENITVPDTK	Hex(5)HexNAc(5)NeuAc(1)Fuc(1)	2262.8142	1198.5031	3
SLNENITVPDTK	Hex(5)HexNAc(6)NeuAc(1)Fuc(1)	2465.8936	1266.196	3
SLNENITVPDTK	Hex(5)HexNAc(6)NeuAc(2)Fuc(1)	2756.989	1363.2261	3
SLNENITVPDTK	Hex(6)HexNAc(5)NeuAc(1)Fuc(1)	2424.867	1252.5232	3
SLNENITVPDTK	Hex(6)HexNAc(5)NeuAc(3)Fuc(1)	3007.0578	1085.1929	4
SLNENITVPDTK	Hex(6)HexNAc(6)Fuc(1)	2336.851	917.6409	4
SLNENITVPDTK	Hex(6)HexNAc(6)NeuAc(1)Fuc(1)	2627.9464	1320.2089	3
SLNENITVPDTK	Hex(7)HexNAc(6)Fuc(1)	2498.9038	1277.1991	3
SLNENITVPDTK	Hex(7)HexNAc(6)NeuAc(1)Fuc(1)	2789.9992	1374.2346	3
SLNENITVPDTK	Hex(7)HexNAc(6)NeuAc(2)Fuc(1)	3081.0946	1471.2684	3
SLNENITVPDTK	Hex(7)HexNAc(6)NeuAc(3)Fuc(1)	3372.19	1568.3007	3
SLNENITVPDTK	Hex(7)HexNAc(6)NeuAc(4)Fuc(1)	3663.2854	1665.3324	3

Table S15. EPO N-glycopeptides (Asn110) identified from 10 μg of EPO spiked into CHO cell supernatant and recovered using the affinity-peptide membrane prior to in-solution digestion. This is the analysis of the second replicate solution (Exp#2).

Peptide	Glycan	Glycan Mass (Da)	Precursor m/z	Precursor charge
GQALLVNSSQPWEPLQLHVDK	Hex(3)HexNAc(4)Fuc(1)	1444.5338	1268.599	3
GQALLVNSSQPWEPLQLHVDK	Hex(4)HexNAc(3)Fuc(1)	1403.5073	1254.9269	3
GQALLVNSSQPWEPLQLHVDK	Hex(4)HexNAc(4)Fuc(1)	1606.5866	992.2123	4
GQALLVNSSQPWEPLQLHVDK	Hex(4)HexNAc(4)NeuAc(1)Fuc(1)	1897.682	1419.652	3
GQALLVNSSQPWEPLQLHVDK	Hex(4)HexNAc(5)Fuc(1)	1809.666	1390.3098	3
GQALLVNSSQPWEPLQLHVDK	Hex(4)HexNAc(6)Fuc(1)	2012.7453	1093.7543	4
GQALLVNSSQPWEPLQLHVDK	Hex(4)HexNAc(6)NeuAc(1)Fuc(1)	2303.8408	933.42	5
GQALLVNSSQPWEPLQLHVDK	Hex(5)HexNAc(4)Fuc(1)	1768.6394	1032.7261	4
GQALLVNSSQPWEPLQLHVDK	Hex(5)HexNAc(4)NeuAc(1)Fuc(1)	2059.7348	1105.5005	4
GQALLVNSSQPWEPLQLHVDK	Hex(5)HexNAc(4)NeuAc(2)Fuc(1)	2350.8302	1570.6937	3
GQALLVNSSQPWEPLQLHVDK	Hex(5)HexNAc(5)NeuAc(1)Fuc(1)	2262.8142	1541.3548	3
GQALLVNSSQPWEPLQLHVDK	Hex(5)HexNAc(6)Fuc(1)	2174.7982	1134.2663	4
GQALLVNSSQPWEPLQLHVDK	Hex(5)HexNAc(6)NeuAc(2)Fuc(1)	2756.989	1279.8088	4
GQALLVNSSQPWEPLQLHVDK	Hex(6)HexNAc(5)Fuc(1)	2133.7716	899.409	5
GQALLVNSSQPWEPLQLHVDK	Hex(6)HexNAc(5)NeuAc(1)Fuc(1)	2424.867	1196.7864	4
GQALLVNSSQPWEPLQLHVDK	Hex(6)HexNAc(5)NeuAc(2)Fuc(1)	2715.9624	1015.8469	5
GQALLVNSSQPWEPLQLHVDK	Hex(6)HexNAc(5)NeuAc(3)Fuc(1)	3007.0578	1342.333	4
GQALLVNSSQPWEPLQLHVDK	Hex(6)HexNAc(6)NeuAc(3)Fuc(1)	3210.1372	1114.677	5
GQALLVNSSQPWEPLQLHVDK	Hex(7)HexNAc(6)NeuAc(1)Fuc(1)	2789.9992	1288.0675	4
GQALLVNSSQPWEPLQLHVDK	Hex(7)HexNAc(6)NeuAc(2)Fuc(1)	3081.0946	1360.8417	4
GQALLVNSSQPWEPLQLHVDK	Hex(7)HexNAc(6)NeuAc(3)Fuc(1)	3372.19	956.0783	6
GQALLVNSSQPWEPLQLHVDK	Hex(7)HexNAc(6)NeuAc(4)Fuc(1)	3663.2854	1004.5934	6
GQALLVNSSQPWEPLQLHVDK	Hex(8)HexNAc(7)NeuAc(1)Fuc(1)	3155.1314	1103.683	5

Table S16. EPO N-glycopeptides (Asn51) identified from 10 µg of EPO spiked into CHO cell supernatant and recovered using the affinity-peptide membrane prior to in-solution digestion. This is the analysis of the third replicate solution (Exp#3).

Peptide	Glycan	Glycan Mass (Da)	Precursor m/z	Precursor charge
EAENITTGC	Hex(3)HexNAc(3)Fuc(1)	1241.4544	1111.4534	2
EAENITTGC	Hex(3)HexNAc(4)Fuc(1)	1444.5338	808.9972	3
EAENITTGC	Hex(3)HexNAc(5)Fuc(1)	1647.6132	876.6908	3
EAENITTGC	Hex(3)HexNAc(6)Fuc(1)	1850.6925	1416.0646	2
EAENITTGC	Hex(4)HexNAc(3)Fuc(1)	1403.5073	795.3218	3
EAENITTGC	Hex(4)HexNAc(3)NeuAc(1)Fuc(1)	1694.6027	1338.0256	2
EAENITTGCAEHC	Hex(4)HexNAc(4)Fuc(1)	1606.5866	1535.6127	2
EAENITTGC	Hex(4)HexNAc(4)NeuAc(1)Fuc(1)	1897.682	1439.5624	2
EAENITTGCAEHC	Hex(4)HexNAc(5)Fuc(1)	1809.666	1091.773	3
EAENITTGC	Hex(5)HexNAc(4)Fuc(1)	1768.6394	1375.044	2
EAENITTGC	Hex(5)HexNAc(4)NeuAc(1)Fuc(1)	2059.7348	1520.595	2
EAENITTGC	Hex(5)HexNAc(4)NeuAc(2)Fuc(1)	2350.8302	1111.0946	3
EAENITTGC	Hex(5)HexNAc(5)Fuc(1)	1971.7188	738.7966	4
EAENITTGC	Hex(5)HexNAc(5)NeuAc(1)Fuc(1)	2262.8142	1081.7589	3
EAENITTGC	Hex(5)HexNAc(6)Fuc(1)	2174.7982	1052.4157	3
EAENITTGC	Hex(6)HexNAc(5)Fuc(1)	2133.7716	1557.6143	2
EAENITTGCAEHC	Hex(6)HexNAc(5)NeuAc(1)Fuc(1)	2424.867	1296.8416	3
EAENITTGC	Hex(6)HexNAc(5)NeuAc(2)Fuc(1)	2715.9624	1232.8079	3
EAENITTGCAEHC	Hex(6)HexNAc(5)NeuAc(3)Fuc(1)	3007.0578	1329.8425	3
EAENITTGCAEHC	Hex(7)HexNAc(6)Fuc(1)	2498.9038	991.389	4
EAENITTGC	Hex(7)HexNAc(6)NeuAc(1)Fuc(1)	2789.9992	1257.4847	3
EAENITTGC	Hex(7)HexNAc(6)NeuAc(2)Fuc(1)	3081.0946	1016.1367	4

Table S17. EPO N-glycopeptides (Asn65) identified from 10 µg of EPO spiked into CHO cell supernatant and recovered using the affinity-peptide membrane prior to in-solution digestion. This is the analysis of the third replicate solution (Exp#3).

Peptide	Glycan	Glycan Mass (Da)	Precursor m/z	Precursor charge
SLNENITVPDTK	Hex(3)HexNAc(3)Fuc(1)	1241.4544	1286.579	2
SLNENITVPDTK	Hex(3)HexNAc(4)Fuc(1)	1444.5338	1388.1187	2
SLNENITVPDTK	Hex(3)HexNAc(5)Fuc(1)	1647.6132	1489.6558	2
SLNENITVPDTK	Hex(3)HexNAc(6)Fuc(1)	1850.6925	1591.1972	2
SLNENITVPDTK	Hex(4)HexNAc(3)Fuc(1)	1403.5073	1367.6011	2
SLNENITVPDTK	Hex(4)HexNAc(4)Fuc(1)	1606.5866	1469.1461	2
SLNENITVPDTK	Hex(4)HexNAc(4)NeuAc(1)Fuc(1)	1897.682	807.8506	4
SLNENITVPDTK	Hex(4)HexNAc(5)Fuc(1)	1809.666	1047.459	3
SLNENITVPDTK	Hex(4)HexNAc(6)NeuAc(1)Fuc(1)	2303.8408	1212.1822	3
SLNENITVPDTK	Hex(5)HexNAc(4)Fuc(1)	1768.6394	1033.7834	3
SLNENITVPDTK	Hex(5)HexNAc(4)NeuAc(2)Fuc(1)	2350.8302	1227.8487	3
SLNENITVPDTK	Hex(5)HexNAc(5)Fuc(1)	1971.7188	1651.7094	2
SLNENITVPDTK	Hex(5)HexNAc(5)NeuAc(1)Fuc(1)	2262.8142	1198.5087	3
SLNENITVPDTK	Hex(6)HexNAc(5)NeuAc(1)Fuc(1)	2424.867	1252.5231	3
SLNENITVPDTK	Hex(6)HexNAc(5)NeuAc(3)Fuc(1)	3007.0578	1446.5833	3
SLNENITVPDTK	Hex(6)HexNAc(6)Fuc(1)	2336.851	1223.1888	3
SLNENITVPDTK	Hex(6)HexNAc(6)NeuAc(3)Fuc(1)	3210.1372	1514.2815	3
SLNENITVPDTK	Hex(7)HexNAc(6)Fuc(1)	2498.9038	1915.3059	2
SLNENITVPDTK	Hex(7)HexNAc(6)NeuAc(1)Fuc(1)	2789.9992	1374.2395	3
SLNENITVPDTK	Hex(7)HexNAc(6)NeuAc(2)Fuc(1)	3081.0946	1471.2693	3
SLNENITVPDTK	Hex(7)HexNAc(6)NeuAc(3)Fuc(1)	3372.19	1568.297	3

Table S18. EPO N-glycopeptides (Asn110) identified from 10 µg of EPO spiked into CHO cell supernatant and recovered using the affinity-peptide membrane prior to in-solution digestion. This is the analysis of the third replicate solution (Exp#3).

Peptide	Glycan	Glycan Mass (Da)	Precursor m/z	Precursor charge
GQALLVNSSQPWEPLQLHVDK	Hex(4)HexNAc(3)Fuc(1)	1403.5073	1254.9264	3
GQALLVNSSQPWEPLQLHVDK	Hex(4)HexNAc(4)Fuc(1)	1606.5866	1322.6138	3
GQALLVNSSQPWEPLQLHVDK	Hex(4)HexNAc(4)NeuAc(1)Fuc(1)	1897.682	1419.6484	3
GQALLVNSSQPWEPLQLHVDK	Hex(4)HexNAc(5)Fuc(1)	1809.666	1390.3139	3
GQALLVNSSQPWEPLQLHVDK	Hex(4)HexNAc(5)NeuAc(1)	1954.7035	1079.2434	4
GQALLVNSSQPWEPLQLHVDK	Hex(4)HexNAc(5)NeuAc(1)Fuc(1)	2100.7614	1115.7581	4
GQALLVNSSQPWEPLQLHVDK	Hex(4)HexNAc(6)Fuc(1)	2012.7453	1458.0053	3
GQALLVNSSQPWEPLQLHVDK	Hex(5)HexNAc(4)Fuc(1)	1768.6394	826.3843	5
GQALLVNSSQPWEPLQLHVDK	Hex(5)HexNAc(4)NeuAc(1)Fuc(1)	2059.7348	1473.6652	3
GQALLVNSSQPWEPLQLHVDK	Hex(5)HexNAc(4)NeuAc(2)Fuc(1)	2350.8302	1178.2777	4
GQALLVNSSQPWEPLQLHVDK	Hex(5)HexNAc(5)NeuAc(2)Fuc(1)	2553.9096	819.6985	6
GQALLVNSSQPWEPLQLHVDK	Hex(6)HexNAc(5)Fuc(1)	2133.7716	899.4122	5
GQALLVNSSQPWEPLQLHVDK	Hex(6)HexNAc(5)NeuAc(1)Fuc(1)	2424.867	1196.788	4
GQALLVNSSQPWEPLQLHVDK	Hex(6)HexNAc(5)NeuAc(2)Fuc(1)	2715.9624	1015.8509	5
GQALLVNSSQPWEPLQLHVDK	Hex(6)HexNAc(5)NeuAc(3)Fuc(1)	3007.0578	1342.332	4
GQALLVNSSQPWEPLQLHVDK	Hex(6)HexNAc(6)Fuc(1)	2336.851	1174.7821	4
GQALLVNSSQPWEPLQLHVDK	Hex(6)HexNAc(6)NeuAc(3)Fuc(1)	3210.1372	1857.4577	3
GQALLVNSSQPWEPLQLHVDK	Hex(7)HexNAc(6)NeuAc(2)Fuc(1)	3081.0946	1088.8732	5
GQALLVNSSQPWEPLQLHVDK	Hex(7)HexNAc(6)NeuAc(3)Fuc(1)	3372.19	1147.0965	5
GQALLVNSSQPWEPLQLHVDK	Hex(7)HexNAc(6)NeuAc(4)Fuc(1)	3663.2854	1004.5971	6

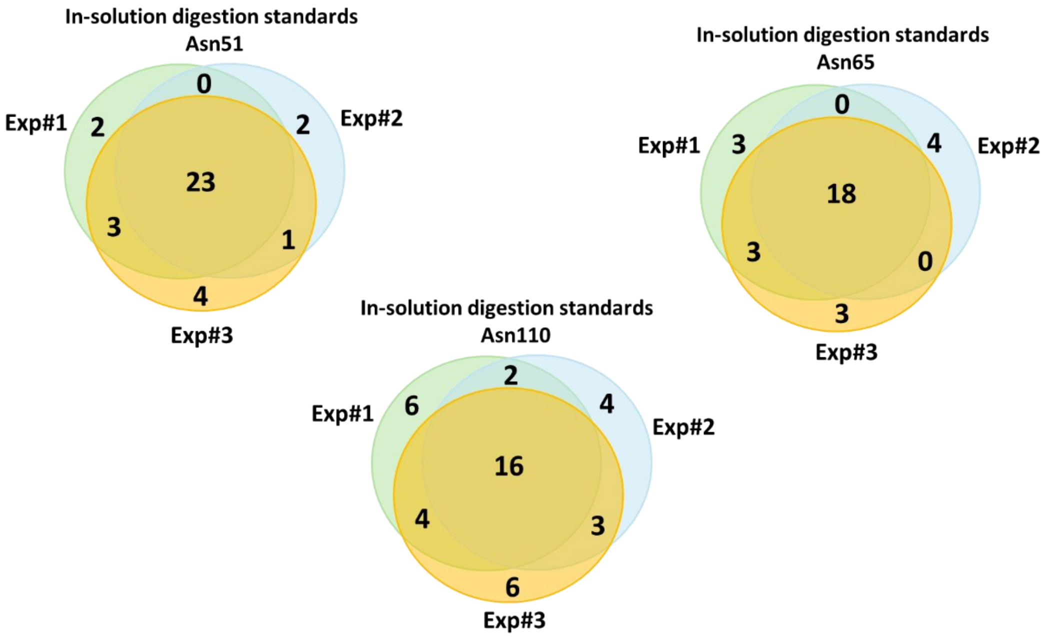

Figure S12. Overlap of glycans identified from EPO standards after three replicate in-solution tryptic digestions.

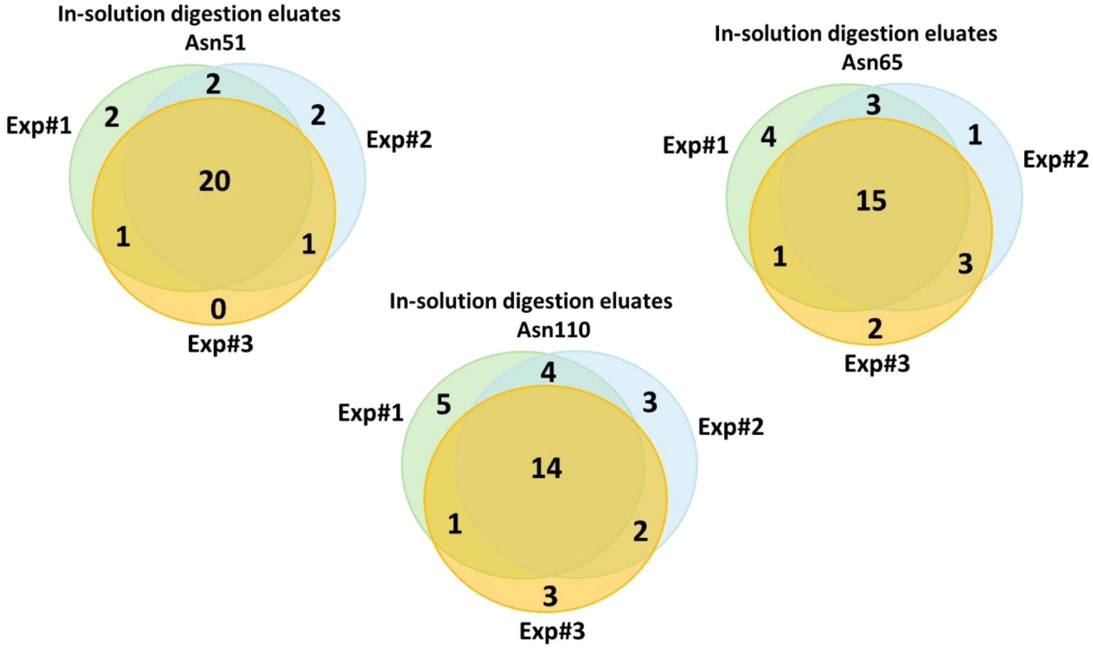

Figure S13. Overlap of glycans identified from recovered EPO after three replicate in-solution tryptic digestions.

Tables S19-S36. EPO N-glycans identified from in-membrane tryptic digested samples

Table S19. EPO N-glycopeptides (Asn51) identified from 10 µg of EPO standard after in-membrane digestion. This is the analysis of the first replicate solution (Exp#1).

Peptide	Glycan	Glycan Mass (Da)	Precursor m/z	Precursor charge
EAENITTGCAEHC	Hex(3)HexNAc(3)Fuc(1)	1241.4544	902.3651	3
EAENITTGCAEHC	Hex(3)HexNAc(4)Fuc(1)	1444.5338	970.0556	3
EAENITTGCAEHC	Hex(3)HexNAc(5)Fuc(1)	1647.6132	1037.7494	3
EAENITTGCAEHC	Hex(4)HexNAc(3)Fuc(1)	1403.5073	956.3826	3
EAENITTGCAEHC	Hex(4)HexNAc(3)NeuAc(1)Fuc(1)	1694.6027	1053.4149	3
EAENITTGCAEHC	Hex(4)HexNAc(4)Fuc(1)	1606.5866	1024.0731	3
EAENITTGCAEHC	Hex(4)HexNAc(4)NeuAc(1)Fuc(1)	1897.682	1121.1049	3
EAENITTGCAEHC	Hex(4)HexNAc(5)Fuc(1)	1809.666	1091.766	3
EAENITTGCAEHC	Hex(4)HexNAc(5)NeuAc(1)Fuc(1)	2100.7614	1188.8047	3
EAENITTGCAEHC	Hex(5)HexNAc(4)Fuc(1)	1768.6394	1078.092	3
EAENITTGCAEHC	Hex(5)HexNAc(4)NeuAc(1)Fuc(1)	2059.7348	1762.1806	2
EAENITTGCAEHC	Hex(5)HexNAc(4)NeuAc(2)Fuc(1)	2350.8302	1272.1571	3
EAENITTGCAEHC	Hex(5)HexNAc(5)Fuc(1)	1971.7188	1145.7808	3
EAENITTGCAEHC	Hex(5)HexNAc(5)NeuAc(1)Fuc(1)	2262.8142	1242.817	3
EAENITTGCAEHC	Hex(5)HexNAc(5)NeuAc(2)Fuc(1)	2553.9096	1005.1371	4
EAENITTGCAEHC	Hex(5)HexNAc(6)Fuc(1)	2174.7982	1213.4736	3
EAENITTGCAEHC	Hex(6)HexNAc(4)Fuc(1)	1930.6923	1132.1109	3
EAENITTGCAEHC	Hex(6)HexNAc(5)Fuc(1)	2133.7716	1199.8028	3
EAENITTGCAEHC	Hex(6)HexNAc(5)NeuAc(1)Fuc(1)	2424.867	1296.834	3
EAENITTGCAEHC	Hex(6)HexNAc(5)NeuAc(2)Fuc(1)	2715.9624	1393.8638	3
EAENITTGC	Hex(6)HexNAc(5)NeuAc(3)Fuc(1)	3007.0578	1329.8417	3
EAENITTGCAEHC	Hex(6)HexNAc(6)Fuc(1)	2336.851	950.8703	4
EAENITTGCAEHC	Hex(6)HexNAc(6)NeuAc(1)Fuc(1)	2627.9464	1364.5284	3
EAENITTGCAEHC	Hex(7)HexNAc(6)Fuc(1)	2498.9038	1321.5146	3
EAENITTGCAEHC	Hex(7)HexNAc(6)NeuAc(1)Fuc(1)	2789.9992	1064.1607	4
EAENITTGCAEHC	Hex(7)HexNAc(6)NeuAc(2)Fuc(1)	3081.0946	1136.9328	4
EAENITTGCAEHC	Hex(7)HexNAc(6)NeuAc(3)Fuc(1)	3372.19	1612.6078	3

Table S20. EPO N-glycopeptides (Asn65) identified from 10 µg of EPO standard after in-membrane digestion. This is the analysis of the first replicate solution (Exp#1).

Peptide	Glycan	Glycan Mass (Da)	Precursor m/z	Precursor charge
SLNENITVPDTK	Hex(3)HexNAc(3)Fuc(1)	1241.4544	858.0491	3
SLNENITVPDTKVNFYAWK	Hex(3)HexNAc(4)Fuc(1)	1444.5338	1228.5565	3
SLNENITVPDTK	Hex(3)HexNAc(5)Fuc(1)	1647.6132	1489.6535	2
SLNENITVPDTK	Hex(3)HexNAc(6)Fuc(1)	1850.6925	1061.1354	3
SLNENITVPDTK	Hex(4)HexNAc(3)Fuc(1)	1403.5073	1367.5975	2
SLNENITVPDTK	Hex(4)HexNAc(3)NeuAc(1)Fuc(1)	1694.6027	1513.1442	2
SLNENITVPDTK	Hex(4)HexNAc(4)Fuc(1)	1606.5866	1469.1351	2
SLNENITVPDTK	Hex(4)HexNAc(4)NeuAc(1)Fuc(1)	1897.682	1076.7929	3
SLNENITVPDTK	Hex(4)HexNAc(5)Fuc(1)	1809.666	785.8426	4
SLNENITVPDTK	Hex(4)HexNAc(5)NeuAc(1)Fuc(1)	2100.7614	1144.4837	3
SLNENITVPDTK	Hex(4)HexNAc(6)Fuc(1)	2012.7453	1672.2163	2
SLNENITVPDTKVNFYAWKR	Hex(5)HexNAc(4)Fuc(1)	1768.6394	1041.7208	4
SLNENITVPDTK	Hex(5)HexNAc(4)NeuAc(1)Fuc(1)	2059.7348	1130.812	3
SLNENITVPDTK	Hex(5)HexNAc(4)NeuAc(2)Fuc(1)	2350.8302	1227.8421	3
SLNENITVPDTK	Hex(5)HexNAc(5)Fuc(1)	1971.7188	1651.7032	2
SLNENITVPDTK	Hex(5)HexNAc(5)NeuAc(1)Fuc(1)	2262.8142	1198.504	3
SLNENITVPDTK	Hex(6)HexNAc(5)Fuc(1)	2133.7716	1732.7275	2
SLNENITVPDTK	Hex(6)HexNAc(5)NeuAc(1)Fuc(1)	2424.867	1878.2715	2
SLNENITVPDTK	Hex(6)HexNAc(5)NeuAc(2)Fuc(1)	2715.9624	1349.5557	3
SLNENITVPDTK	Hex(6)HexNAc(5)NeuAc(3)Fuc(1)	3007.0578	1446.5853	3
SLNENITVPDTK	Hex(6)HexNAc(6)Fuc(1)	2336.851	1223.1841	3
SLNENITVPDTK	Hex(6)HexNAc(6)NeuAc(2)Fuc(1)	2919.0418	1417.2444	3
SLNENITVPDTK	Hex(7)HexNAc(6)Fuc(1)	2498.9038	1915.2953	2
SLNENITVPDTK	Hex(7)HexNAc(6)NeuAc(1)Fuc(1)	2789.9992	1374.2301	3
SLNENITVPDTK	Hex(7)HexNAc(6)NeuAc(2)Fuc(1)	3081.0946	1103.7006	4
SLNENITVPDTK	Hex(7)HexNAc(6)NeuAc(4)Fuc(1)	3663.2854	1665.3271	3
SLNENITVPDTK	Hex(8)HexNAc(7)NeuAc(1)Fuc(1)	3155.1314	1495.9385	3

Table S21. EPO N-glycopeptides (Asn110) identified from 10 µg of EPO standard after in-membrane digestion. This is the analysis of the first replicate solution (Exp#1).

Peptide	Glycan	Glycan Mass (Da)	Precursor m/z	Precursor charge
GQALLVNSSQPWEPLQLHVDK	Hex(3)HexNAc(4)Fuc(1)	1444.5338	1268.5959	3
GQALLVNSSQPWEPLQLHVDK	Hex(3)HexNAc(5)Fuc(1)	1647.6132	1336.289	3
GQALLVNSSQPWEPLQLHVDK	Hex(4)HexNAc(3)Fuc(1)	1403.5073	1254.9197	3
GQALLVNSSQPWEPLQLHVDK	Hex(4)HexNAc(3)NeuAc(1)Fuc(1)	1694.6027	1351.9559	3
GQALLVNSSQPWEPLQLHVDK	Hex(4)HexNAc(4)Fuc(1)	1606.5866	1322.6153	3
GQALLVNSSQPWEPLQLHVDK	Hex(4)HexNAc(4)NeuAc(1)Fuc(1)	1897.682	1419.6463	3
GQALLVNSSQPWEPLQLHVDK	Hex(4)HexNAc(5)NeuAc(1)Fuc(1)	2100.7614	1487.3339	3
GQALLVNSSQPWEPLQLHVDK	Hex(4)HexNAc(6)Fuc(1)	2012.7453	1458.0071	3
GQALLVNSSQPWEPLQLHVDK	Hex(5)HexNAc(4)Fuc(1)	1768.6394	1032.7281	4
GQALLVNSSQPWEPLQLHVDK	Hex(5)HexNAc(4)NeuAc(1)Fuc(1)	2059.7348	1473.6638	3
GQALLVNSSQPWEPLQLHVDKAVSGLR	Hex(5)HexNAc(4)NeuAc(2)Fuc(1)	2350.8302	1324.1086	4
GQALLVNSSQPWEPLQLHVDK	Hex(5)HexNAc(5)Fuc(1)	1971.7188	1083.4967	4
GQALLVNSSQPWEPLQLHVDKAVSGLR	Hex(5)HexNAc(5)NeuAc(1)Fuc(1)	2262.8142	1302.1051	4
GQALLVNSSQPWEPLQLHVDKAVSGLR	Hex(5)HexNAc(5)NeuAc(2)Fuc(1)	2553.9096	1100.1079	5
GQALLVNSSQPWEPLQLHVDKAVSGLR	Hex(6)HexNAc(5)Fuc(1)	2133.7716	1498.3446	3
GQALLVNSSQPWEPLQLHVDK	Hex(6)HexNAc(5)NeuAc(1)Fuc(1)	2424.867	1196.7848	4
GQALLVNSSQPWEPLQLHVDKAVSGLR	Hex(6)HexNAc(5)NeuAc(2)Fuc(1)	2715.9624	1886.8492	3
GQALLVNSSQPWEPLQLHVDKAVSGLR	Hex(6)HexNAc(5)NeuAc(3)Fuc(1)	3007.0578	1190.7361	5
GQALLVNSSQPWEPLQLHVDK	Hex(6)HexNAc(6)Fuc(1)	2336.851	1174.7842	4
GQALLVNSSQPWEPLQLHVDK	Hex(6)HexNAc(6)NeuAc(2)Fuc(1)	2919.0418	1760.0926	3
GQALLVNSSQPWEPLQLHVDK	Hex(7)HexNAc(6)Fuc(1)	2498.9038	1215.291	4
GQALLVNSSQPWEPLQLHVDKAVSGLR	Hex(7)HexNAc(6)NeuAc(1)Fuc(1)	2789.9992	1147.3199	5
GQALLVNSSQPWEPLQLHVDK	Hex(7)HexNAc(6)NeuAc(2)Fuc(1)	3081.0946	1814.1151	3
GQALLVNSSQPWEPLQLHVDKAVSGLR	Hex(7)HexNAc(6)NeuAc(3)Fuc(1)	3372.19	1263.7607	5
GQALLVNSSQPWEPLQLHVDK	Hex(7)HexNAc(6)NeuAc(4)Fuc(1)	3663.2854	1205.3122	5
GQALLVNSSQPWEPLQLHVDK	Hex(8)HexNAc(7)Fuc(1)	2864.036	1306.5799	4

Table S22. EPO N-glycopeptides (Asn51) identified from 10 µg of EPO standard after in-membrane digestion. This is the analysis of the second replicate solution (Exp#2).

Peptide	Glycan	Glycan Mass (Da)	Precursor m/z	Precursor charge
EAENITTGCAEHC	Hex(3)HexNAc(3)Fuc(1)	1241.4544	902.3638	3
EAENITTGCAEHC	Hex(3)HexNAc(4)Fuc(1)	1444.5338	970.0573	3
EAENITTGCAEHC	Hex(3)HexNAc(5)Fuc(1)	1647.6132	1037.7483	3
YLLEAKEAENITTGCAEHC	Hex(4)HexNAc(3)Fuc(1)	1403.5073	896.8892	4
EAENITTGCAEHC	Hex(4)HexNAc(4)Fuc(1)	1606.5866	1024.0741	3
EAENITTGCAEHC	Hex(4)HexNAc(4)NeuAc(1)Fuc(1)	1897.682	1121.1083	3
EAENITTGCAEHC	Hex(4)HexNAc(5)Fuc(1)	1809.666	1091.7667	3
EAENITTGCAEHC	Hex(4)HexNAc(5)NeuAc(1)Fuc(1)	2100.7614	1188.8023	3
EAENITTGCAEHC	Hex(5)HexNAc(4)Fuc(1)	1768.6394	1078.0884	3
EAENITTGCAEHC	Hex(5)HexNAc(4)NeuAc(1)Fuc(1)	2059.7348	1175.125	3
EAENITTGCAEHC	Hex(5)HexNAc(4)NeuAc(2)Fuc(1)	2350.8302	1272.158	3
YLLEAKEAENITTGCAEHC	Hex(5)HexNAc(5)Fuc(1)	1971.7188	1038.9443	4
EAENITTGCAEHC	Hex(5)HexNAc(5)NeuAc(1)Fuc(1)	2262.8142	1242.8159	3
YLLEAKEAENITTGCAEHC	Hex(6)HexNAc(4)Fuc(1)	1930.6923	823.1533	5
YLLEAKEAENITTGCAEHC	Hex(6)HexNAc(5)Fuc(1)	2133.7716	1079.4573	4
EAENITTGCAEHC	Hex(6)HexNAc(5)NeuAc(1)Fuc(1)	2424.867	1296.8362	3
EAENITTGCAEHC	Hex(6)HexNAc(5)NeuAc(2)Fuc(1)	2715.9624	1393.8673	3
EAENITTGCAEHC	Hex(6)HexNAc(5)NeuAc(3)Fuc(1)	3007.0578	1490.8957	3
EAENITTGCAEHC	Hex(6)HexNAc(6)NeuAc(1)Fuc(1)	2627.9464	1364.5301	3
EAENITTGCAEHC	Hex(7)HexNAc(6)Fuc(1)	2498.9038	1321.5159	3
EAENITTGCAEHC	Hex(7)HexNAc(6)NeuAc(1)Fuc(1)	2789.9992	1064.1627	4
EAENITTGCAEHC	Hex(7)HexNAc(6)NeuAc(2)Fuc(1)	3081.0946	1136.9342	4
EAENITTGCAEHC	Hex(8)HexNAc(7)NeuAc(1)Fuc(1)	3155.1314	1155.4428	4

Table S23. EPO N-glycopeptides (Asn65) identified from 10 μg of EPO standard after in-membrane digestion. This is the analysis of the second replicate solution (Exp#2).

Peptide	Gycan	Glycan Mass (Da)	Precursor m/z	Precursor charge
SLNENITVPDTK	Hex(3)HexNAc(3)Fuc(1)	1241.4544	1286.5711	2
SLNENITVPDTK	Hex(3)HexNAc(4)Fuc(1)	1444.5338	1388.1123	2
SLNENITVPDTKVNFYAWK	Hex(3)HexNAc(5)Fuc(1)	1647.6132	1296.2499	3
SLNENITVPDTK	Hex(4)HexNAc(3)Fuc(1)	1403.5073	1367.6001	2
SLNENITVPDTK	Hex(4)HexNAc(4)Fuc(1)	1606.5866	1469.1323	2
SLNENITVPDTK	Hex(4)HexNAc(4)NeuAc(1)Fuc(1)	1897.682	1076.7938	3
SLNENITVPDTK	Hex(4)HexNAc(5)Fuc(1)	1809.666	1047.4503	3
SLNENITVPDTK	Hex(4)HexNAc(6)NeuAc(1)Fuc(1)	2303.8408	1212.1799	3
SLNENITVPDTK	Hex(5)HexNAc(3)Fuc(1)	1565.5601	1448.6207	2
SLNENITVPDTK	Hex(5)HexNAc(4)Fuc(1)	1768.6394	1550.1659	2
SLNENITVPDTK	Hex(5)HexNAc(4)NeuAc(1)Fuc(1)	2059.7348	1130.8142	3
SLNENITVPDTK	Hex(5)HexNAc(5)Fuc(1)	1971.7188	826.3554	4
SLNENITVPDTK	Hex(5)HexNAc(5)NeuAc(1)Fuc(1)	2262.8142	1198.5021	3
SLNENITVPDTK	Hex(5)HexNAc(6)NeuAc(2)Fuc(1)	2756.989	1363.2312	3
SLNENITVPDTK	Hex(6)HexNAc(4)Fuc(1)	1930.6923	1087.7923	3
SLNENITVPDTK	Hex(6)HexNAc(5)Fuc(1)	2133.7716	866.868	4
SLNENITVPDTK	Hex(6)HexNAc(5)NeuAc(1)Fuc(1)	2424.867	939.644	4
SLNENITVPDTK	Hex(6)HexNAc(6)Fuc(1)	2336.851	1223.1888	3
SLNENITVPDTK	Hex(7)HexNAc(6)Fuc(1)	2498.9038	1915.2984	2
SLNENITVPDTK	Hex(7)HexNAc(6)NeuAc(1)Fuc(1)	2789.9992	1374.2313	3
SLNENITVPDTK	Hex(7)HexNAc(6)NeuAc(2)Fuc(1)	3081.0946	1471.2687	3
SLNENITVPDTK	Hex(7)HexNAc(6)NeuAc(3)Fuc(1)	3372.19	1176.4726	4
SLNENITVPDTK	Hex(7)HexNAc(6)NeuAc(4)Fuc(1)	3663.2854	1249.2443	4

Table S24. EPO N-glycopeptides (Asn110) identified from 10 µg of EPO standard after in-membrane digestion. This is the analysis of the second replicate solution (Exp#2).

Peptide	Glycan	Glycan Mass (Da)	Precursor m/z	Precursor charge
GQALLVNSSQPWEPLQLHVDK AVSGLR	Hex(3)HexNAc(4)Fuc(1)	1444.5338	1097.5356	4
GQALLVNSSQPWEPLQLHVDK	Hex(3)HexNAc(5)Fuc(1)	1647.6132	1002.4735	4
GQALLVNSSQPWEPLQLHVDK	Hex(4)HexNAc(3)Fuc(1)	1403.5073	1254.922	3
GQALLVNSSQPWEPLQLHVDK	Hex(4)HexNAc(3)NeuAc(1) Fuc(1)	1694.6027	1351.9549	3
GQALLVNSSQPWEPLQLHVDK	Hex(4)HexNAc(4)Fuc(1)	1606.5866	1322.6127	3
GQALLVNSSQPWEPLQLHVDK	Hex(4)HexNAc(4)NeuAc(1) Fuc(1)	1897.682	1064.9828	4
GQALLVNSSQPWEPLQLHVDK AVSGLR	Hex(4)HexNAc(5)Fuc(1)	1809.666	1188.8191	4
GQALLVNSSQPWEPLQLHVDK	Hex(4)HexNAc(5)NeuAc(1) Fuc(1)	2100.7614	1115.7565	4
GQALLVNSSQPWEPLQLHVDK	Hex(4)HexNAc(6)Fuc(1)	2012.7453	1457.9969	3
GQALLVNSSQPWEPLQLHVDK	Hex(5)HexNAc(4)Fuc(1)	1768.6394	1376.6316	3
GQALLVNSSQPWEPLQLHVDK	Hex(5)HexNAc(4)NeuAc(1) Fuc(1)	2059.7348	1473.6702	3
GQALLVNSSQPWEPLQLHVDK	Hex(5)HexNAc(4)NeuAc(2) Fuc(1)	2350.8302	1570.6983	3
GQALLVNSSQPWEPLQLHVDK AVSGLR	Hex(5)HexNAc(5)Fuc(1)	1971.7188	1229.3326	4
GQALLVNSSQPWEPLQLHVDK AVSGLR	Hex(5)HexNAc(5)NeuAc(2) Fuc(1)	2553.9096	1100.1043	5
GQALLVNSSQPWEPLQLHVDK	Hex(6)HexNAc(5)Fuc(1)	2133.7716	1124.0083	4
GQALLVNSSQPWEPLQLHVDK AVSGLR	Hex(6)HexNAc(5)NeuAc(1) Fuc(1)	2424.867	1342.6192	4
GQALLVNSSQPWEPLQLHVDK AVSGLR	Hex(6)HexNAc(5)NeuAc(2) Fuc(1)	2715.9624	1132.5158	5
GQALLVNSSQPWEPLQLHVDK	Hex(6)HexNAc(5)NeuAc(3) Fuc(1)	3007.0578	1342.3297	4
GQALLVNSSQPWEPLQLHVDK	Hex(7)HexNAc(6)Fuc(1)	2498.9038	1215.2907	4
GQALLVNSSQPWEPLQLHVDK	Hex(7)HexNAc(6)NeuAc(1) Fuc(1)	2789.9992	859.0452	6
GQALLVNSSQPWEPLQLHVDK	Hex(7)HexNAc(6)NeuAc(2) Fuc(1)	3081.0946	1360.8424	4
GQALLVNSSQPWEPLQLHVDK	Hex(7)HexNAc(6)NeuAc(3) Fuc(1)	3372.19	1147.0924	5
GQALLVNSSQPWEPLQLHVDK	Hex(7)HexNAc(6)NeuAc(4) Fuc(1)	3663.2854	1321.975	5
GQALLVNSSQPWEPLQLHVDK	Hex(8)HexNAc(7)NeuAc(1) Fuc(1)	3155.1314	1379.3503	4
GQALLVNSSQPWEPLQLHVDK	Hex(8)HexNAc(7)NeuAc(3) Fuc(1)	3737.3222	1220.118	5
GQALLVNSSQPWEPLQLHVDK	Hex(8)HexNAc(7)NeuAc(4) Fuc(1)	4028.4176	1278.3388	5

Table S25. EPO N-glycopeptides (Asn51) identified from 10 μg of EPO standard after in-membrane digestion. This is the analysis of the third replicate solution (Exp#3).

Peptide	Glycan	Glycan Mass (Da)	Precursor m/z	Precursor charge
EAENITTGCAEHC	Hex(3)HexNAc(3)Fuc(1)	1241.4544	902.3616	3
EAENITTGCAEHC	Hex(3)HexNAc(4)Fuc(1)	1444.5338	727.7921	4
EAENITTGCAEHC	Hex(3)HexNAc(5)Fuc(1)	1647.6132	1037.747	3
EAENITTGCAEHC	Hex(4)HexNAc(3)Fuc(1)	1403.5073	956.3826	3
EAENITTGCAEHC	Hex(4)HexNAc(3)NeuAc(1)Fuc(1)	1694.6027	1053.4151	3
EAENITTGCAEHC	Hex(4)HexNAc(4)Fuc(1)	1606.5866	1535.6054	2
EAENITTGCAEHC	Hex(4)HexNAc(4)NeuAc(1)Fuc(1)	1897.682	1121.1093	3
EAENITTGCAEHC	Hex(4)HexNAc(5)Fuc(1)	1809.666	1091.7671	3
EAENITTGCAEHC	Hex(4)HexNAc(5)NeuAc(1)Fuc(1)	2100.7614	1188.8014	3
EAENITTGCAEHC	Hex(5)HexNAc(4)Fuc(1)	1768.6394	1078.093	3
EAENITTGCAEHC	Hex(5)HexNAc(4)NeuAc(1)Fuc(1)	2059.7348	1762.1818	2
EAENITTGC	Hex(5)HexNAc(4)NeuAc(2)Fuc(1)	2350.8302	1666.1333	2
EAENITTGCAEHC	Hex(5)HexNAc(5)Fuc(1)	1971.7188	859.5893	4
EAENITTGCAEHC	Hex(5)HexNAc(5)NeuAc(1)Fuc(1)	2262.8142	932.3682	4
EAENITTGCAEHC	Hex(6)HexNAc(5)Fuc(1)	2133.7716	1199.7988	3
EAENITTGCAEHC	Hex(6)HexNAc(5)NeuAc(1)Fuc(1)	2424.867	1296.8334	3
EAENITTGCAEHC	Hex(6)HexNAc(5)NeuAc(2)Fuc(1)	2715.9624	1045.6513	4
EAENITTGCAEHC	Hex(6)HexNAc(5)NeuAc(3)Fuc(1)	3007.0578	1490.9006	3
EAENITTGCAEHC	Hex(7)HexNAc(6)Fuc(1)	2498.9038	991.3832	4
EAENITTGCAEHC	Hex(7)HexNAc(6)NeuAc(1)Fuc(1)	2789.9992	1064.1624	4
EAENITTGCAEHC	Hex(7)HexNAc(6)NeuAc(2)Fuc(1)	3081.0946	1515.5835	3
EAENITTGCAEHC	Hex(7)HexNAc(6)NeuAc(3)Fuc(1)	3372.19	1209.7137	4
EAENITTGCAEHC	Hex(7)HexNAc(6)NeuAc(4)Fuc(1)	3663.2854	1282.4877	4
EAENITTGCAEHC	Hex(8)HexNAc(7)NeuAc(1)Fuc(1)	3155.1314	1155.446	4

Table S26. EPO N-glycopeptides (Asn65) identified from 10 µg of EPO standard after in-membrane digestion. This is the analysis of the third replicate solution (Exp#3).

Peptide	Glycan	Glycan Mass (Da)	Precursor m/z	Precursor charge
SLNENITVPDTK	Hex(3)HexNAc(3)Fuc(1)	1241.4544	858.05	3
SLNENITVPDTK	Hex(3)HexNAc(4)Fuc(1)	1444.5338	1388.1114	2
SLNENITVPDTK	Hex(3)HexNAc(5)Fuc(1)	1647.6132	1489.6529	2
SLNENITVPDTK	Hex(3)HexNAc(6)Fuc(1)	1850.6925	1591.1857	2
SLNENITVPDTK	Hex(4)HexNAc(3)Fuc(1)	1403.5073	1367.6004	2
SLNENITVPDTK	Hex(4)HexNAc(3)NeuAc(1)Fuc(1)	1694.6027	1513.1438	2
SLNENITVPDTKVNFYAWK	Hex(4)HexNAc(4)Fuc(1)	1606.5866	1282.5779	3
SLNENITVPDTK	Hex(4)HexNAc(4)NeuAc(1)Fuc(1)	1897.682	1076.7949	3
SLNENITVPDTK	Hex(4)HexNAc(5)Fuc(1)	1809.666	1047.455	3
SLNENITVPDTK	Hex(4)HexNAc(5)NeuAc(1)Fuc(1)	2100.7614	1144.4837	3
SLNENITVPDTK	Hex(4)HexNAc(6)NeuAc(1)Fuc(1)	2303.8408	1212.1855	3
SLNENITVPDTK	Hex(5)HexNAc(4)Fuc(1)	1768.6394	1550.1663	2
SLNENITVPDTK	Hex(5)HexNAc(4)NeuAc(1)Fuc(1)	2059.7348	1130.8143	3
SLNENITVPDTK	Hex(5)HexNAc(4)NeuAc(2)Fuc(1)	2350.8302	921.1351	4
SLNENITVPDTKVNFYAWK	Hex(5)HexNAc(5)Fuc(1)	1971.7188	1053.4717	4
SLNENITVPDTK	Hex(5)HexNAc(5)NeuAc(1)Fuc(1)	2262.8142	1198.5034	3
SLNENITVPDTK	Hex(5)HexNAc(6)NeuAc(1)Fuc(1)	2465.8936	949.8969	4
SLNENITVPDTK	Hex(6)HexNAc(5)Fuc(1)	2133.7716	1732.7306	2
SLNENITVPDTK	Hex(6)HexNAc(5)NeuAc(1)Fuc(1)	2424.867	1878.2773	2
SLNENITVPDTK	Hex(6)HexNAc(5)NeuAc(2)Fuc(1)	2715.9624	1349.5593	3
SLNENITVPDTK	Hex(6)HexNAc(5)NeuAc(3)Fuc(1)	3007.0578	1446.5909	3
SLNENITVPDTK	Hex(7)HexNAc(6)Fuc(1)	2498.9038	1915.2949	2
SLNENITVPDTK	Hex(7)HexNAc(6)NeuAc(1)Fuc(1)	2789.9992	1374.2292	3
SLNENITVPDTK	Hex(7)HexNAc(6)NeuAc(3)Fuc(1)	3372.19	941.3814	5
SLNENITVPDTK	Hex(7)HexNAc(6)NeuAc(4)Fuc(1)	3663.2854	1665.3328	3

Table S27. EPO N-glycopeptides (Asn110) identified from 10 μg of EPO standard after in-membrane digestion. This is the analysis of the third replicate solution (Exp#3).

Peptide	Glycan	Glycan Mass (Da)	Precursor m/z	Precursor charge
GQALLVNSSQPWEPLQLHVDK	Hex(3)HexNAc(4)Fuc(1)	1444.5338	1268.5966	3
GQALLVNSSQPWEPLQLHVDK	Hex(3)HexNAc(5)Fuc(1)	1647.6132	1336.2872	3
GQALLVNSSQPWEPLQLHVDK	Hex(4)HexNAc(3)Fuc(1)	1403.5073	1254.9208	3
GQALLVNSSQPWEPLQLHVDK	Hex(4)HexNAc(4)Fuc(1)	1606.5866	1322.6144	3
GQALLVNSSQPWEPLQLHVDK	Hex(4)HexNAc(4)NeuAc(1)Fuc(1)	1897.682	1064.9892	4
GQALLVNSSQPWEPLQLHVDK	Hex(4)HexNAc(5)NeuAc(1)Fuc(1)	2100.7614	1115.7588	4
GQALLVNSSQPWEPLQLHVDK	Hex(5)HexNAc(4)Fuc(1)	1768.6394	826.3822	5
GQALLVNSSQPWEPLQLHVDK	Hex(5)HexNAc(4)NeuAc(1)Fuc(1)	2059.7348	1473.6638	3
GQALLVNSSQPWEPLQLHVDK	Hex(5)HexNAc(4)NeuAc(2)Fuc(1)	2350.8302	1178.2729	4
GQALLVNSSQPWEPLQLHVDKAVSGLR	Hex(5)HexNAc(5)Fuc(1)	1971.7188	1229.3293	4
GQALLVNSSQPWEPLQLHVDKAVSGLR	Hex(5)HexNAc(5)NeuAc(1)Fuc(1)	2262.8142	1041.8857	5
GQALLVNSSQPWEPLQLHVDKAVSGLR	Hex(5)HexNAc(5)NeuAc(2)Fuc(1)	2553.9096	1100.1054	5
GQALLVNSSQPWEPLQLHVDK	Hex(6)HexNAc(5)Fuc(1)	2133.7716	899.408	5
GQALLVNSSQPWEPLQLHVDKAVSGLR	Hex(6)HexNAc(5)NeuAc(1)Fuc(1)	2424.867	1074.2978	5
GQALLVNSSQPWEPLQLHVDKAVSGLR	Hex(6)HexNAc(5)NeuAc(2)Fuc(1)	2715.9624	1132.5176	5
GQALLVNSSQPWEPLQLHVDK	Hex(6)HexNAc(6)Fuc(1)	2336.851	1566.0298	3
GQALLVNSSQPWEPLQLHVDK	Hex(6)HexNAc(6)NeuAc(2)Fuc(1)	2919.0418	1760.0926	3
GQALLVNSSQPWEPLQLHVDK (7,Deamidated (+0.984));	Hex(6)HexNAc(6)NeuAc(3)Fuc(1)	3210.1372	1857.4601	3
GQALLVNSSQPWEPLQLHVDKAVSGLR	Hex(7)HexNAc(6)Fuc(1)	2498.9038	1361.1262	4
GQALLVNSSQPWEPLQLHVDK	Hex(7)HexNAc(6)NeuAc(1)Fuc(1)	2789.9992	1288.0642	4
GQALLVNSSQPWEPLQLHVDKAVSGLR	Hex(7)HexNAc(6)NeuAc(2)Fuc(1)	3081.0946	1205.5475	5
GQALLVNSSQPWEPLQLHVDKAVSGLR	Hex(7)HexNAc(6)NeuAc(3)Fuc(1)	3372.19	1263.7621	5
GQALLVNSSQPWEPLQLHVDKAVSGLR	Hex(7)HexNAc(6)NeuAc(4)Fuc(1)	3663.2854	1321.9859	5
GQALLVNSSQPWEPLQLHVDKAVSGLR	Hex(8)HexNAc(7)NeuAc(4)Fuc(1)	4028.4176	1395.0065	5

Table S28. EPO N-glycopeptides (Asn51) identified from 10 μg of EPO spiked into CHO cell supernatant and recovered using the affinity-peptide membrane prior to in-membrane digestion. This is the analysis of the first replicate solution (Exp#1).

Peptide	Glycan	Glycan Mass (Da)	Precursor m/z	Precursor charge
EAENITTGCAEHC	Hex(3)HexNAc(3)Fuc(1)	1241.4544	1353.0462	2
EAENITTGCAEHC	Hex(3)HexNAc(4)Fuc(1)	1444.5338	970.0579	3
EAENITTGCAEHC	Hex(3)HexNAc(5)Fuc(1)	1647.6132	1037.7543	3
EAENITTGC	Hex(4)HexNAc(3)Fuc(1)	1403.5073	1192.4776	2
EAENITTGC	Hex(4)HexNAc(3)NeuAc(1)Fuc(1)	1694.6027	1338.0238	2
EAENITTGCAEHC	Hex(4)HexNAc(4)Fuc(1)	1606.5866	1024.0803	3
EAENITTGC	Hex(4)HexNAc(4)NeuAc(1)Fuc(1)	1897.682	1439.5634	2
EAENITTGCAEHC	Hex(4)HexNAc(5)Fuc(1)	1809.666	1091.7716	3
EAENITTGCAEHC	Hex(4)HexNAc(5)NeuAc(1)Fuc(1)	2100.7614	1188.8004	3
EAENITTGCAEHC	Hex(5)HexNAc(3)Fuc(1)	1565.5601	1010.4014	3
EAENITTGCAEHC	Hex(5)HexNAc(4)Fuc(1)	1768.6394	1078.097	3
EAENITTGCAEHC	Hex(5)HexNAc(4)NeuAc(1)Fuc(1)	2059.7348	1175.1298	3
EAENITTGC	Hex(5)HexNAc(4)NeuAc(2)Fuc(1)	2350.8302	1111.096	3
EAENITTGCAEHC	Hex(5)HexNAc(5)Fuc(1)	1971.7188	1145.7876	3
EAENITTGCAEHC	Hex(5)HexNAc(5)NeuAc(1)Fuc(1)	2262.8142	1242.8175	3
EAENITTGCAEHC	Hex(6)HexNAc(4)Fuc(1)	1930.6923	1132.1068	3
EAENITTGCAEHC	Hex(6)HexNAc(5)Fuc(1)	2133.7716	1199.8097	3
EAENITTGCAEHC	Hex(6)HexNAc(5)NeuAc(1)Fuc(1)	2424.867	1296.8404	3
EAENITTGCAEHC	Hex(6)HexNAc(5)NeuAc(2)Fuc(1)	2715.9624	1393.8729	3
EAENITTGCAEHC	Hex(6)HexNAc(5)NeuAc(3)Fuc(1)	3007.0578	1118.4306	4
EAENITTGCAEHC	Hex(7)HexNAc(6)Fuc(1)	2498.9038	1321.5135	3
EAENITTGCAEHC	Hex(7)HexNAc(6)NeuAc(2)Fuc(1)	3081.0946	1136.9397	4

Table S29. EPO N-glycopeptides (Asn65) identified from 10 µg of EPO spiked into CHO cell supernatant and recovered using the affinity-peptide membrane prior to in-membrane digestion. This is the analysis of the first replicate solution (Exp#1).

Peptide	Glycan	Glycan Mass (Da)	Precursor m/z	Precursor charge
SLNENITVPDTK	Hex(3)HexNAc(3)Fuc(1)	1241.4544	858.0506	3
SLNENITVPDTK	Hex(3)HexNAc(4)Fuc(1)	1444.5338	1388.1189	2
SLNENITVPDTK	Hex(3)HexNAc(5)Fuc(1)	1647.6132	1489.6503	2
SLNENITVPDTK	Hex(3)HexNAc(6)Fuc(1)	1850.6925	1591.1961	2
SLNENITVPDTK	Hex(4)HexNAc(3)Fuc(1)	1403.5073	1367.6038	2
SLNENITVPDTKVNFYAWK	Hex(4)HexNAc(4)Fuc(1)	1606.5866	1282.5773	3
SLNENITVPDTK	Hex(4)HexNAc(5)Fuc(1)	1809.666	1570.6791	2
SLNENITVPDTK	Hex(4)HexNAc(5)NeuAc(1)Fuc(1)	2100.7614	1716.2184	2
SLNENITVPDTK	Hex(5)HexNAc(4)Fuc(1)	1768.6394	1033.7838	3
SLNENITVPDTK	Hex(5)HexNAc(4)NeuAc(1)Fuc(1)	2059.7348	1130.8163	3
SLNENITVPDTK	Hex(5)HexNAc(4)NeuAc(2)Fuc(1)	2350.8302	921.1381	4
SLNENITVPDTK	Hex(5)HexNAc(5)Fuc(1)	1971.7188	1101.4736	3
SLNENITVPDTK	Hex(5)HexNAc(5)NeuAc(1)Fuc(1)	2262.8142	1198.5028	3
SLNENITVPDTK	Hex(5)HexNAc(6)Fuc(1)	2174.7982	1753.2515	2
SLNENITVPDTK	Hex(6)HexNAc(5)Fuc(1)	2133.7716	866.8701	4
SLNENITVPDTK	Hex(6)HexNAc(5)NeuAc(1)Fuc(1)	2424.867	1878.2817	2
SLNENITVPDTK	Hex(6)HexNAc(5)NeuAc(3)Fuc(1)	3007.0578	1085.1937	4
SLNENITVPDTK	Hex(6)HexNAc(6)Fuc(1)	2336.851	1223.1877	3
SLNENITVPDTK	Hex(7)HexNAc(6)Fuc(1)	2498.9038	1915.2961	2
SLNENITVPDTK	Hex(7)HexNAc(6)NeuAc(1)Fuc(1)	2789.9992	1374.2369	3
SLNENITVPDTK	Hex(7)HexNAc(6)NeuAc(2)Fuc(1)	3081.0946	1471.271	3
SLNENITVPDTK	Hex(7)HexNAc(6)NeuAc(3)Fuc(1)	3372.19	1176.4767	4
SLNENITVPDTK	Hex(7)HexNAc(6)NeuAc(4)Fuc(1)	3663.2854	1665.3299	3

Table S30. EPO N-glycopeptides (Asn110) identified from 10 µg of EPO spiked into CHO cell supernatant and recovered using the affinity-peptide membrane prior to in-membrane digestion. This is the analysis of the first replicate solution (Exp#1).

Peptide	Glycan	Glycan Mass (Da)	Precursor m/z	Precursor charge
GQALLVNSSQPWEPLQLHVDKAVSGLR	Hex(3)HexNAc(5)Fuc(1)	1647.6132	1148.3103	4
GQALLVNSSQPWEPLQLHVDK	Hex(4)HexNAc(3)Fuc(1)	1403.5073	1254.9203	3
GQALLVNSSQPWEPLQLHVDK	Hex(4)HexNAc(3)NeuAc(1)Fuc(1)	1694.6027	1351.9569	3
GQALLVNSSQPWEPLQLHVDKAVSGLR	Hex(4)HexNAc(4)Fuc(1)	1606.5866	1138.0539	4
GQALLVNSSQPWEPLQLHVDK	Hex(4)HexNAc(4)NeuAc(1)Fuc(1)	1897.682	1064.9889	4
GQALLVNSSQPWEPLQLHVDKAVSGLR	Hex(4)HexNAc(5)Fuc(1)	1809.666	1188.8232	4
GQALLVNSSQPWEPLQLHVDK	Hex(5)HexNAc(4)Fuc(1)	1768.6394	1376.6344	3
GQALLVNSSQPWEPLQLHVDK	Hex(5)HexNAc(4)NeuAc(1)Fuc(1)	2059.7348	1473.6689	3
GQALLVNSSQPWEPLQLHVDKAVSGLR	Hex(5)HexNAc(4)NeuAc(2)Fuc(1)	2350.8302	1324.1153	4
GQALLVNSSQPWEPLQLHVDK	Hex(5)HexNAc(5)NeuAc(1)Fuc(1)	2262.8142	1156.2738	4
GQALLVNSSQPWEPLQLHVDK	Hex(5)HexNAc(5)NeuAc(2)Fuc(1)	2553.9096	1638.3932	3
GQALLVNSSQPWEPLQLHVDKAVSGLR	Hex(6)HexNAc(4)Fuc(1)	1930.6923	1219.0702	4
GQALLVNSSQPWEPLQLHVDKAVSGLR	Hex(6)HexNAc(5)Fuc(1)	2133.7716	1269.8495	4
GQALLVNSSQPWEPLQLHVDKAVSGLR	Hex(6)HexNAc(5)NeuAc(1)Fuc(1)	2424.867	1074.2991	5
GQALLVNSSQPWEPLQLHVDK	Hex(6)HexNAc(5)NeuAc(2)Fuc(1)	2715.9624	1269.5609	4
GQALLVNSSQPWEPLQLHVDK	Hex(6)HexNAc(6)Fuc(1)	2336.851	1174.7826	4
GQALLVNSSQPWEPLQLHVDKAVSGLR	Hex(7)HexNAc(6)Fuc(1)	2498.9038	1361.1334	4
GQALLVNSSQPWEPLQLHVDKAVSGLR	Hex(7)HexNAc(6)NeuAc(2)Fuc(1)	3081.0946	1205.543	5
GQALLVNSSQPWEPLQLHVDK	Hex(7)HexNAc(6)NeuAc(3)Fuc(1)	3372.19	1147.0957	5
GQALLVNSSQPWEPLQLHVDKAVSGLR	Hex(7)HexNAc(6)NeuAc(4)Fuc(1)	3663.2854	1321.9814	5

Table S31. EPO N-glycopeptides (Asn51) identified from 10 µg of EPO spiked into CHO cell supernatant and recovered using the affinity-peptide membrane prior to in-membrane digestion. This is the analysis of the second replicate solution (Exp#2).

Peptide	Glycan	Glycan Mass (Da)	Precursor m/z	Precursor charge
EAENITTGCAEHC	Hex(3)HexNAc(3)Fuc(1)	1241.4544	677.0259	4
EAENITTGCAEHC	Hex(3)HexNAc(4)Fuc(1)	1444.5338	970.06	3
EAENITTGCAEHC	Hex(3)HexNAc(5)Fuc(1)	1647.6132	1037.7548	3
EAENITTGCAEHC	Hex(4)HexNAc(3)Fuc(1)	1403.5073	717.5408	4
EAENITTGCAEHC	Hex(4)HexNAc(3)NeuAc(1)Fuc(1)	1694.6027	1053.4187	3
EAENITTGCAEHC	Hex(4)HexNAc(4)Fuc(1)	1606.5866	1024.0793	3
EAENITTGCAEHC	Hex(4)HexNAc(4)NeuAc(1)Fuc(1)	1897.682	1121.1106	3
EAENITTGCAEHC	Hex(4)HexNAc(5)Fuc(1)	1809.666	1091.7694	3
EAENITTGCAEHC	Hex(4)HexNAc(5)NeuAc(1)Fuc(1)	2100.7614	1188.8011	3
EAENITTGCAEHC	Hex(5)HexNAc(4)Fuc(1)	1768.6394	1078.0982	3
EAENITTGCAEHC	Hex(5)HexNAc(4)NeuAc(1)Fuc(1)	2059.7348	1762.1877	2
EAENITTGCAEHC	Hex(5)HexNAc(4)NeuAc(2)Fuc(1)	2350.8302	1272.1585	3
EAENITTGCAEHC	Hex(5)HexNAc(5)Fuc(1)	1971.7188	1145.7892	3
EAENITTGCAEHC	Hex(5)HexNAc(5)NeuAc(1)Fuc(1)	2262.8142	1242.8226	3
EAENITTGCAEHC	Hex(6)HexNAc(4)Fuc(1)	1930.6923	1132.1152	3
EAENITTGCAEHC	Hex(6)HexNAc(5)NeuAc(1)Fuc(1)	2424.867	778.5054	5
EAENITTGCAEHC	Hex(6)HexNAc(5)NeuAc(2)Fuc(1)	2715.9624	1393.8723	3
EAENITTGCAEHC	Hex(6)HexNAc(5)NeuAc(3)Fuc(1)	3007.0578	1118.431	4
EAENITTGCAEHC	Hex(6)HexNAc(6)Fuc(1)	2336.851	950.875	4
EAENITTGCAEHC	Hex(6)HexNAc(6)NeuAc(1)Fuc(1)	2627.9464	1364.5274	3
EAENITTGCAEHC	Hex(7)HexNAc(6)Fuc(1)	2498.9038	991.3911	4
EAENITTGCAEHC	Hex(7)HexNAc(6)NeuAc(1)Fuc(1)	2789.9992	1064.1638	4

Table S32. EPO N-glycopeptides (Asn65) identified from 10 µg of EPO spiked into CHO cell supernatant and recovered using the affinity-peptide membrane prior to in-membrane digestion. This is the analysis of the second replicate solution (Exp#2).

Peptide	Glycan	Glycan Mass (Da)	Precursor m/z	Precursor charge
SLNENITVPDTK	Hex(3)HexNAc(3)Fuc(1)	1241.4544	1286.5731	2
SLNENITVPDTK	Hex(3)HexNAc(4)Fuc(1)	1444.5338	1388.1157	2
SLNENITVPDTK	Hex(3)HexNAc(5)Fuc(1)	1647.6132	1489.6552	2
SLNENITVPDTK	Hex(3)HexNAc(6)Fuc(1)	1850.6925	1591.2001	2
SLNENITVPDTK	Hex(4)HexNAc(3)Fuc(1)	1403.5073	1367.6023	2
SLNENITVPDTK	Hex(4)HexNAc(4)Fuc(1)	1606.5866	1469.1408	2
SLNENITVPDTK	Hex(4)HexNAc(4)NeuAc(1)Fuc(1)	1897.682	807.8491	4
SLNENITVPDTK	Hex(4)HexNAc(5)Fuc(1)	1809.666	1570.6786	2
SLNENITVPDTK	Hex(5)HexNAc(4)Fuc(1)	1768.6394	1033.7838	3
SLNENITVPDTK	Hex(5)HexNAc(4)NeuAc(1)Fuc(1)	2059.7348	1130.8163	3
SLNENITVPDTK	Hex(5)HexNAc(5)Fuc(1)	1971.7188	1101.4733	3
SLNENITVPDTK	Hex(5)HexNAc(6)NeuAc(1)Fuc(1)	2465.8936	1266.2	3
SLNENITVPDTK	Hex(6)HexNAc(5)Fuc(1)	2133.7716	866.8723	4
SLNENITVPDTK	Hex(6)HexNAc(5)NeuAc(1)Fuc(1)	2424.867	1878.2833	2
SLNENITVPDTK	Hex(6)HexNAc(5)NeuAc(2)Fuc(1)	2715.9624	1012.4216	4
SLNENITVPDTK	Hex(7)HexNAc(6)Fuc(1)	2498.9038	1277.1998	3
SLNENITVPDTK	Hex(7)HexNAc(6)NeuAc(1)Fuc(1)	2789.9992	1374.2358	3
SLNENITVPDTK	Hex(7)HexNAc(6)NeuAc(2)Fuc(1)	3081.0946	883.1638	5
SLNENITVPDTK	Hex(7)HexNAc(6)NeuAc(4)Fuc(1)	3663.2854	1249.2515	4

Table S33. EPO N-glycopeptides (Asn110) identified from 10 μg of EPO spiked into CHO cell supernatant and recovered using the affinity-peptide membrane prior to in-membrane digestion. This is the analysis of the second replicate solution (Exp#2).

peptide	glycan	Glycan Mass (Da)	Precursor m/z	Precursor charge
GQALLVNSSQPWEPLQLHVDK	Hex(3)HexNAc(4)Fuc(1)	1444.5338	951.6989	4
GQALLVNSSQPWEPLQLHVDK	Hex(3)HexNAc(5)Fuc(1)	1647.6132	1336.2913	3
GQALLVNSSQPWEPLQLHVDK	Hex(4)HexNAc(3)Fuc(1)	1403.5073	1254.9262	3
GQALLVNSSQPWEPLQLHVDK	Hex(4)HexNAc(4)Fuc(1)	1606.5866	1322.6154	3
GQALLVNSSQPWEPLQLHVDK	Hex(4)HexNAc(4)NeuAc(1)Fuc(1)	1897.682	1419.6496	3
GQALLVNSSQPWEPLQLHVDKAVSGLR	Hex(4)HexNAc(5)NeuAc(1)Fuc(1)	2100.7614	1261.5975	4
GQALLVNSSQPWEPLQLHVDK	Hex(5)HexNAc(4)Fuc(1)	1768.6394	1032.7295	4
GQALLVNSSQPWEPLQLHVDKAVSGLR	Hex(5)HexNAc(4)NeuAc(1)Fuc(1)	2059.7348	1251.3412	4
GQALLVNSSQPWEPLQLHVDK	Hex(5)HexNAc(4)NeuAc(2)Fuc(1)	2350.8302	1178.2775	4
GQALLVNSSQPWEPLQLHVDKAVSGLR	Hex(5)HexNAc(5)NeuAc(2)Fuc(1)	2553.9096	1100.1099	5
GQALLVNSSQPWEPLQLHVDK	Hex(5)HexNAc(6)Fuc(1)	2174.7982	1134.269	4
GQALLVNSSQPWEPLQLHVDK	Hex(6)HexNAc(4)Fuc(1)	1930.6923	1430.6427	3
GQALLVNSSQPWEPLQLHVDKAVSGLR	Hex(6)HexNAc(5)Fuc(1)	2133.7716	1016.0799	5
GQALLVNSSQPWEPLQLHVDKAVSGLR	Hex(6)HexNAc(5)NeuAc(1)Fuc(1)	2424.867	1074.2999	5
GQALLVNSSQPWEPLQLHVDKAVSGLR	Hex(6)HexNAc(5)NeuAc(2)Fuc(1)	2715.9624	943.9288	6
GQALLVNSSQPWEPLQLHVDKAVSGLR	Hex(6)HexNAc(5)NeuAc(3)Fuc(1)	3007.0578	1190.7399	5
GQALLVNSSQPWEPLQLHVDKAVSGLR	Hex(6)HexNAc(6)NeuAc(1)Fuc(1)	2627.9464	1393.3948	4
GQALLVNSSQPWEPLQLHVDK	Hex(7)HexNAc(6)Fuc(1)	2498.9038	1620.057	3
GQALLVNSSQPWEPLQLHVDK	Hex(7)HexNAc(6)NeuAc(1)Fuc(1)	2789.9992	1288.0715	4
GQALLVNSSQPWEPLQLHVDKAVSGLR	Hex(7)HexNAc(6)NeuAc(2)Fuc(1)	3081.0946	1205.547	5
GQALLVNSSQPWEPLQLHVDKAVSGLR	Hex(7)HexNAc(6)NeuAc(3)Fuc(1)	3372.19	1263.7615	5
GQALLVNSSQPWEPLQLHVDKAVSGLR	Hex(7)HexNAc(6)NeuAc(4)Fuc(1)	3663.2854	1321.9844	5

Table S34. EPO N-glycopeptides (Asn51) identified from 10 µg of EPO spiked into CHO cell supernatant and recovered using the affinity-peptide membrane prior to in-membrane digestion. This is the analysis of the third replicate solution (Exp#3).

Peptide	Gycan	Glycan Mass (Da)	Precursor m/z	Precursor charge
EAENITTGCAEHC	Hex(3)HexNAc(3)Fuc(1)	1241.4544	902.3685	3
EAENITTGCAEHC	Hex(3)HexNAc(4)Fuc(1)	1444.5338	727.7954	4
EAENITTGCAEHC	Hex(3)HexNAc(5)Fuc(1)	1647.6132	1037.754	3
EAENITTGCAEHC	Hex(4)HexNAc(3)Fuc(1)	1403.5073	717.5382	4
EAENITTGC	Hex(4)HexNAc(3)NeuAc(1)Fuc(1)	1694.6027	1338.0239	2
EAENITTGCAEHC	Hex(4)HexNAc(4)Fuc(1)	1606.5866	1535.614	2
EAENITTGCAEHC	Hex(4)HexNAc(5)Fuc(1)	1809.666	1091.772	3
EAENITTGCAEHC	Hex(4)HexNAc(5)NeuAc(1)Fuc(1)	2100.7614	1188.8032	3
EAENITTGCAEHC	Hex(5)HexNAc(3)Fuc(1)	1565.5601	1010.4034	3
EAENITTGCAEHC	Hex(5)HexNAc(4)Fuc(1)	1768.6394	1616.6417	2
EAENITTGCAEHC	Hex(5)HexNAc(4)NeuAc(1)Fuc(1)	2059.7348	1175.1306	3
EAENITTGCAEHC	Hex(5)HexNAc(4)NeuAc(2)Fuc(1)	2350.8302	1272.1605	3
EAENITTGCAEHC	Hex(5)HexNAc(5)Fuc(1)	1971.7188	1145.7895	3
EAENITTGCAEHC	Hex(5)HexNAc(5)NeuAc(1)Fuc(1)	2262.8142	1242.8237	3
EAENITTGC	Hex(5)HexNAc(6)Fuc(1)	2174.7982	1052.4206	3
EAENITTGCAEHC	Hex(6)HexNAc(4)Fuc(1)	1930.6923	1132.1122	3
EAENITTGCAEHC	Hex(6)HexNAc(5)Fuc(1)	2133.7716	900.1086	4
EAENITTGCAEHC	Hex(6)HexNAc(5)NeuAc(1)Fuc(1)	2424.867	1296.8381	3
EAENITTGC	Hex(6)HexNAc(5)NeuAc(2)Fuc(1)	2715.9624	1232.8102	3
EAENITTGC	Hex(6)HexNAc(5)NeuAc(3)Fuc(1)	3007.0578	997.6312	4
EAENITTGCAEHC	Hex(7)HexNAc(6)Fuc(1)	2498.9038	991.3893	4
EAENITTGCAEHC	Hex(7)HexNAc(6)NeuAc(1)Fuc(1)	2789.9992	1418.5477	3

Table S35. EPO N-glycopeptides (Asn65) identified from 10 µg of EPO spiked into CHO cell supernatant and recovered using the affinity-peptide membrane prior to in-membrane digestion. This is the analysis of the third replicate solution (Exp#3).

Peptide	Gycan	Glycan Mass (Da)	Precursor m/z	Precursor charge
SLNENITVPDTK	Hex(3)HexNAc(3)Fuc(1)	1241.4544	1286.5762	2
SLNENITVPDTK	Hex(3)HexNAc(4)Fuc(1)	1444.5338	1388.1166	2
SLNENITVPDTK	Hex(3)HexNAc(5)Fuc(1)	1647.6132	1489.6496	2
SLNENITVPDTK	Hex(3)HexNAc(6)Fuc(1)	1850.6925	1061.1283	3
SLNENITVPDTK	Hex(4)HexNAc(3)Fuc(1)	1403.5073	1367.6047	2
SLNENITVPDTK	Hex(4)HexNAc(4)Fuc(1)	1606.5866	1469.144	2
SLNENITVPDTK	Hex(4)HexNAc(4)NeuAc(1)Fuc(1)	1897.682	1076.7986	3
SLNENITVPDTK	Hex(4)HexNAc(5)Fuc(1)	1809.666	1047.4571	3
SLNENITVPDTK	Hex(4)HexNAc(5)NeuAc(1)Fuc(1)	2100.7614	858.6203	4
SLNENITVPDTK	Hex(5)HexNAc(4)Fuc(1)	1768.6394	1550.166	2
SLNENITVPDTK	Hex(5)HexNAc(4)NeuAc(1)Fuc(1)	2059.7348	1695.7187	2
SLNENITVPDTK	Hex(5)HexNAc(4)NeuAc(2)Fuc(1)	2350.8302	1841.2579	2
SLNENITVPDTK	Hex(5)HexNAc(5)Fuc(1)	1971.7188	1651.7084	2
SLNENITVPDTK	Hex(6)HexNAc(4)Fuc(1)	1930.6923	1631.1943	2
SLNENITVPDTK	Hex(6)HexNAc(5)Fuc(1)	2133.7716	866.8721	4
SLNENITVPDTK	Hex(6)HexNAc(5)NeuAc(1)Fuc(1)	2424.867	1878.2846	2
SLNENITVPDTK	Hex(6)HexNAc(5)NeuAc(2)Fuc(1)	2715.9624	810.138	5
SLNENITVPDTK	Hex(6)HexNAc(5)NeuAc(3)Fuc(1)	3007.0578	1446.5852	3
SLNENITVPDTK	Hex(7)HexNAc(6)Fuc(1)	2498.9038	1277.1987	3
SLNENITVPDTK	Hex(7)HexNAc(6)NeuAc(1)Fuc(1)	2789.9992	1030.9284	4
SLNENITVPDTK	Hex(7)HexNAc(6)NeuAc(2)Fuc(1)	3081.0946	1103.703	4
SLNENITVPDTK	Hex(7)HexNAc(6)NeuAc(3)Fuc(1)	3372.19	1568.3032	3
SLNENITVPDTK	Hex(7)HexNAc(6)NeuAc(4)Fuc(1)	3663.2854	1665.334	3

Table S36. EPO N-glycopeptides (Asn110) identified from 10 µg of EPO spiked into CHO cell supernatant and recovered using the affinity-peptide membrane prior to in-membrane digestion. This is the analysis of the third replicate solution (Exp#3).

Peptide	Glycan	Glycan Mass (Da)	Precursor m/z	Precursor charge
GQALLVNSSQPWEPLQLHVDK	Hex(3)HexNAc(5)Fuc(1)	1647.6132	802.179	5
GQALLVNSSQPWEPLQLHVDK	Hex(4)HexNAc(3)Fuc(1)	1403.5073	1254.9204	3
GQALLVNSSQPWEPLQLHVDK	Hex(4)HexNAc(3)NeuAc(1)Fuc(1)	1694.6027	1351.9573	3
GQALLVNSSQPWEPLQLHVDK	Hex(4)HexNAc(4)Fuc(1)	1606.5866	992.2169	4
GQALLVNSSQPWEPLQLHVDK	Hex(4)HexNAc(4)NeuAc(1)Fuc(1)	1897.682	1064.9911	4
GQALLVNSSQPWEPLQLHVDK	Hex(5)HexNAc(4)Fuc(1)	1768.6394	1376.6373	3
GQALLVNSSQPWEPLQLHVDK	Hex(5)HexNAc(4)NeuAc(1)Fuc(1)	2059.7348	1105.5048	4
GQALLVNSSQPWEPLQLHVDK	Hex(5)HexNAc(4)NeuAc(2)Fuc(1)	2350.8302	1178.2767	4
GQALLVNSSQPWEPLQLHVDK	Hex(5)HexNAc(5)NeuAc(2)Fuc(1)	2553.9096	1638.3952	3
GQALLVNSSQPWEPLQLHVDK	Hex(5)HexNAc(6)NeuAc(1)Fuc(1)	2465.8936	1207.0429	4
GQALLVNSSQPWEPLQLHVDKAVSGLR	Hex(6)HexNAc(5)Fuc(1)	2133.7716	1269.8495	4
GQALLVNSSQPWEPLQLHVDKAVSGLR	Hex(6)HexNAc(5)NeuAc(1)Fuc(1)	2424.867	1074.2996	5
GQALLVNSSQPWEPLQLHVDK	Hex(6)HexNAc(5)NeuAc(2)Fuc(1)	2715.9624	1269.5609	4
GQALLVNSSQPWEPLQLHVDK	Hex(6)HexNAc(6)NeuAc(1)Fuc(1)	2627.9464	1247.5561	4
GQALLVNSSQPWEPLQLHVDK	Hex(7)HexNAc(6)Fuc(1)	2498.9038	1620.06	3
GQALLVNSSQPWEPLQLHVDK	Hex(7)HexNAc(6)NeuAc(2)Fuc(1)	3081.0946	1360.8443	4
GQALLVNSSQPWEPLQLHVDK	Hex(7)HexNAc(6)NeuAc(3)Fuc(1)	3372.19	1433.6168	4
GQALLVNSSQPWEPLQLHVDK	Hex(7)HexNAc(6)NeuAc(4)Fuc(1)	3663.2854	1205.3151	5
GQALLVNSSQPWEPLQLHVDK	Hex(8)HexNAc(7)NeuAc(3)Fuc(1)	3737.3222	1220.1202	5
GQALLVNSSQPWEPLQLHVDK	Hex(8)HexNAc(7)NeuAc(4)Fuc(1)	4028.4176	1278.3355	5

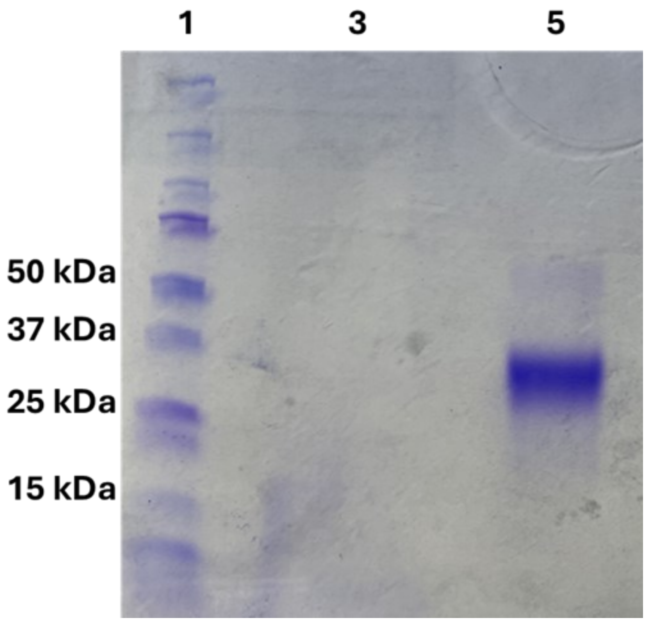

Figure S14. SDS–PAGE analysis of EPO before and after in-membrane tryptic digestion. Lane 1: molecular weight markers; Lane 3: 10 µg of EPO after in-membrane tryptic digestion; Lane 5: 10 µg of EPO passed through a non–trypsin-containing membrane.

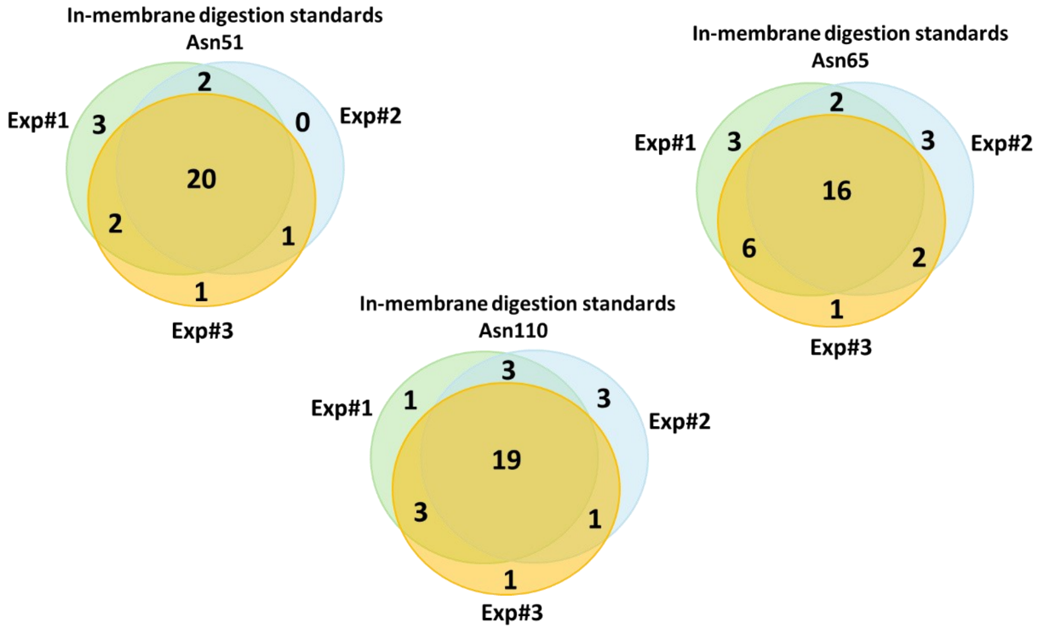

Figure S15. Overlap of glycans identified from EPO standards after three replicate in-membrane tryptic digestions.

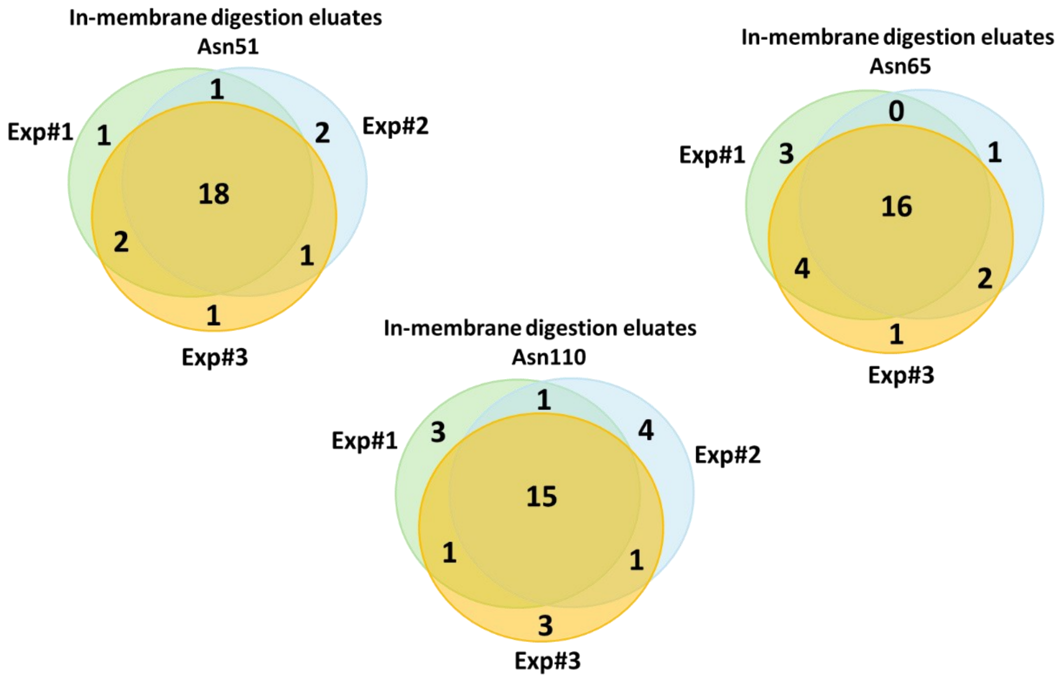

Figure S16. Overlap of glycans identified from EPO recovered using three different affinity membranes prior to three independent in-membrane tryptic digestions.

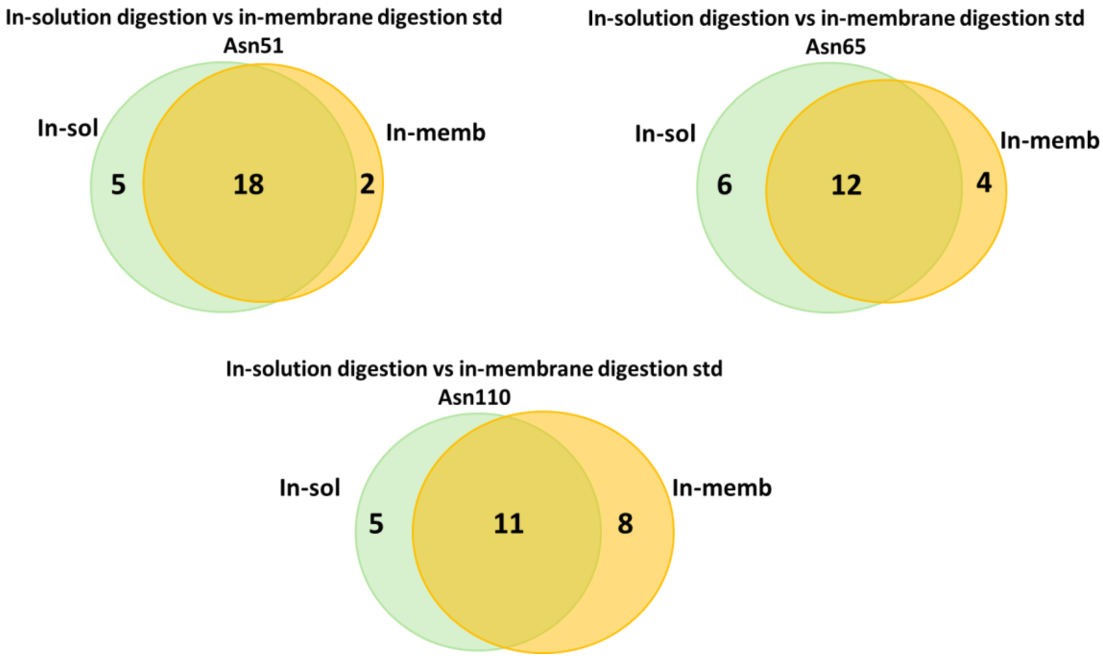

Figure S17. Comparison of EPO glycans identified from 10 µg of EPO standard digested either in solution or in a membrane. Each group includes three replicates, and only glycans detected in all replicates are shown.

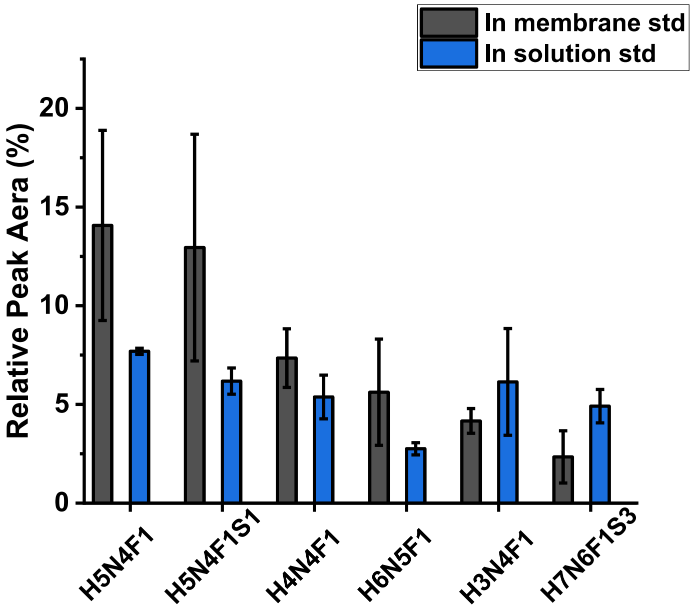

Figure S18. Relative peak areas of the six most intense glycans found at all three N-glycosylation sites for in-membrane and in-solution tryptic digested EPO standards. The peak areas are summed from all glycopeptides containing the glycan. Error bars represent standard deviations from three replicates. Abbreviations: H, hexose; N, N-acetylhexosamine; F, fucose; S, N-acetylneuraminic acid.

Reference:

[1] W. Cao, M.L. Bruening, Analysis of Protein Glycosylation after Rapid Digestion Using Protease-Containing Membranes in Spin Columns, J. Am. Soc. Mass Spectrom. 34 (2023) 1086–1095. https://doi.org/10.1021/jasms.3c00038.

[2] S. Lippold, A. Büttner, M.S.F. Choo, M. Hook, C.J. de Jong, T. Nguyen-Khuong, M. Haberger, D. Reusch, M. Wuhrer, N. de Haan, Cysteine Aminoethylation Enables the Site-Specific Glycosylation Analysis of Recombinant Human Erythropoietin using Trypsin, Anal. Chem. 92 (2020) 9476–9481. https://doi.org/10.1021/acs.analchem.0c01794.
